# Design and Synthesis of Conformationally Diverse Pyrimidine-Embedded Medium/Macro- and Bridged Cycles *via* Skeletal Transformation

**DOI:** 10.3389/fchem.2022.841250

**Published:** 2022-04-04

**Authors:** Yoona Choi, Subin Lee, Heejun Kim, Seung Bum Park

**Affiliations:** CRI Center for Chemical Proteomics, Department of Chemistry, Seoul National University, Seoul, South Korea

**Keywords:** pyrimidine, conformational diversity, skeletal transformation, polyheterocycle, medium cycle, bridged cycle, macrocycle

## Abstract

The rigidity and flexibility of small molecules are complementary in 3-dimensional ligand-protein interaction. Therefore, the chemical library with conformational diversity would be a valuable resource for investigating the influence of skeletal flexibility on the biological system. In this regard, we designed and synthesized ten conformationally diverse pyrimidine-embedded medium/macro- and bridged cyclic scaffolds covering 7- to 14-member rings *via* an efficient skeletal transformation strategy. Their high conformational and shape diversity was confirmed by chemoinformatic analysis.

## Introduction

The construction of small-molecule libraries with high skeletal diversity and biological relevancy is an invaluable resource to discover new therapeutics and chemical modulators (Dandapani and Marcaurelle, 2010; Galloway et al., 2010; O’ Connor et al., 2011). In this respect, we have developed a privileged substructure-based diversity-oriented synthesis (pDOS) strategy over the last decade. The pDOS strategy focuses on creatively reconstructing heterocyclic moieties around the biologically relevant privileged substructures through diversity-generating reactions efficiently (Oh and Park, 2011; Kim et al., 2014). Continuing our endeavor to develop novel pDOS pathways, we recently focused on pyrimidine as a valuable privileged substructure since pyrimidine and its analogs have been extensively used as nucleotide analogs. Although pyrimidine is a well-known molecular framework ensuring good bioactivity, previously reported pyrimidine-containing compounds are mostly monocycles or flat bicycles with limited structural diversity (Wakeling et al., 2002; Yaziji et al., 2011; Lawrence et al., 2012). To overcome this structural limitation, we have developed several pDOS pathways to construct unprecedented pyrimidine-embedded polyheterocycles (Kim et al., 2013; Kim et al., 2016; Choi et al., 2019) and identified a list of small-molecule modulators controlling the cellular contents of lipid droplets (Choi et al., 2015), inhibiting the protein-protein interaction (Kim et al., 2016), and regulating tau proteostasis (Shin et al., 2021).

This report proposed a new pDOS strategy for pyrimidine-containing polyheterocycles with conformational diversity. Either rigid or flexible conformation of small molecules can be critical for specific modulation of 3-dimensional ligand-protein interactions. As the overall orientation of crucial substituents can be pre-organized in the conformationally constrained molecular skeletons, they might exhibit specific interaction toward biological targets with high affinity and selectivity. On the other hand, flexible molecules of which different conformations exist in equilibrium enable distinct binding modes for different proteins, which rigid molecules cannot achieve (Becker et al., 2000; Rubin and Qvit, 2016). In this regard, the chemical library with diverse skeletal flexibility would be a valuable resource for chemical biology and drug discovery community to investigate the influences of skeletal flexibility on the biological system.

**FIGURE 1:**
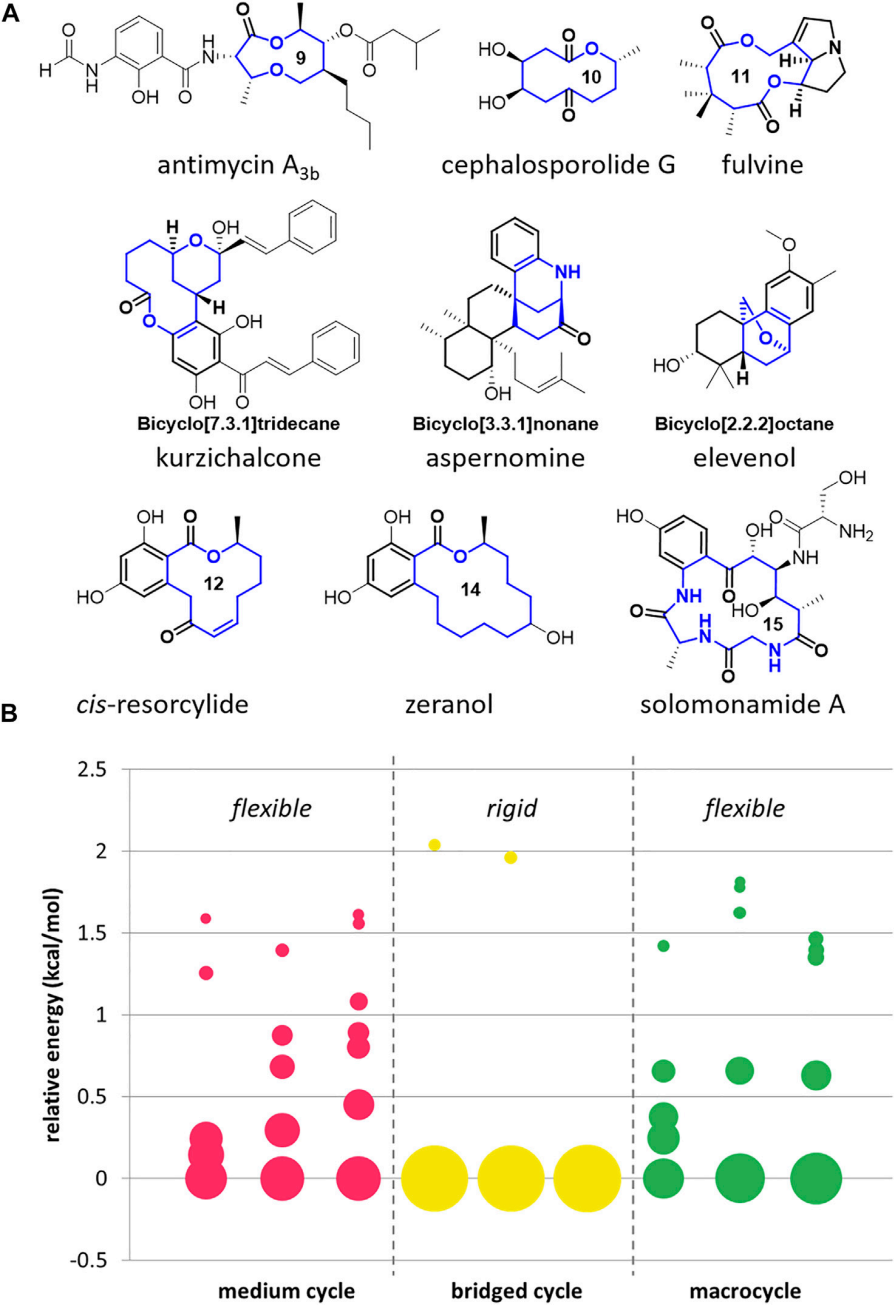
Examples of medium/macro- and bridged rings in bioactive natural products **(A)** and conformational analysis of their core skeletons **(B)**.

Although bioactive medium/macro- and bridged cyclic scaffolds are frequently found in many natural products with conformational diversity (Figure 1A), they are scarce in the reported chemical libraries due to the limited synthetic accessibility (Gradillas and Pérez-Castells, 2006; Shiina, 2007; Hussain et al., 2014; Cheng-Sánchez et al., 2018; Mu et al., 2020). To visualize the conformational diversity of core scaffolds, we conducted a conformational analysis of bioactive natural products shown in Figure 1A. Among the searched conformers of these natural products containing 8- to 15- member rings, we selected the conformers of which Boltzmann population is higher than 2.5% at room temperature and displayed the distribution of the chosen conformers on the bubble chart (Figure 1B, please see the Supplementary Material for detailed search method and raw data in a tabular form). The size of the circle represents the population of the corresponding conformer. As a result, the natural products containing medium-sized rings and macrocycles are relatively flexible with various low-energy conformations. On the other hand, the natural products with bridged cycles are more constrained with a lower number of possible conformers. Although these natural products show apparent conformational diversity, it is impossible to compare their biological activities in response to their skeletal flexibility because these molecules are not directly comparable with different structures.

**FIGURE 2:**
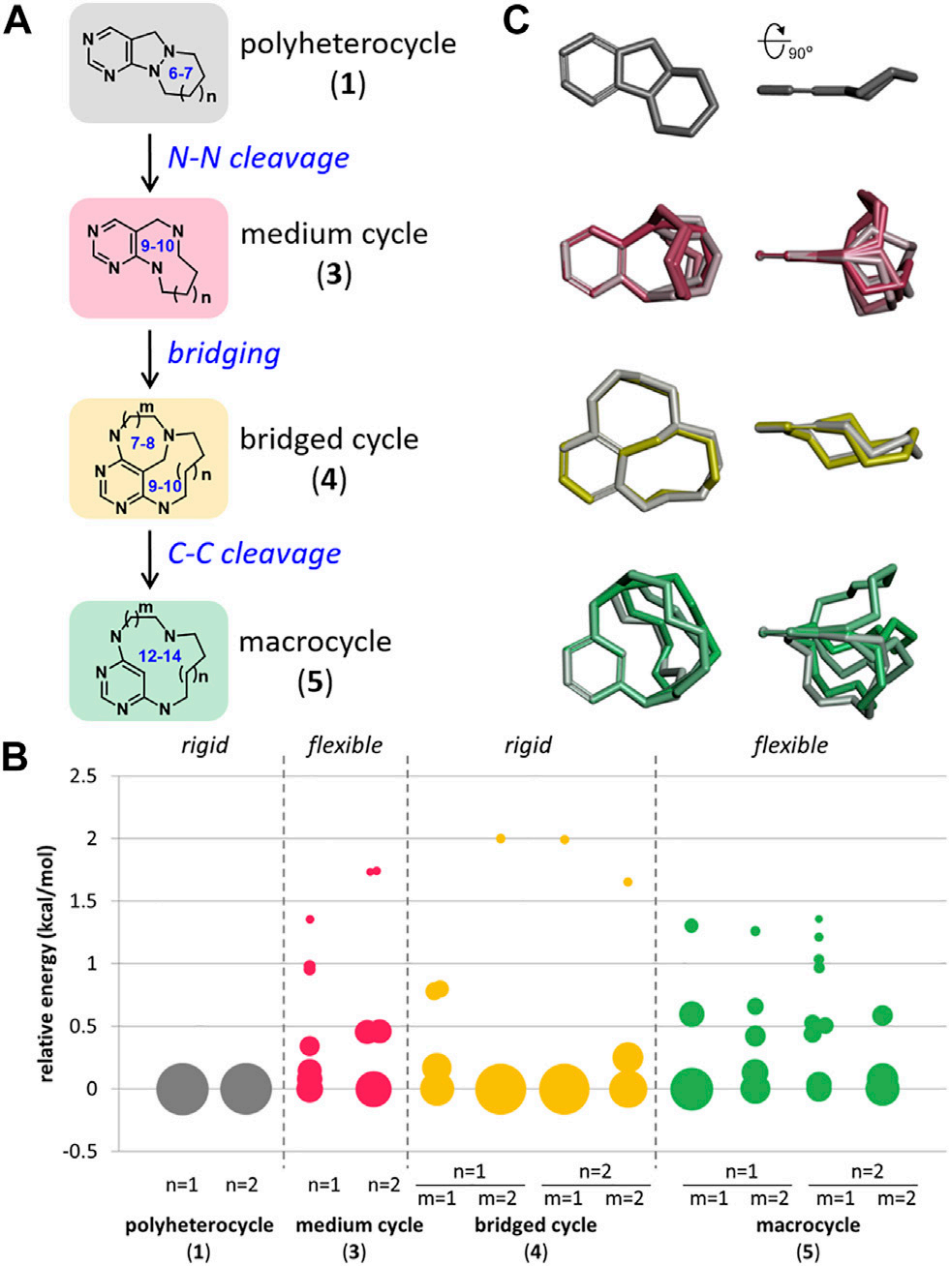
Design strategy for pyrimidine-embedded medium/macro- and bridged cyclic scaffolds. **(A)** Overview of skeletal transformation strategy. **(B)** Conformational analysis of all designed scaffolds. **(C)** Overlay of the selected conformers of representative scaffolds aligned by pyrimidine substructure.

To construct a set of small molecules that allows comparable skeletal flexibility, we paid attention to medium/macro- and bridged cyclic scaffolds sharing similar structural motifs. Therefore, we proposed an efficient skeletal transformation strategy encompassing medium/macro- and bridged rings (Figure 2A). The rigid polyheterocyclic starting scaffolds are composed of 6,5,6- and 6,5,7-membered tricyclic rings (**1**) that can be easily accessed from the reaction of functionalized 4-formyl pyrimidines with cyclic hydrazines. The cleavage of the central N–N bond in azatricycles (**1**) would afford relatively flexible 9- and 10-membered medium-sized rings (**3**). The subsequent intramolecular cyclization would convert them to conformationally restricted bridged cyclic skeletons (**4**). The resulting bridged cycles can be converted to conformationally labile 12- to 14-membered macrocycles (**5**) *via* selective C–C bond cleavage. The most significant advantage of this skeletal transformation strategy is that all respective products for each scaffold can be used as the starting materials for the following transformation. Therefore, we do not need to prepare the intermediate separately.

Before exploring each scaffold, we conducted a conformational analysis to investigate the skeletal flexibility of each scaffold as we did for the representative natural products in Figure 1B. As shown in Figure 2B, initial azatricycles (**1**) and bridged cyclic skeletons (**4**) were conformationally restricted in a single or a small number of possible conformations (see the Supplementary Material for the raw data in a tabular form). On the other hand, the medium-sized rings (**3**) and macrocycles (**5**) showed relative skeletal flexibility with multiple low-energy conformers depending on the ring size. The conformational flexibility of each skeleton was also visualized through the overlay of selected conformers aligned by the pyrimidine moiety (Figure 2C). From this chemoinformatic analysis, we envisioned that this set of polyheterocycles sharing pyrimidine moiety with conformational diversity could be a valuable resource to investigate how skeletal flexibility influences their biological functions.

## Result and Discussion

### Design of Synthetic Routes for Medium/Macro- and Bridged Cyclic Skeletons

**FIGURE 3:**
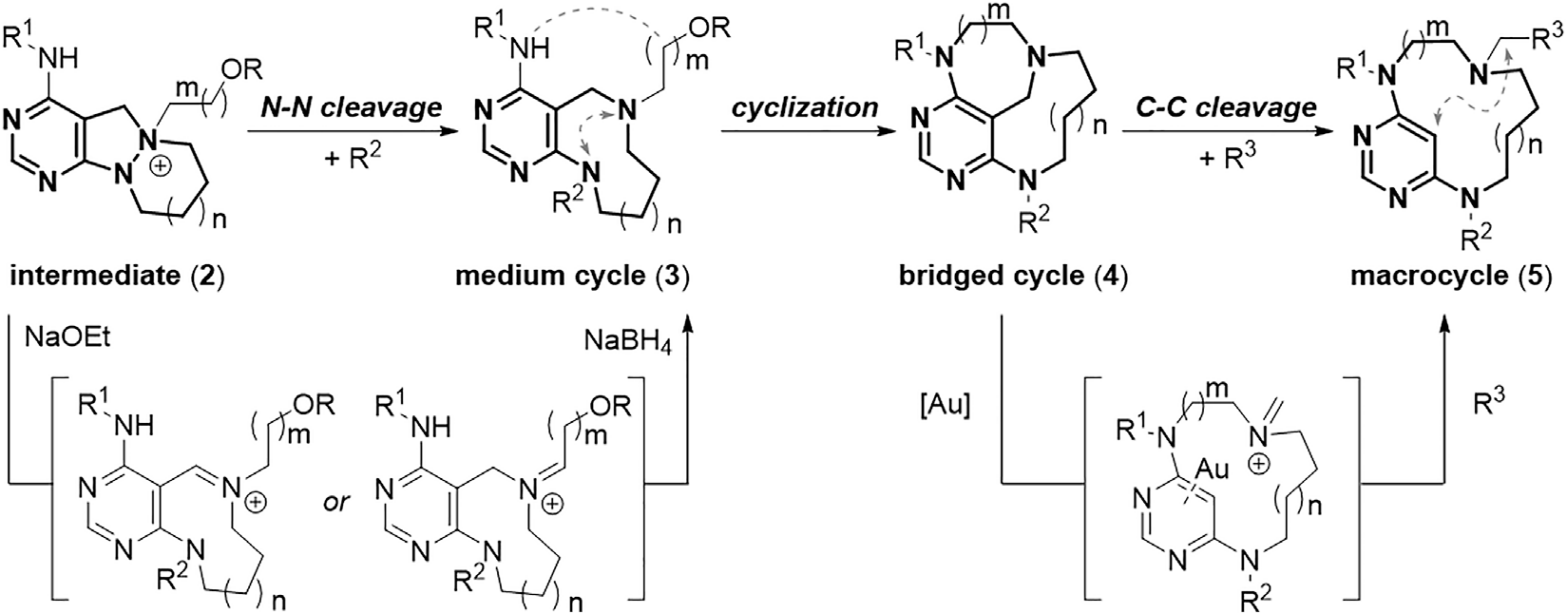
Overall synthetic route for pyrimidine-embedded medium/macro- and bridged cycles.

To access the pyrimidine-embedded medium/macro- and bridged cyclic compounds with sufficient conformational diversity, we first designed conformationally rigid starting tricycles (**1**) that were easily accessible through coupling of functionalized pyrimidines with cyclic hydrazines. In the preparation of starting tricycles, we could secure both the appendage diversity and skeletal diversity by introducing the R^1^ group and varying the ring size of cyclic hydrazines, respectively. The resulting 6,5,6- and 6,5,7-azatricycles (**1**) can become key intermediates (**2**) by selective *N-*quaternization of their tertiary amines (Figure 3, see the Supplementary Material for detailed procedure). These key intermediates (**2**) were then treated with base and hydride sources under N–N bond cleavage conditions (Choi et al., 2019). Base-promoted N–N bond cleavage and hydride-mediated neutralization afforded flexible 9- and 10-membered medium-sized rings (**3**), and we can secure additional appendage diversity by introducing the *R* ^2^ group. Intramolecular cyclization of medium cycles (**3**) would generate conformationally constrained bridged cyclic scaffolds (**4**) with varying chain lengths. Finally, the unconventional gold-catalyzed C–C bond cleavage reaction between the carbon at the 5-position of the pyrimidine ring and the benzylic carbon (Koo et al., 2017) would lead to the formation of ring-expanded flexible macrocycles (**5**) with appendage diversity point at the R^3^ position. Consequently, this skeletal transformation strategy would procure a distinct collection of pyrimidine-embedded medium/macro- and bridged cyclic molecules with skeletal and conformational diversity.

### Exploration for Each Scaffold

**SCHEME 1:**
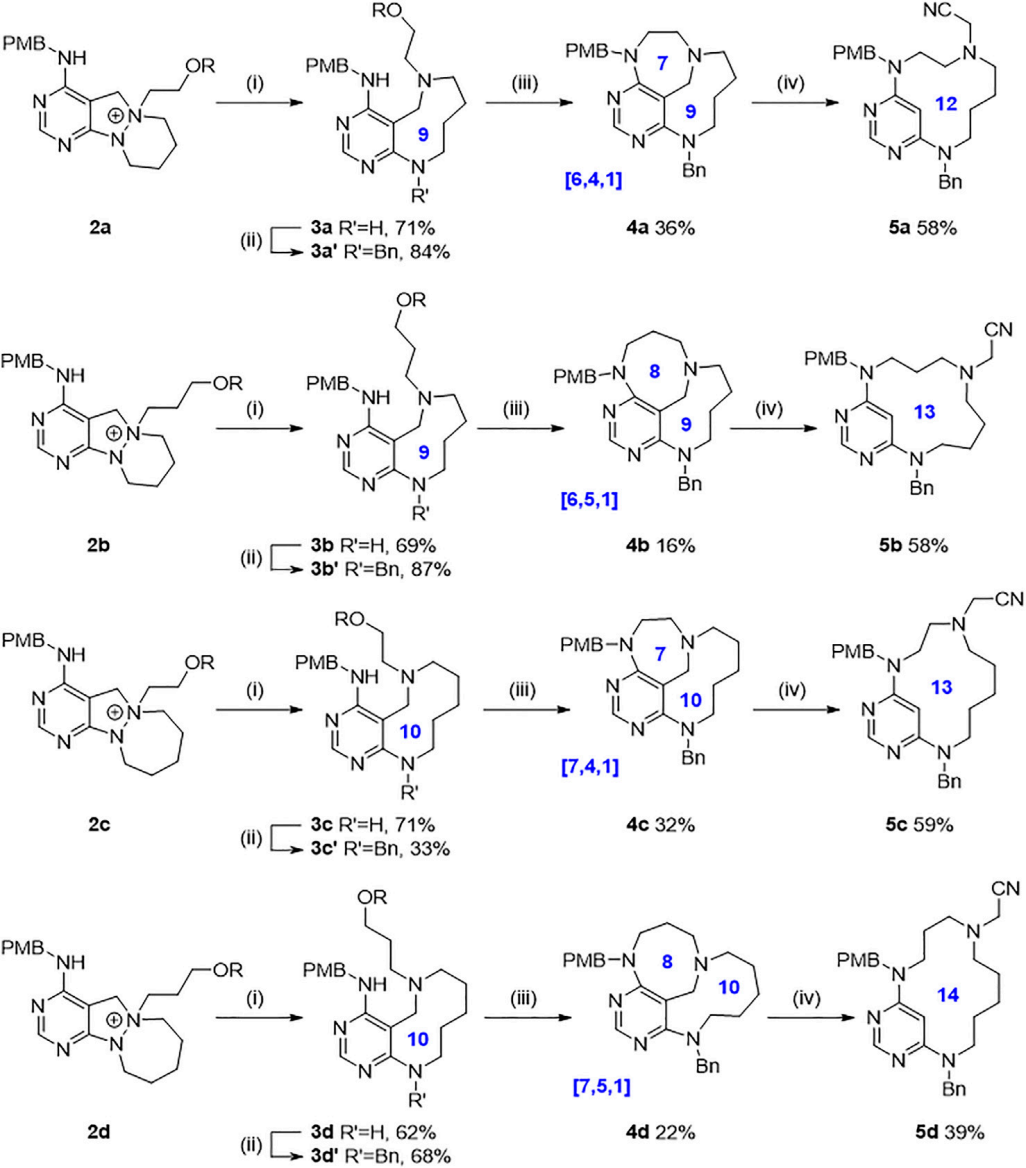
Exploration for each scaffold. Reagents and conditions: (i) NaOEt, NaBH_4_, EtOH, 60°C; (ii) BnBr, ACN, r.t.; (iii) HF/pyridine/THF, r.t., then MsCl, TEA, DCM, r.t., then NaH, dry DMF, r.t.; (iv) AuCl, TMSCN, DCE, 80 C. R = *tert*-butyldiphenylsilyl.

With key intermediates **2a–2d** varying in the ring size of cyclic hydrazines and the chain length of alkylating partners in hand, we explored the synthetic feasibility of each designed scaffold (Scheme 1, see the Supplementary Material for detailed procedures). First of all, the central N–N bond cleavage reaction of *N-*quaternized key intermediates **2a–2d** under sodium ethoxide and sodium borohydride in elevated temperature condition proceeded robustly to afford conformationally labile medium cycles (**3a–3d)** containing pyrimidine-embedded 9-membered diazonanes (**3a** and **3b**) or 10-membered diazecanes (**3c** and **3d**) in a single step with moderate to good yields. Furthermore, introducing a benzyl group as an *R* ^2^ substituent occurred selectively at the newly generated aniline moiety to yield **3a′–3d′** with generally good yields. We then pursued converting the silyl-protected hydroxyl group to a leaving group and the subsequent intramolecular cyclization reaction to afford conformationally rigid methano-bridged cycles (**4**). Both the ring size of medium cycles and the length of appending alkyl chains gave rise to the structural diversity of bridged cyclic skeletons with unique ring-size combinations (**4a** [6.4.1], **4b** [6.5.1], **4c** [7.4.1], and **4d** [7.5.1]). Finally, we applied an unconventional gold-catalyzed C–C bond cleavage reaction to access relatively flexible macrocycles (Koo et al., 2017). In this case, the methano-bridge of the bridged scaffold was the appropriate bond cleavage site by the gold-mediated activation of the diaminopyrimidine ring. The iminium intermediate produced by gold-catalyzed ring opening reaction was trapped by an external cyanide nucleophile to append the R^3^ substituent, thus readily generating 12- to 14-membered macrocyclic scaffolds in a single step. As we installed the functional handles at R^1^, R^2^, and R^3^ positions of each scaffold, we could secure further appendage diversity through introducing various moieties instead of *p*-methoxybenzyl (R^1^) and benzyl (R^2^) groups or modifying the cyanide (R^3^) group to other moieties. Collectively, we obtained ten distinct medium/macro- and bridged cyclic scaffolds sharing pyrimidine moiety with skeletal and conformational flexibility.

### Chemoinformatic Analysis

**FIGURE 4:**
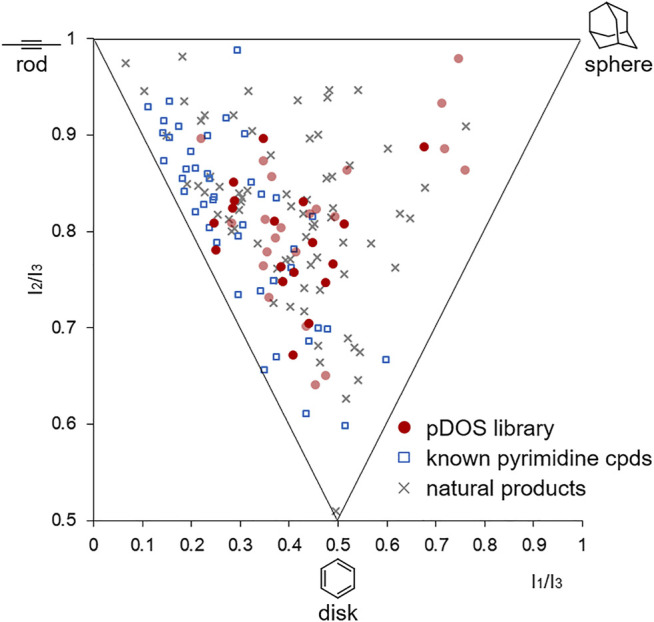
Principal moment of inertia (PMI) plot of natural products, bioactive pyrimidine compounds, and all new molecules synthesized in this study. The 3D molecular shapes of the selected conformers of the newly synthesized compounds (red circles, The dark red circles represent the lowest energy conformers) were compared with reference sets of 45 known bioactive pyrimidine compounds (blue squares) and 60 diverse natural products (grey × shape).

To examine the overall shape diversity of newly synthesized pyrimidine-embedded medium/macro- and bridged cyclic molecules, we performed principal moment of inertia (PMI) analysis. Among the searched conformers of all synthesized molecules, we selected the conformers of which the Boltzmann population is higher than 10% at room temperature. The resulting selected conformers were compared with a set of 45 known pyrimidine-containing bioactive molecules and a set of 60 diverse natural products (Kopp et al., 2012). As shown in Figure 4, the selected conformers of newly synthesized heterocyclic molecules obtained *via* skeletal transformation covered broader areas with more spherical properties similar to natural products, different from the known pyrimidine-embedded bioactive molecules plotted on rod- and disk-like regions. This high shape diversity of our pyrimidine-embedded medium/macro- and bridged cyclic molecules indicates their potential for distinct biological activities compared to the known flat pyrimidine compounds.

## Conclusion

In conclusion, we constructed a small collection of structurally diverse pyrimidine-embedded medium/macro- and bridged cyclic molecules with unique conformational flexibility. We envisioned that building a set of pyrimidine-containing polyheterocycles with skeletal diversity could be a valuable resource to investigate how differences in skeletal flexibility influence the function of the molecules. Using skeletal transformation strategy, we accessed conformationally flexible 9- to 14-member medium/macrocycles as well as rigid tricycles and bridged cycles sharing pyrimidine moiety. Conformational analysis showed that all synthesized molecules in this study have broad coverage of 3D molecular shapes in PMI, unlike the known bioactive pyrimidine molecules. Therefore, we are confident that this collection of pyrimidine-embedded polyheterocycles would serve as a valuable resource in exploring unique biological activities unattainable with previous pyrimidine compounds.

## Data Availability

The datasets presented in this study can be found in online repositories. The names of the repository/repositories and accession number(s) can be found in the article/Supplementary Material.

## Author’s Note

Korea patent application number [10-2022-0040110 (Mar. 31st, 2022)] and [10-2022-0040133 (Mar. 31st, 2022)].
