# Supplementary material for: Design and Synthesis of Conformationally Diverse Pyrimidine-Embedded Medium/Macro- and Bridged Cycles via Skeletal Transformation
Source: Front Chem. 2022 Apr 4;10:841250. doi: 10.3389/fchem.2022.841250 (PMC9014854; doi:10.3389/fchem.2022.841250)
Supplement: Supplementary file 1 [file DataSheet1.pdf]

Supplementary Material

Design and Synthesis of Conformationally Diverse Pyrimidine-Embedded Medium/Macro- and Bridged Cyclic Small Molecules *via* Skeletal Transformation

Yoona Choi, Subin Lee, Heejun Kim, and Seung Bum Park*

CRI Center for Chemical Proteomics, Department of Chemistry, Seoul National University, Seoul, 08826 Republic of Korea

*** Correspondence:**
Prof. Seung Bum Park
sbpark@snu.ac.kr

1 Supplementary Data

1.1 General information

All commercially available reagents and solvents were purchased from commercial vendors and used without further purification unless noted otherwise. ^{1}H and ^{13}C NMR spectra were obtained on Agilent 400–MR DD2 Magnetic Resonance System (400 MHz, Agilent Technologies), Varian Inova–500 (500 MHz, Varian Associates), Ascend 500 (500 MHz, Bruker), or AVANCE 600 (600 MHz, Bruker). Chemical shifts were measured in parts per million (δ), referenced to tetramethylsilane (TMS) as the internal standard or to the residual solvent peak ($CDCl_3$, ^{1}H: 7.27, ^{13}C: 77.00; MeOH-d_4, ^{1}H: 3.31, ^{13}C: 49.15; acetone-d_6, ^{1}H: 2.05, ^{13}C: 29.92; DMSO-d_6, ^{1}H: 2.50, ^{13}C: 39.50; DCM-d_2, ^{1}H: 5.32, ^{13}C: 54.00). Multiplicities were indicated as follows: s (singlet), d (doublet), t (triplet), q (quartet), quint (quintet), m (multiplet), dd (doublet of doublets), dt (doublet of triplets), brs (broad singlet), and so on. Coupling constants were reported in hertz (Hz). Low-resolution mass spectra (LRMS) were analyzed on LCMS-2020 (Shimazu) using the electrospray ionization (ESI) method. High-resolution mass spectra (HRMS) were analyzed on Compact (Bruker) or Orbitrap Explorise 120 (ThermoFisher Scientific) using the electrospray ionization (ESI) method. The conversion of starting materials was monitored using thin-layer chromatography (TLC) (Merck Kiselgel 60 F_{254} plates), and components were visualized by observation under UV light (254 and 365 nm) or by treating the TLC plates with visualizing agents such as $KMnO_4$, phosphomolybdic acid, and ninhydrin followed by thermal visualization. Products were purified by flash column chromatography on Merck Kieselgel 60 (230–400 mesh). Conformational analysis was performed in CONFLEX 7 (MMFF94s force field) and visualized by Discovery Studio (Accelrys). The moment of inertia values of synthesized core skeletons was calculated by PreADMET software to plot the principal moment of inertia (PMI).

1.2 Synthetic procedure and compound characterization

1.2.1. General synthetic procedure for the preparation of 1a–1b

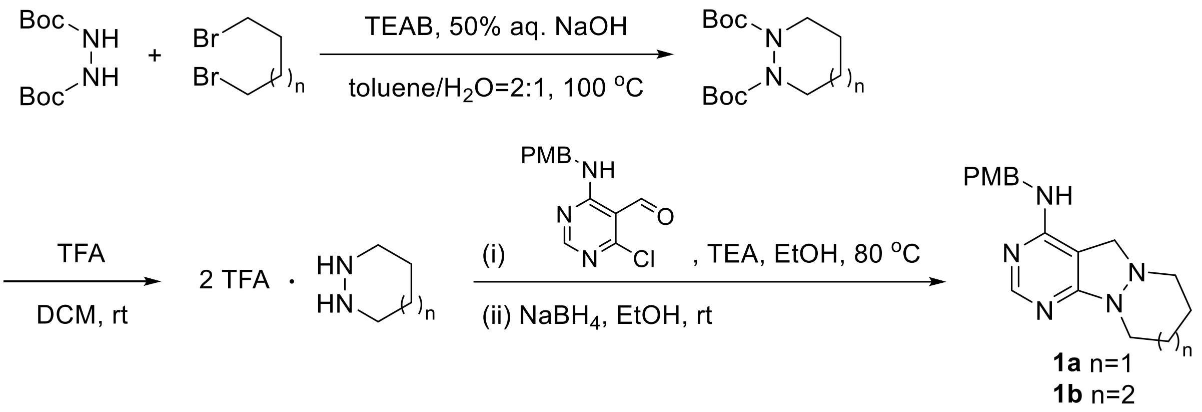

Scheme S1. Synthetic scheme for starting tricycles **1a**–**1b**.

To a solution of di-*tert*-butyl hydrazodicarboxylate in toluene/50% aq. NaOH=2:1 solution (0.1 M), tetraethylammonium bromide (TEAB, 0.15 equiv.) and alkyl dibromide (1.5 equiv.) were added at room temperature. The resulting mixture was vigorously stirred at 100 °C. After the completion of the reaction was checked by TLC, the resulting solution was quenched with deionized water and saturated aq. NaCl, and the organic material was extracted twice with ethyl acetate (EA). The combined organic extracts were dried over anhydrous Na_2SO_4(s) and filtered. After the solvent was evaporated under reduced pressure, the residue was purified by silica-gel flash column chromatography to obtain the desired Boc-protected cyclic hydrazines [82% (n=1) and 83% (n=2)]. The resulting Boc-protected cyclic hydrazines were dissolved in dichloromethane (DCM) and mixed with trifluoroacetic acid (TFA). The reaction mixture was stirred at room temperature. After the completion of the reaction was checked by TLC, the resulting solution was concentrated under reduced pressure to obtain the desired cyclic hydrazines (quantitative yields).

To a ethanol solution (0.1 M) of 4-chloro-6-(4-methoxybenzylamino)pyrimidine-5-carboxaldehyde that was prepared by using the reported synthetic procedure (Choi et al., 2015), cyclic hydrazines (1.1 equiv.) and triethylamine (TEA, 5 equiv.) were added at room temperature. The resulting mixture was stirred at 80 °C. After the complete consumption of pyrimidine was checked by TLC, the reaction mixture was cooled down to room temperature. Then, $NaBH_4$ (3 equiv.) was added to the reaction mixture, and the resulting mixture was stirred at room temperature. When the completion of the reaction was checked by TLC, the resulting mixture was concentrated under reduced pressure, quenched with deionized water and saturated aq. NaCl, and the organic material was extracted three times with EA. The combined organic extracts were dried over anhydrous Na_2SO_4(s) and filtered. After the solvent was evaporated under reduced pressure, the residue was purified by silica-gel flash column chromatography to obtain the desired compounds **1a**–**1b**.

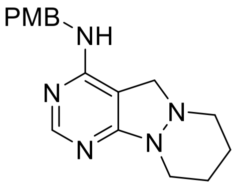

(**1a**) Yield: 81%; ^{1}H NMR (400 MHz, $CDCl_3$) δ 8.18 (s, 1 H), 7.24 (d, *J* = 8.2 Hz, 2 H), 6.87 (d, *J* = 8.2 Hz, 2 H), 4.54 (s, 3 H), 3.90 (brs, 3 H), 3.80 (s, 3 H), 2.81 (brs, 2 H), 1.76 (quint, *J* = 5.6 Hz, 2 H), 1.62 (brs, 2 H); ^{13}C NMR (100 MHz, $CDCl_3$) δ 165.3, 158.9, 157.8, 156.4, 131.0, 128.6, 114.1, 93.3, 55.2, 54.5, 54.1, 45.1, 44.7, 24.5, 23.2; HRMS (ESI) *m/z* calcd for $C_{17}H_{22}N_5O$ $[M+H]^+$: 312.1819; Found: 312.1819.

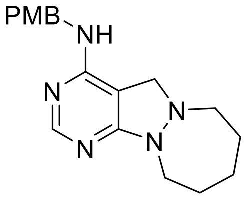

(**1b**) Yield: 68%; ^{1}H NMR (500 MHz, $CDCl_3$) δ 8.21 (s, 1 H), 7.24 (d, *J* = 8.4 Hz, 2 H), 6.87 (m, *J* = 8.8 Hz, 2 H), 4.56 (d, *J* = 5.4 Hz, 2 H), 4.51 (brs, 1 H), 4.11 (s, 2 H), 3.79 (s, 3 H), 3.52 (t, *J* = 5.9 Hz, 2 H), 2.89 (t, *J* = 5.9 Hz, 2 H), 1.83 (quint, *J* = 4.9 Hz, 2 H), 1.742-1.737 (m, 4 H); ^{13}C NMR (100 MHz, $CDCl_3$) δ 166.9, 159.0, 158.2, 156.5, 130.9, 128.8, 114.0, 94.0, 62.0, 56.3, 55.3, 50.4, 44.6, 29.0, 27.5, 25.0; HRMS (ESI) *m/z* calcd for $C_{18}H_{24}N_5O$ $[M+H]^+$: 326.1976; Found: 326.1975.

1.2.2. General synthetic procedure for the preparation of 2a–2d

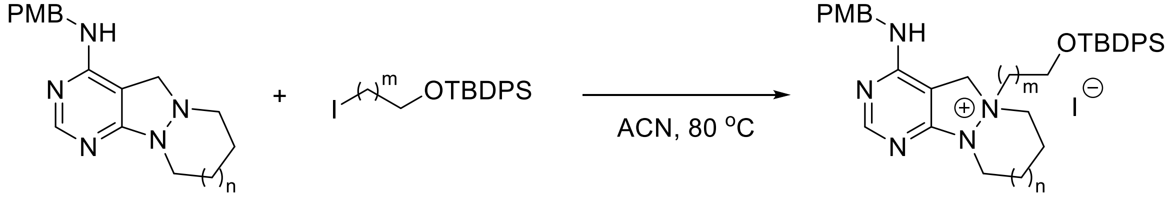

1a n=1
1b n=2

2a n=1, m=1
2b n=1, m=2
2c n=2, m=1
2d n=2, m=2

Scheme S2. Synthetic scheme for key intermediates **2a**–**2d**.

To a solution of **1a**–**1b** in acetonitrile (ACN, 0.2 M), alkylating reagent (3 equiv.), prepared by using the reported synthetic procedure (Fürstner et al., 2009), was added at room temperature, and the resulting mixture was stirred at 80 °C. After the complete consumption of starting material was checked by TLC, the reaction mixture was concentrated under reduced pressure. The residue was purified by silica-gel flash column chromatography to obtain desired compounds **2a**–**2d**.

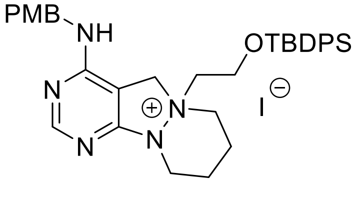

(**2a**) Yield: 72% from **1a**; ^{1}H NMR (500 MHz, MeOH-d_4) δ 8.34 (s, 1 H), 7.63 (d, *J* = 6.4 Hz, 2 H), 7.53 (d, *J* = 6.8 Hz, 2 H), 7.38–7.46 (m, 4 H), 7.33 (t, *J* = 7.35 Hz, 2 H), 7.28 (d, *J* = 8.3 Hz, 2 H), 6.83 (d, *J* = 8.8 Hz, 2 H), 5.70 (d, *J* = 13.2 Hz, 1 H), 5.01 (d, *J* = 13.2 Hz, 1 H), 4.70 (d, *J* = 14.7 Hz, 1 H), 4.52 (d, *J* = 15.2 Hz, 1 H), 4.33–4.42 (m, 2 H), 4.18–4.27 (m, 2 H), 4.08 (d, *J* = 13.2 Hz, 1 H), 3.83–3.94 (m, 2 H), 3.74 (s, 3

H), 3.47–3.53 (m, 1 H), 2.29–2.37 (m, 1 H), 1.88 (d, *J* = 15.2 Hz, 1 H), 1.61–1.64 (m, 2 H), 0.95 (s, 9 H); ^{13}C NMR (100 MHz, DMSO-d_6) δ 162.9, 160.5, 158.6, 136.8, 136.7, 133.0, 132.9, 132.3, 131.5, 130.3, 129.30, 129.26, 115.0, 90.4, 68.8, 63.2, 61.4, 60.0, 55.91, 55.87, 44.8, 42.8, 29.7, 27.3, 19.9, 19.8; HRMS (ESI) *m/z* calcd for $C_{35}H_{44}N_5O_2Si^+$ $[M]^+$: 594.3259; Found: 594.3259.

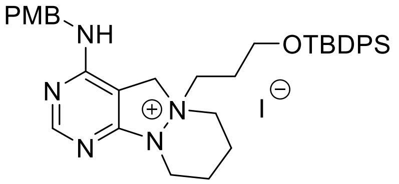

(**2b**) Yield: 72% from **1a**; ^{1}H NMR (400 MHz, MeOH-d_4) δ 8.29 (s, 1 H), 7.68 (d, *J* = 7.4 Hz, 4 H), 7.39–7.44 (m, 6 H), 7.30 (d, *J* = 8.6 Hz, 2 H), 6.86 (d, *J* = 8.6 Hz, 2 H), 4.88 (ABq, ⊿δ_{AB} = 0.04, J_{AB} = 13.7 Hz, 2 H), 4.61 (s, 2 H), 4.15–4.24 (m, 2 H), 4.04–4.11 (m, 1 H), 3.90 (t, *J* = 5.3 Hz, 2 H), 3.81 (dd, *J* = 13.3, 2.8 Hz, 1 H), 3.74 (s, 3 H), 3.68–3.72 (m, 1 H), 3.46–3.54 (m, 1 H), 2.18–2.27 (m, 1 H), 1.96–2.14 (m, 2 H), 1.86 (d, *J* = 15.3 Hz, 1 H), 1.60–1.64 (m, 2 H), 1.07 (s, 9 H); ^{13}C NMR (100 MHz, DMSO-d_6) δ 163.0, 160.6, 160.5, 158.7, 136.8, 134.42, 134.36, 132.2, 131.3, 130.3, 129.2, 115.1, 90.4, 60.3, 61.6, 60.6, 58.0, 55.92, 55.89, 45.0, 42.5, 27.8, 27.6, 20.1, 19.7; HRMS (ESI) *m/z* calcd for $C_{36}H_{46}N_5O_2Si^+$ $[M]^+$: 608.3415; Found: 608.3415.

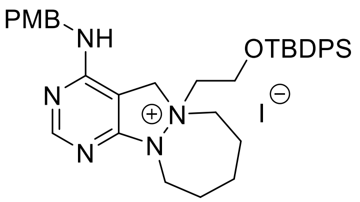

(**2c**) Yield: 81% from **1b**; ^{1}H NMR (400 MHz, MeOH-d_4) δ 8.33 (s, 1 H), 7.58 (d, *J* = 6.8 Hz, 2 H), 7.35–7.44 (m, 8 H), 7.25 (d, *J* = 8.4 Hz, 2 H), 6.78 (d, *J* = 8.8 Hz, 2 H), 5.79 (d, *J* = 14.4 Hz, 1 H), 5.22 (d, *J* = 14.4 Hz, 1 H), 4.56 (ABq, $\Delta\delta_{AB}$ = 0.05, J_{AB} = 14.6 Hz, 2 H), 4.31–4.48 (m, 2 H), 4.22 (d, *J* = 16.8 Hz, 1 H), 3-98–4.06 (m, 3 H), 3.85 (dd, *J* = 13.4 Hz, 3.0 Hz, 1 H), 3.76 (d, *J* = 11.2 Hz, 1 H), 3.72 (s, 3 H), 2.07 (t, *J* = 5.6 Hz, 1 H), 1.73–1.85 (m, 3 H), 1.59 (t, *J* = 9.2 Hz, 2 H), 0.92 (s, 9 H); ^{13}C NMR (100 MHz, MeOH-d_4) δ 162.7, 160.6, 160.5, 157.4, 136.8, 136.5, 133.1, 132.8, 132.1, 131.5, 131.4, 130.6, 129.32, 129.30, 115.1, 89.2, 73.5, 71.1, 69.8, 59.8, 55.9, 45.14, 45.07, 28.1, 27.4, 26.5, 23.3, 19.8; HRMS (ESI) m/z calcd for $C_{36}H_{46}N_5O_2Si^+$ $[M]^+$: 608.3415; Found: 608.3415.

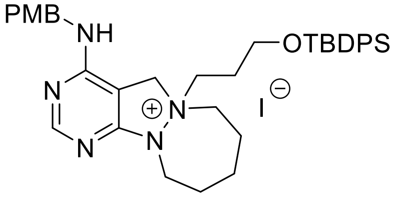

(**2d**) Yield: 68% from **1b**; ^{1}H NMR (500 MHz, MeOH-d_4) δ 8.25 (s, 1 H), 7.61–7.34 (m, 4 H), 7.37–7.42 (m, 6 H), 7.30 (d, *J* = 8.0 Hz, 2 H), 6.85 (d, *J* = 9.0 Hz, 2 H), 5.14 (ABq, ⊿δ_{AB} = 0.03, J_{AB} = 14.8 Hz, 2 H), 4.59 (ABq, ⊿δ_{AB} = 0.02, J_{AB} = 14.5 Hz, 2 H), 4.29–4.37 (m, 1 H), 4.22 (d, *J* = 16.5 Hz, 1 H), 4.04 (dd, *J* = 14.5 Hz, 9.5 Hz, 1 H), 3.91–3.97 (m, 1 H), 3.83–3.88 (m, 1 H), 3.74–3.81 (m, 3 H), 3.71 (s, 3 H), 2.03–2.08 (m, 1 H), 1.71–1.88 (m, 5 H), 1.54–1.66 (m, 2 H), 1.03 (s, 9 H); ^{13}C NMR (125 MHz, MeOH-d_4) δ 162.3, 160.6, 160.5, 157.4, 136.8, 136.7, 134.4, 134.3, 132.3, 131.2, 130.3, 129.1, 115.1, 88.2, 71.5, 67.7, 61.6, 55.9, 45.0, 28.0, 27.6, 27.2, 27.0, 23.7, 20.1; HRMS (ESI) m/z calcd for $C_{37}H_{48}N_5O_2Si^+$ $[M]^+$: 622.3572; Found: 622.3572.

1.2.3. General synthetic procedure for the preparation of 3a–3d and **3a′–3d′**

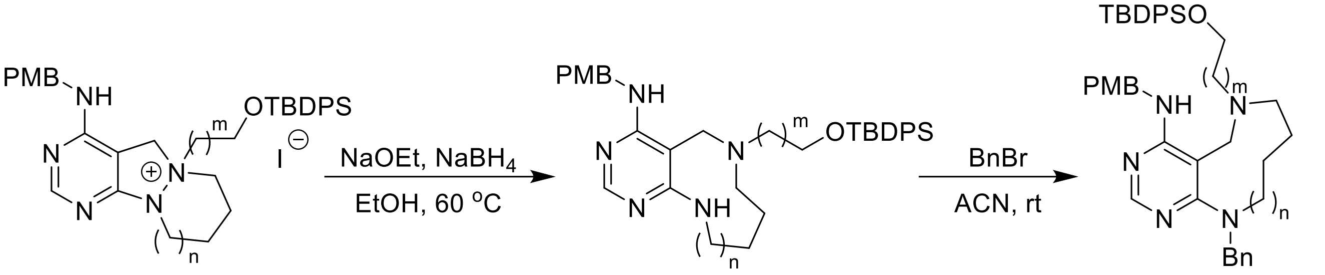

2a n=1, m=1
2b n=1, m=2
2c n=2, m=1
2d n=2, m=2

3a n=1, m=1
3b n=1, m=2
3c n=2, m=1
3d n=2, m=2

3a' n=1, m=1
3b' n=1, m=2
3c' n=2, m=1
3d' n=2, m=2

Scheme S3. Synthetic scheme for medium-sized rings **3a–3d** and **3a′–3d′**.

To a solution of **2a**–**2d** in ethanol (0.05 M), NaOEt (1.5 equiv.) and $NaBH_4$ (20 equiv.) were added at room temperature. The resulting mixture was stirred at 60 °C. After the completion of the reaction was checked by TLC, the reaction mixture was concentrated under reduced pressure, quenched with deionized water and saturated aq. $NaHCO_3$, and the organic material was extracted three times with ethyl acetate (EA). The combined organic extracts were dried over anhydrous Na_2SO_4(s) and filtered. After the solvent was evaporated under reduced pressure, the residue was purified by silica-gel flash column chromatography to obtain desired compounds **3a**–**3d**.

To a solution of **3a**–**3d** in ACN (0.05 M), benzyl bromide (1.10 equiv.) was added at room temperature with stirring. When the completion of the reaction was checked by TLC, the resulting mixture was concentrated under reduced pressure, quenched with deionized water and saturated aq. $NaHCO_3$, and the organic material was extracted three times with EA and three times with DCM. The combined organic extracts were dried over anhydrous Na_2SO_4(s) and filtered. The solvent was evaporated under reduced pressure, and the residue was purified by silica-gel flash column chromatography to obtain the desired compounds **3a′**–**3d′**.

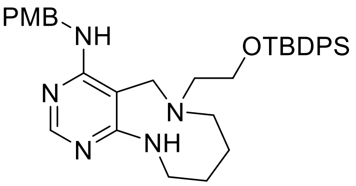

(**3a**) Yield: 71% from **2a**; ^{1}H NMR (400 MHz, $CDCl_3$) δ 8.31 (s, 1 H), 8.25 (brs, 1 H), 7.65–7.67 (m, 4 H), 7.34–7.42 (m, 6 H), 7.25 (d, J = 8.6 Hz, 2 H), 6.87 (d, J = 8.2 Hz, 2 H), 4.57 (d, J = 5.1 Hz, 2 H), 4.36 (brs, 1 H), 3.77–3.79 (m, 5 H), 3.41 (brs, 4 H), 2.75 (t, J = 5.5 Hz, 2 H), 2.34 (brs, 2 H), 1.60–1.61 (m, 2 H), 1.53 (brs, 2 H), 1.04 (s, 9 H); ^{13}C NMR (100 MHz, $CDCl_3$) δ 164.3, 160.3, 159.0, 156.4, 135.6, 133.3, 131.2, 129.7, 129.2, 127.7, 114.0, 95.2, 61.7, 58.9, 55.3, 54.6, 51.1, 45.3, 44.9, 31.4, 28.8, 26.8, 19.1; HRMS (ESI) m/z calcd for $C_{35}H_{46}N_5O_2Si$ $[M+H]^+$: 596.3415; Found: 596.3413.

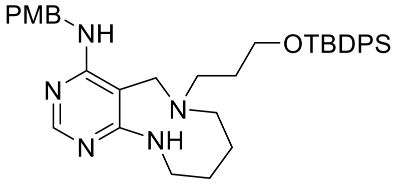

(**3b**) Yield: 69% from **2b**; ^{1}H NMR (400 MHz, $CDCl_3$) δ 8.30 (s, 1 H), 8.16 (brs, 1 H), 7.66 (d, *J* = 6.3 Hz, 4 H), 7.35–7.43 (m, 6 H), 7.27 (d, *J* = 8.2 Hz, 2 H), 6.88 (d, *J* = 8.2 Hz, 2 H), 4.58 (d, *J* = 4.7 Hz, 2 H), 4.47 (brs, 1 H), 3.79 (s, 3 H), 3.74 (t, *J* = 6.1 Hz, 2 H), 3.35 (brs, 4 H), 2.72 (t, *J* = 7.2 Hz, 2 H), 2.31 (brs, 2 H), 1.76 (quint, *J* = 6.6 Hz, 2 H), 1.58 (brs, 2 H), 1.52 (brs, 2 H), 1.05 (s, 9 H); ^{13}C NMR (100 MHz, $CDCl_3$) δ 164.3, 160.4, 159.0, 156.4, 135.5, 133.7, 131.2, 129.6, 129.2, 127.6, 114.0, 95.2, 61.7, 55.3, 54.7, 54.2, 51.1, 45.4, 44.9, 31.3, 30.8, 29.0, 26.8, 19.2; HRMS (ESI) *m/z* calcd for $C_{36}H_{48}N_5O_2Si$ $[M+H]^+$: 610.3572; Found: 610.3569.

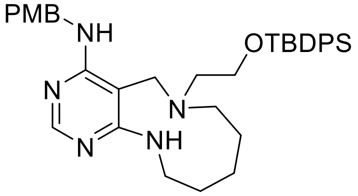

(**3c**) Yield: 71% from **2c**; ^{1}H NMR (400 MHz, $CDCl_3$) δ 8.67 (brs, 1 H), 8.25 (s, 1 H), 7.66 (d, *J* = 6.4 Hz, 4 H), 7.32–7.41 (m, 6 H), 7.25 (d, *J* = 8.4 Hz, 2 H), 6.84 (d, *J* = 8.8 Hz, 2 H), 4.56 (d, *J* = 4.8 Hz, 2 H), 4.10 (t, *J* = 3.6 Hz, 1 H), 3.80 (s, 5 H), 3.31 (brs ,2 H), 2.67 (brs, 4 H), 2.33 (brs, 2 H), 1.60 (brs, 6 H), 1.04 (s, 9 H); ^{13}C NMR (125 MHz, $CDCl_3$) δ 162.9, 159.7, 159.1, 156.4, 135.7, 133.5, 131.7, 129.9, 129.4, 127.9, 114.2, 92.0, 61.5, 55.4, 54.7, 50.9, 48.1, 45.2, 43.8, 27.4, 27.0, 26.5, 22.0, 19.2; HRMS (ESI) *m/z* calcd for $C_{36}H_{48}N_5O_2Si$ $[M+H]^+$: 610.3572; Found: 610.3572.

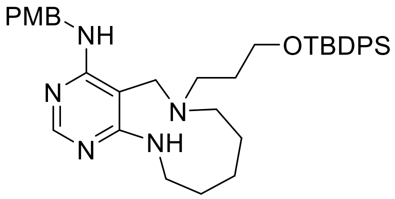

(**3d**) Yield: 62% from **2d**; ^{1}H NMR (400 MHz, $CDCl_3$) δ 8.66 (brs, 1 H), 8.26 (s, 1 H), 7.65 (d, *J* = 6.4 Hz, 4 H), 7.34–7.44 (m, 6 H), 7.27 (d, *J* = 8.4 Hz, 2 H), 6.88 (d, *J* = 8.8 Hz, 2 H), 4.58 (d, *J* = 4.4 Hz, 2 H), 4.21 (t, *J* = 5.2 Hz, 1 H), 3.80 (s, 3 H), 3.76 (t, *J* = 6.0 Hz, 2 H), 3.22 (brs, 8 H), 1.77 (quint, *J* = 6.6 Hz, 2 H), 1.54 (brs, 6 H), 1.05 (s, 9 H); ^{13}C NMR (100 MHz, $CDCl_3$) δ 163.0, 159.7, 159.1, 156.4, 135.7, 133.9, 131.7, 129.8, 129.4, 127.8, 114.2, 92.1, 62.0, 55.4, 50.3, 49.6, 48.5, 45.2, 43.9, 30.0, 27.4, 27.0, 26.5, 22.3, 19.3; HRMS (ESI) *m/z* calcd for $C_{37}H_{50}N_5O_2Si$ $[M+H]^+$: 624.3729; Found: 624.3728.

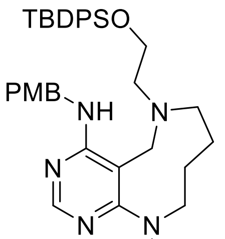

(**3a′**) Yield: 84% from **3a**; ^{1}H NMR (400 MHz, $CDCl_3$) δ 8.26 (s, 1 H), 7.62 (d, *J* = 6.8 Hz, 4 H), 7.30–7.43 (m, 11 H), 7.22 (brs, 1 H), 7.16 (d, *J* = 8.8 Hz, 2 H), 6.78 (d, *J* = 8.4 Hz, 2 H), 4.72 (s, 2 H), 4.54 (d, *J* = 5.6 Hz, 2 H), 3.75 (s, 3 H), 3.70–3.73 (m, 4 H), 3.47 (brs, 2 H), 2.69–2.72 (m, 4 H), 1.48 (brs, 4 H), 1.03 (s, 9 H); ^{13}C NMR (100 MHz, $CDCl_3$) δ 164.1, 162.9, 158.6, 156.1, 140.2, 135.6, 133.7, 132.1, 129.8, 128.43, 128.40, 128.2, 127.8, 126.9, 114.0, 97.1, 62.7, 56.8, 56.0, 55.3, 53.6, 52.7, 50.2, 44.3, 29.2, 27.0, 24.1, 19.3; HRMS (ESI) *m/z* calcd for $C_{42}H_{52}N_5O_2Si$ $[M+H]^+$: 686.3885; Found: 686.3884.

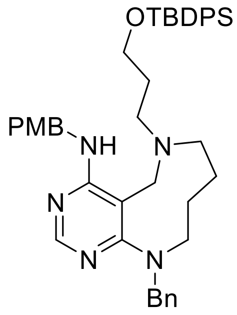

(**3b′**) Yield: 87% from **3b**; ^{1}H NMR (400 MHz, $CDCl_3$) δ 8.26 (s, 1 H), 7.63 (d, *J* = 6.4 Hz, 4 H), 7.30–7.43 (m, 11 H), 7.22 (t, *J* = 4.0 Hz, 1 H), 7.16 (d, *J* = 8.4 Hz, 2 H), 6.77 (d, *J* = 8.8 Hz, 2 H), 4.74 (s, 2 H), 4.51 (d, *J* = 5.6 Hz, 2 H), 3.71 (s, 3 H), 3.58–3.60 (m, 4 H), 3.46 (brs, 2 H), 2.67 (brs, 2 H), 2.61(t, *J* = 7.0 Hz, 2 H), 1.641 (quint, *J* = 6.4 Hz, 2 H), 1.48 (brs, 4 H), 1.03 (s, 9 H); ^{13}C NMR (100 MHz, $CDCl_3$) δ 164.1, 162.5, 158.7, 156.1, 140.1, 135.7, 134.0, 131.9, 129.8, 128.6, 128.4, 128.2, 127.8, 126.9, 114.0, 96.6, 62.0, 61.4, 55.3, 53.3, 52.1, 51.6, 44.4, 31.0, 29.3, 27.0, 23.8, 19.3; HRMS (ESI) *m/z* calcd for $C_{43}H_{54}N_5O_2Si$ $[M+H]^+$: 700.4042; Found: 700.4041.

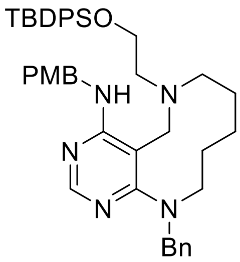

(**3c′**) Yield: 33% from **3c**; ^{1}H NMR (400 MHz, $CDCl_3$) δ 8.50 (s, 1 H), 7.62 (d, *J* = 7.2 Hz, 4 H), 7.20–7.44 (m, 12 H), 7.13 (d, *J* = 8.0 Hz, 2 H), 6.76 (d, *J* = 8.4 Hz, 2 H), 4.52 (d, *J* = 5.2 Hz, 2 H), 4.02 (s, 2 H), 3.77–3.85 (m, 2 H), 3.74 (s, 3 H), 3.71 (t, J = 6.0 Hz, 2 H), 2.97 (brs, 2 H), 2.74 (t, *J* = 5.8 Hz, 2 H), 2.44 (t, *J* = 6.0 Hz, 2 H), 1.58–1.61 (m, 2 H), 1.50 (brs, 2 H), 1.18–1.19 (m, 2 H), 1.02 (s, 9 H); ^{13}C NMR (100 MHz, $CDCl_3$) δ 166.4, 163.9, 158.7, 157.7, 139.0, 135.6, 133.8, 131.4, 129.8, 129.3, 128.4, 128.2, 127.8, 127.1, 114.0, 111.3, 63.0, 60.1, 55.3, 55.1, 53.7, 49.1, 45.4, 44.1, 27.0, 24.6, 22.7, 20.6, 19.3; HRMS (ESI) *m/z* calcd for $C_{43}H_{54}N_5O_2Si$ $[M+H]^+$: 700.4042; Found: 700.4041.

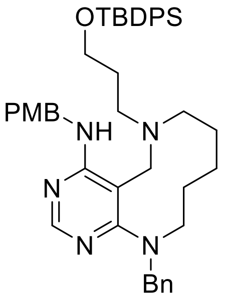

(**3d′**) Yield: 68% from **3d**; ^{1}H NMR (400 MHz, $CDCl_3$) δ 8.51 (s, 1 H), 7.64 (d, *J* = 6.4 Hz, 4 H), 7.21–7.44 (m, 12 H), 7.12 (d, *J* = 8.4 Hz, 2 H), 6.77 (d, *J* = 8.8 Hz, 2 H), 4.48 (d, *J* = 5.6 Hz, 2 H), 4.03 (s, 2 H), 3.72 (s, 3 H), 3.61 (t, *J* = 6.0 Hz, 2 H), 2.96 (brs, 2 H), 2.66 (t, *J* = 7.2 Hz, 2 H), 2.41 (t, *J* = 5.6 Hz, 2 H), 1.59–1.68 (m, 4 H), 1.51 (brs, 2 H), 1.20 (brs, 2 H), 1.04 (s, 9 H); ^{13}C NMR (125 MHz, $CDCl_3$) δ 166.3, 163.9, 158.8, 157.7, 139.1, 135.7, 134.0, 131.3, 129.8, 129.3, 128.6, 128.3, 127.8, 127.1, 114.0, 111.4, 62.2, 60.1, 55.4, 53.7, 49.7, 48.9, 44.4, 44.2, 31.0, 27.0, 24.6, 22.7, 20.4, 19.3; HRMS (ESI) *m/z* calcd for $C_{44}H_{56}N_5O_2Si$ $[M+H]^+$: 714.4198; Found: 714.4197.

1.2.4. General synthetic procedure for the preparation of 4a–4d

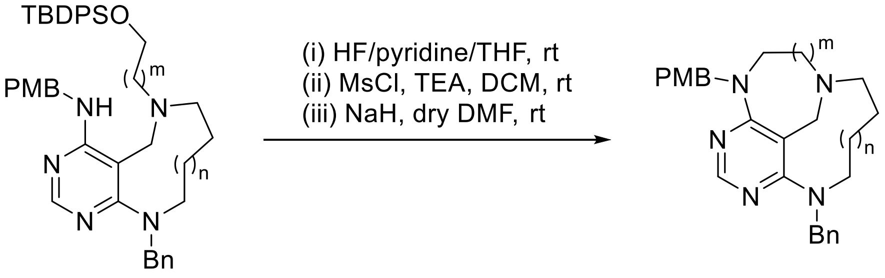

3a' n=1, m=1
3b' n=1, m=2
3c' n=2, m=1
3d' n=2, m=2

4a n=1, m=1
4b n=1, m=2
4c n=2, m=1
4d n=2, m=2

Scheme S4. Synthetic scheme for bridged cycles **4a–4d**.

3a′–3d′ were treated with HF/pyridine/tetrahydrofuran (5/5/90) solution (0.1 M), and the reaction mixture was stirred at room temperature. When the completion of the reaction was checked by TLC, ethoxytrimethylsilane was added and the reaction mixture was allowed to quench any excess HF. The resulting mixture was concentrated under reduced pressure. To a solution of the resulting residue in DCM (0.05 M) and TEA (2.0 equiv.), methanesulfonyl chloride (1.5 equiv.) were added at room temperature with stirring. When the completion of the reaction was checked by TLC, the resulting mixture was concentrated under reduced pressure. The residue and NaH (2.0 equiv.) were dissolved in dry DMF (0.05 M) under an argon atmosphere with stirring at room temperature. After the reaction completion monitored by TLC, the resulting mixture was quenched with deionized water and saturated aq. $NaHCO_3$, and the organic material was extracted three times with EA and three times with DCM. The combined organic extracts were dried over anhydrous Na_2SO_4(s) and filtered. The solvent was evaporated under reduced pressure, and the residue was purified by silica-gel flash column chromatography to obtain desired compounds **4a**–**4d**.

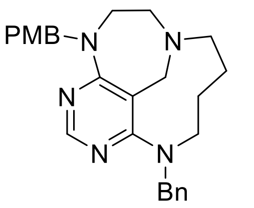

(**4a**) Yield: 36% from **3a′**; ^{1}H NMR (500 MHz, $CDCl_3$) δ 8.24 (s, 1 H), 7.26–7.33 (m, 7 H), 6.87 (d, *J* = 8.8 Hz, 2 H), 5.10 (d, *J* = 14.7 Hz, 1 H), 4.97 (d, *J* = 15.2 Hz, 1 H), 4.42 (d, *J* = 14.7 Hz, 2 H), 3.99 (d, *J* = 14.7 Hz, 1 H), 3.80 (s, 3 H), 3.63 (brs, 2 H), 3.44–3.45 (m, 2 H), 3.10 (dd, *J* = 13.2, 9.3 Hz, 1 H), 2.79–2.85 (m, 2 H), 2.65–2.71(m, 2 H), 1.79–1.83 (m, 1 H), 1.60–1.67 (m, 2 H), 1.44–1.47 (m, 1 H); ^{13}C NMR (100 MHz, $CDCl_3$) δ 167.1, 166.3, 158.7, 154.6, 139.3, 130.9, 129.3, 128.5, 128.3, 127.0, 113.8, 100.5, 55.6, 55.3, 55.2, 54.2, 53.8, 53.6, 53.0, 52.6, 51.7, 28.9, 25.9; HRMS (ESI) *m/z* calcd for $C_{26}H_{32}N_5O$ $[M+H]^+$: 430.2601; Found: 430.2602.

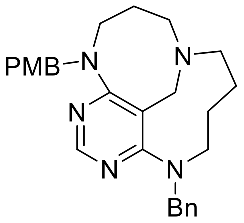

(**4b**) Yield: 16% from **3b′**; ^{1}H NMR (500 MHz, $CDCl_3$) δ 8.26 (s, 1 H), 7.39 (d, *J* = 7.3 Hz, 2 H), 7.26–7.29 (m, 2 H), 7.18–7.21 (m, 3 H), 6.85 (d, *J* = 8.3 Hz, 2 H), 5.01 (d, *J* = 14.7 Hz, 1 H), 4.87 (d, *J* = 15.2 Hz, 1 H), 4.81 (dd, *J* = 13.5, 3.7 Hz, 1 H), 4.75 (d, *J* = 15.2 Hz, 1 H), 4.14 (d, *J* = 14.7 Hz, 1 H), 4.05 (d, *J* = 14.7 Hz, 1 H), 3.79 (s, 3 H), 3.49–3.59 (m, 2 H), 3.11 (dd, *J* = 13.9, 2.7 Hz, 1 H), 2.98 (d, *J* = 14.7 Hz, 1 H), 2.88 (dd, *J* = 11.0, 6.1 Hz, 1 H), 2.70–2.75 (m, 2 H), 2.43–2.48 (m, 1 H), 2.21 (t, *J* = 11.2 Hz, 1 H), 1.93–2.04 (m, 1H), 1.70–1.83 (m, 2 H), 1.45–1.50 (m, 1 H), 1.37–1.40 (m, 1 H); ^{13}C NMR (100 MHz, $CDCl_3$) δ 171.1, 161.3, 158.6, 154.9, 140.4, 131.2, 128.8, 128.5, 128.0, 126.5, 113.8, 105.0, 58.9, 57.3, 57.1, 55.3, 55.2, 55.1, 53.4, 44.9, 28.4, 26.6, 21.4; HRMS (ESI) *m/z* calcd for $C_{27}H_{34}N_5O$ $[M+H]^+$: 444.2758; Found: 444.2758.

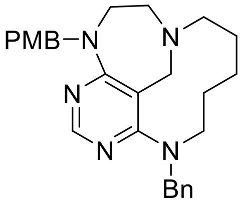

(**4c**) Yield: 35% from **3c′**; ^{1}H NMR (400 MHz, $CDCl_3$) δ 8.19 (s, 1 H), 7.20–7.32 (m, 7 H), 6.78 (d, *J* = 8.4 Hz, 2 H), 5.07 (d, *J* = 15.2 Hz, 1 H), 4.73 (s, 2 H), 4.23 (d, *J* = 14.4 Hz, 1 H), 4.13–4.20 (m, 1 H), 3.99 (d, *J* = 12.8 Hz, 1 H), 3.81 (s, 3 H), 3.25–3.32 (m, 2 H), 3.00 (dd, *J* = 13.6, 10.8 Hz, 1 H), 2.71–2.78 (m, 2 H), 2.67 (d, *J* = 13.2 Hz, 1 H), 2.37 (dt, *J* = 12.4 Hz, 4.1 Hz, 1 H), 2.28 (t, *J* = 11.0 Hz, 1 H), 2.02–2.07 (m, 1 H), 1.81–1.82 (m, 2 H), 1.55–1.61 (m, 2 H), 1.39–1.44 (m, 1 H); ^{13}C NMR (125 MHz, $CDCl_3$) δ 169.1, 166.9, 158.8, 154.2, 139.3, 131.2, 129.8, 128.35, 128.30, 126.9, 113.9, 101.0, 60.1, 57.2, 55.4, 53.7, 53.5, 53.0, 52.6, 51.3, 30.7, 29.8, 24.1; HRMS (ESI) *m/z* calcd for $C_{27}H_{34}N_5O$ $[M+H]^+$: 444.2758; Found: 444.2758.

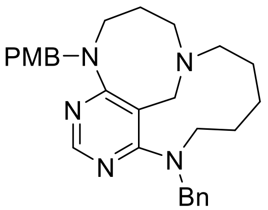

(**4d**) Yield: 22% from **3d′**; ^{1}H NMR (400 MHz, $CDCl_3$) δ 8.42 (s, 1 H), 7.21–7.29 (m, 7 H), 6.86 (d, *J* = 8.8 Hz, 2 H), 4.90 (d, *J* =14.4 Hz, 1 H), 4.49–4.58 (m, 2 H), 4.20–4.33 (m, 3 H), 3.80 (s, 3 H), 3.62–3.70 (m, 1 H), 3.51 (td, *J* = 12.5 Hz, 3.5 Hz, 1 H), 3.04–3.09 (m, 1 H), 2.86–2.93 (m, 1 H), 2.65 (brs, 2 H), 2.39–2.49 (m, 1 H), 2.19–2.26 (m, 1 H), 2.02–2.04 (m, 1 H), 1.94 (brs, 2 H), 1.80–1.86 (m, 2 H), 1.64–1.72 (m, 1 H), 1.50 (brs, 1 H), 1.39–1.43 (m, 1 H); ^{13}C NMR (150 MHz, $CDCl_3$) δ 169.1, 165.8, 158.7, 156.2, 138.0, 130.9, 129.4, 128.7, 128.2, 127.0, 113.8, 106.7, 56.3, 55.2, 54.9, 54.2, 51.0, 50.5, 46.4, 27.4, 26.7, 25.8, 25.1; HRMS (ESI) *m/z* calcd for $C_{28}H_{36}N_5O$ $[M+H]^+$: 458.2915; Found: 458.2914.

1.2.5. General synthetic procedure for the preparation of 5a–5d

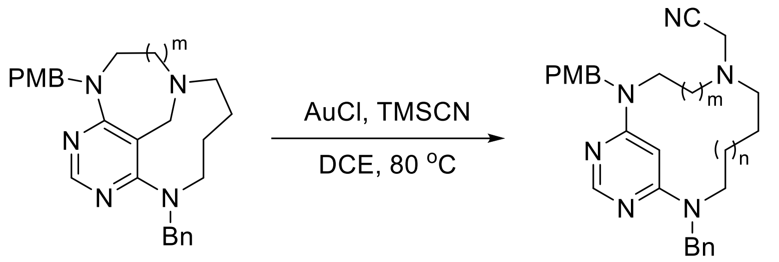

Scheme S5. Synthetic scheme for macrocycles **5a–5d**.

To a solution of **4a**–**4d** in dichloroethane (0.05 M), AuCl (0.5 equiv.) and trimethylsilyl cyanide (3 equiv.) were added at room temperature. The resulting mixture was stirred at 80 °C. After the completion of the reaction (checked by TLC), the reaction mixture was concentrated under reduced pressure. The residue was purified by silica-gel flash column chromatography to obtain desired compounds **5a**–**5d**.

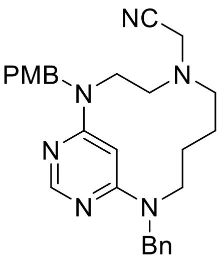

(**5a**) Yield: 66% from **4a**; ^{1}H NMR (400 MHz, $CDCl_3$) δ 8.30 (s, 1 H), 7.23–7.32 (m, 5 H), 7.19 (d, J = 8.4 Hz, 2 H), 6.85 (d, J = 8.4 Hz, 2 H), 5.87 (s, 1 H), 4.86 (d, J = 8.4 Hz, 4 H), 3.80 (s, 3 H), 3.41 (brs, 2 H), 3.39 (s, 2 H), 3.27 (brs, 2 H), 2.74 (brs, 2 H), 2.60 (t, J = 4.4 Hz, 2 H), 1.48 (brs, 4 H); ^{13}C NMR (150 MHz, $CDCl_3$) δ 163.1, 161.5, 159.1, 158.1, 139.4, 131.2, 129.1, 128.6, 128.0, 127.2, 114.5, 114.2, 83.6, 56.2, 55.4, 50.4, 49.9, 48.0, 45.9, 45.7, 43.1, 22.84, 22.79; HRMS (ESI) *m/z* calcd for $C_{27}H_{33}N_6O$ $[M+H]^+$: 457.2711; Found: 457.2710.

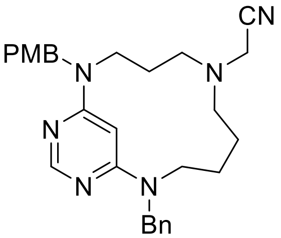

(**5b**) Yield: 58% from **4b**; ^{1}H NMR (400 MHz, $CDCl_3$) δ 8.29 (s, 1 H), 7.24–7.32 (m, 5 H), 7.19 (d, J = 8.8 Hz, 2 H), 6.84 (d, J = 8.4 Hz, 2 H), 6.09 (s, 1 H), 4.87 (brs, 2 H), 4.83 (s, 2 H), 3.79 (s, 3 H), 3.55 (s, 2 H), 3.29 (brs, 2 H), 3.19 (t, J = 8.6 Hz, 2 H), 2.66 (brs, 2 H), 2.44 (brs, 2 H), 1.66 (brs, 4 H), 1.40 (brs, 2 H); ^{13}C NMR (125 MHz, $CDCl_3$) δ 162.6, 162.5, 158.9, 157.8, 139.3, 131.2, 129.2, 128.6, 127.9, 127.2, 114.8, 114.0, 81.6, 55.4, 54.4, 50.5, 50.2, 48.3, 47.3, 43.0, 24.8, 24.7, 22.2; HRMS (ESI) *m/z* calcd for $C_{28}H_{35}N_6O$ $[M+H]^+$: 471.2867; Found: 471.2867.

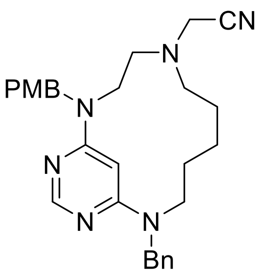

(**5c**) Yield: 59% from **4c**; ^{1}H NMR (400 MHz, $CDCl_3$) 8.32 (s, 1 H), 7.24–7.32 (m, 5 H), 7.19 (d, *J* = 8.8 Hz, 2 H), 6.85 (d, *J* = 8.4 Hz, 2 H), 5.64 (s, 1 H), 4.92 (s, 2 H), 4.85 (s, 2 H), 3.80 (s, 3 H), 3.39 (s, 2 H), 3.38 (t, *J* = 5.6 Hz, 2 H), 3.21 (t, *J* = 7.4 Hz, 2 H), 2.62–2.65 (m, 4 H), 1.47–1.49 (m, 4 H), 1.38 (q, *J* = 6.4 Hz, 2 H); ^{13}C NMR (125 MHz, $CDCl_3$) δ 162.0, 161.8, 159.0, 158.1, 139.2, 131.0, 129.2, 128.6, 127.8, 127.1, 114.8, 114.1, 81.8, 55.4, 53.8, 52.0, 51.0, 50.9, 49.5, 48.8, 40.6, 23.8, 22.3, 21.8; HRMS (ESI) *m/z* calcd for $C_{28}H_{35}N_6O$ $[M+H]^+$: 471.2867; Found: 471.2867.

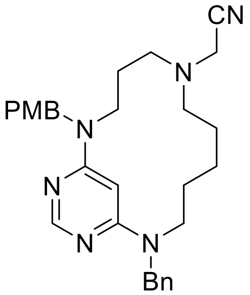

(**5d**) Yield: 39% from **4d**; ^{1}H NMR (400 MHz, $CDCl_3$) δ 8.27 (s, 1 H), 7.24–7.32 (m, 5 H), 7.19 (d, *J* = 8.8 Hz, 2 H), 6.85 (d, *J* = 8.4 Hz, 2 H), 5.48 (s, 1 H), 4.92 (s, 2 H), 4.87 (brs, 2 H), 3.79 (s, 3 H), 3.50 (s, 2H), 3.35 (brs, 2 H), 3.19 (dd, *J* = 10.0 Hz, 6.8 Hz, 2 H), 2.56 (brs, 2 H), 2.50 (brs, 2 H), 1.66 brs, 2 H), 1.58 (brs, 4 H), 1.38 (brs, 2 H); ^{13}C NMR (125 MHz, $CDCl_3$) δ 161.8, 161.6, 158.7, 157.9, 139.1, 131.1, 128.8, 128.4, 127.5, 127.0, 114.5, 113.8, 78.7, 55.3, 53.3, 50.6, 49.2, 47.5, 47.4, 45.0, 42.8, 23.9, 23.0, 21.6, 21.4; HRMS (ESI) *m/z* calcd for $C_{29}H_{37}N_6O$ $[M+H]^+$: 485.3024; Found: 485.3023.

1.3 Conformational analysis

1.3.1 Conformational analysis search method

Force Field	MMFF94s
Solvent Effect	None
Electrostatic Calculation Method	Bond Charge Increment
Optimization Method	Full-Matrix Newton-Raphson
Optimize by	Energy
Search Limit	2.5%
	Corner Flap (O)
	Edge Flip (O)
	Stepwise Rotation (O)

1.3.2 Conformational analysis result

Table S1. Conformational analysis result of medium/macro- and bridged rings in bioactive natural products.

scaffold	potential energy (kcal/mol)	relative energy (kcal/mol)	Boltzmann population (%)
antimycin A_{3b}	-15.88	0	37.0
	-15.74	0.15	29.0
	-15.64	0.24	24.5
	-14.63	1.26	4.4
	-14.30	1.59	2.5
cephalosporolide G	-2.34	0	42.9
	-2.05	0.29	26.1
	-1.66	0.68	13.5
	-1.47	0.87	9.8
	-0.95	1.40	4.1
fulvine	0.23	0	44.4
	0.69	0.45	20.8
	1.04	0.80	11.5
	1.13	0.89	9.9
	1.32	1.08	7.2
	1.79	1.56	3.2
	1.85	1.61	2.9

kurzichalcone	55.85	0	96.8
	57.89	2.04	3.1
aspernomine	80.83	0	96.5
	82.79	1.96	3.5
elevenol	71.22	0	100.0
cis-resorcylide	66.33	0	35.7
	66.97	0.25	23.6
	67.04	0.38	18.9
	67.30	0.66	11.8
	68.08	1.42	3.3
zeranol	41.76	0	53.7
	42.42	0.66	17.7
	43.39	1.62	3.5
	43.54	1.78	2.7
	43.57	1.81	2.5
solomonamide A	37.25	0	58.3
	37.88	0.63	20.1
	38.60	1.35	6.0
	38.65	1.40	5.5
	38.72	1.46	4.9

Table S2. Conformational analysis result of designed scaffolds.

scaffold		potential energy (kcal/mol)	relative energy (kcal/mol)	Boltzmann population (%)
polyheterocycle (**1**)	n=1	6.83	0	99.8
	n=2	9.53	0	97.3
medium cycle (**3**)	n=1	-36.44	0	26.2
		-36.36	0.08	22.9
		-36.29	0.15	20.4
		-36.10	0.34	14.7
		-35.49	0.95	5.2
		-35.45	0.98	5.0
		-35.08	1.36	2.7
	n=2	-34.00	0	47.1
		-33.55	0.45	21.9
		-33.54	0.46	21.7
		-32.27	1.73	2.5
		-32.26	1.74	2.5

bridged cycle (**4**)	n=1, m=1	-37.65	0	42.2
		-37.48	0.17	31.6
		-36.87	0.78	11.3
		-36.85	0.80	11.0
	n=1, m=2	-55.52	0	96.1
		-53.52	2.00	3.3
	n=2, m=1	-36.31	0	93.9
		-34.32	1.99	3.2
	n=2, m=2	-48.78	0	52.5
		-48.53	0.25	34.5
		-47.12	1.65	3.2
macrocyce (**5**)	n=1, m=1	-62.80	0	66.9
		-62.20	0.60	24.3
		-61.49	1.31	7.4
	n=1, m=2	-88.31	0	32.9
		-88.17	0.14	26.2
		-87.89	0.42	16.1
		-87.65	0.66	10.8
		-87.05	1.26	3.9
	n=2, m=1	-61.97	0	24.6
		-61.93	0.04	23.0
		-61.53	0.44	11.7
		-61.46	0.51	10.4
		-61.44	0.53	10.0
		-61.00	0.97	4.8
		-60.93	1.04	4.3
		-60.75	1.21	3.2
		-60.61	1.36	2.5
	n=2, m=2	-90.50	0	42.1
		-90.42	0.08	36.6
		-89.91	0.59	15.6

1.4 ^{1}H and ^{13}C NMR spectra

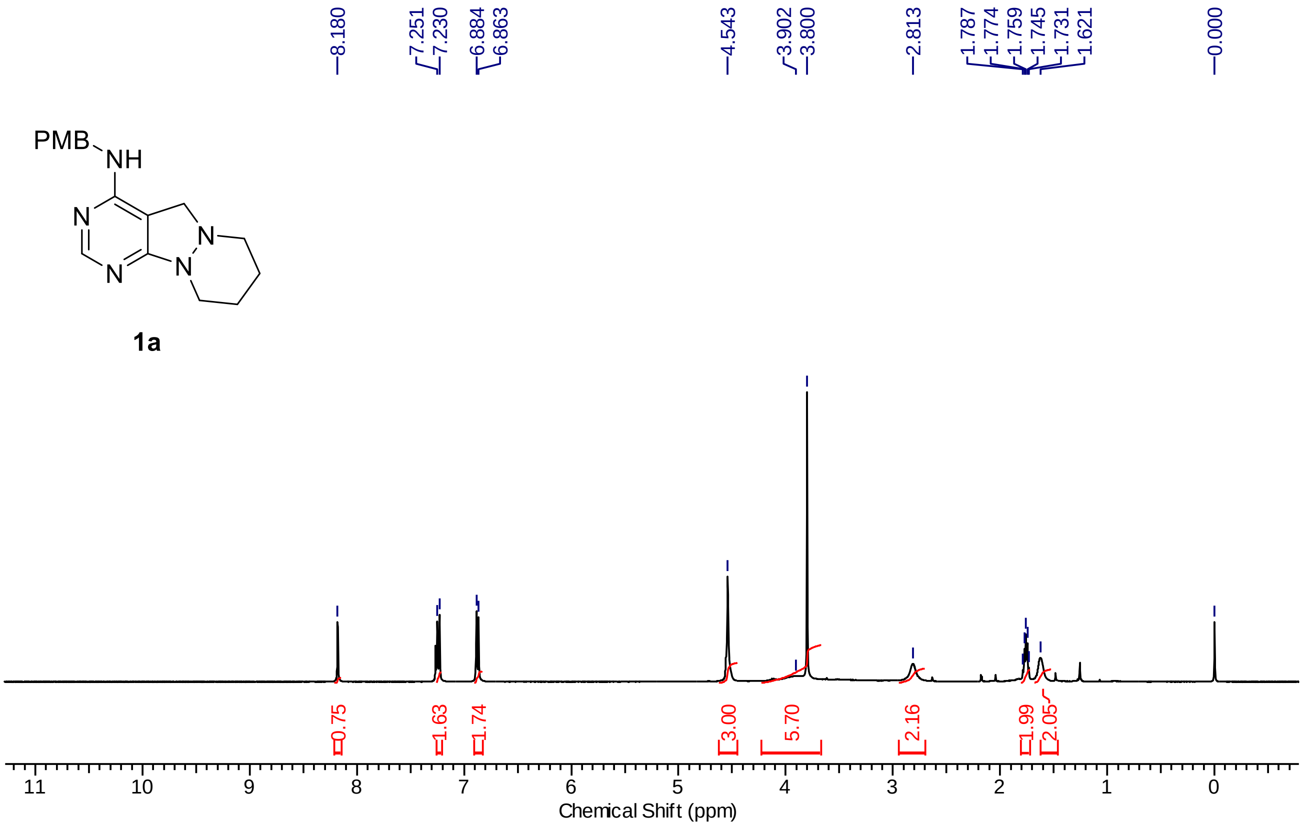

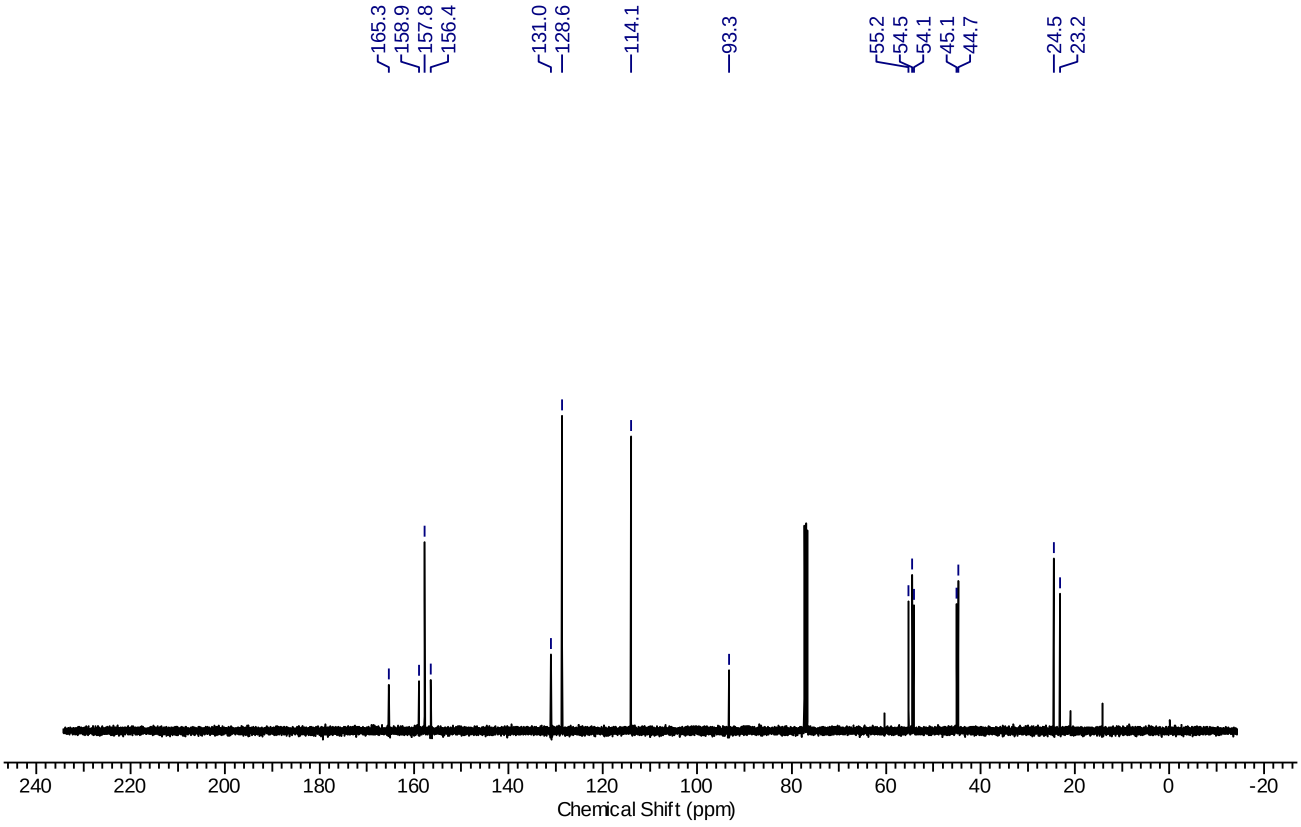

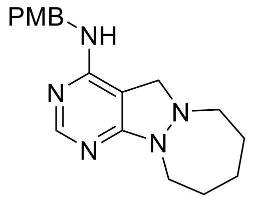
PMB
NH
N
N
N
N

1b

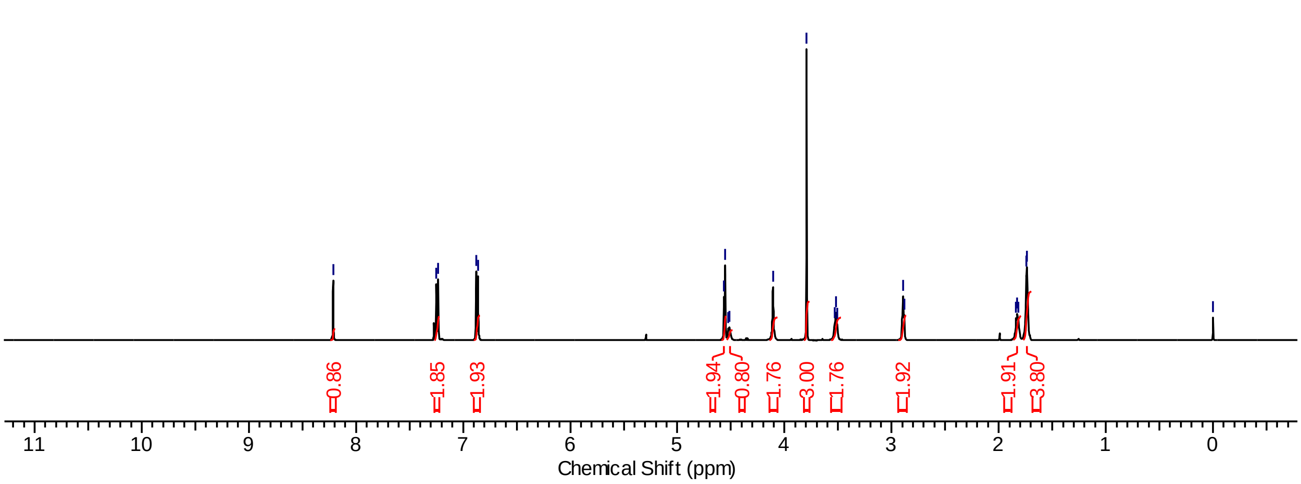
8.214
7.251
7.234
6.878
6.860
4.566
4.556
4.524
4.515
4.107
3.793
3.531
3.520
3.507
2.893
2.881
1.838
1.826
1.818
1.742
1.737
0.000
0.86
1.85
1.93
1.94
0.80
1.76
3.00
1.76
1.92
1.91
3.80
11 10 9 8 7 6 5 4 3 2 1 0
Chemical Shift (ppm)

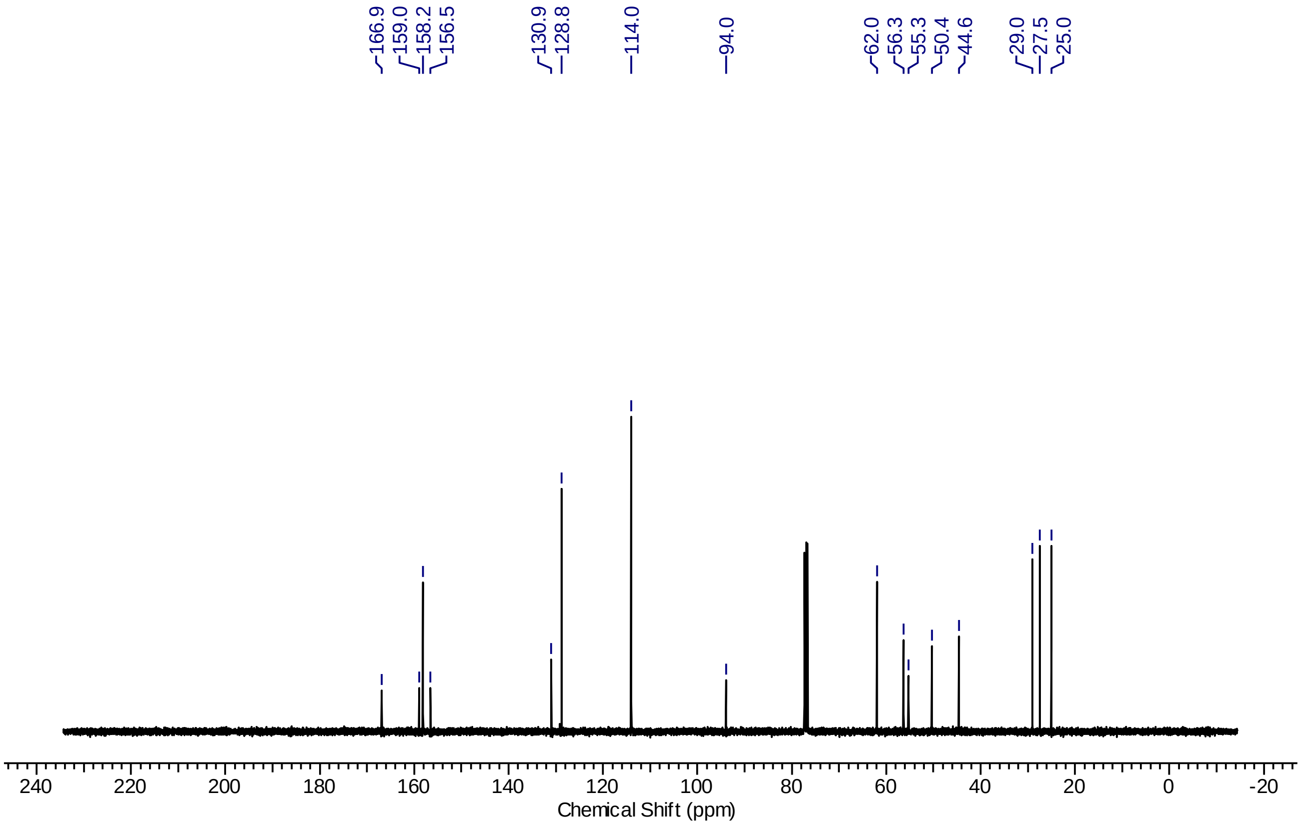
166.9
159.0
158.2
156.5
130.9
128.8
114.0
94.0
62.0
56.3
55.3
50.4
44.6
29.0
27.5
25.0
240 220 200 180 160 140 120 100 80 60 40 20 0 -20
Chemical Shift (ppm)

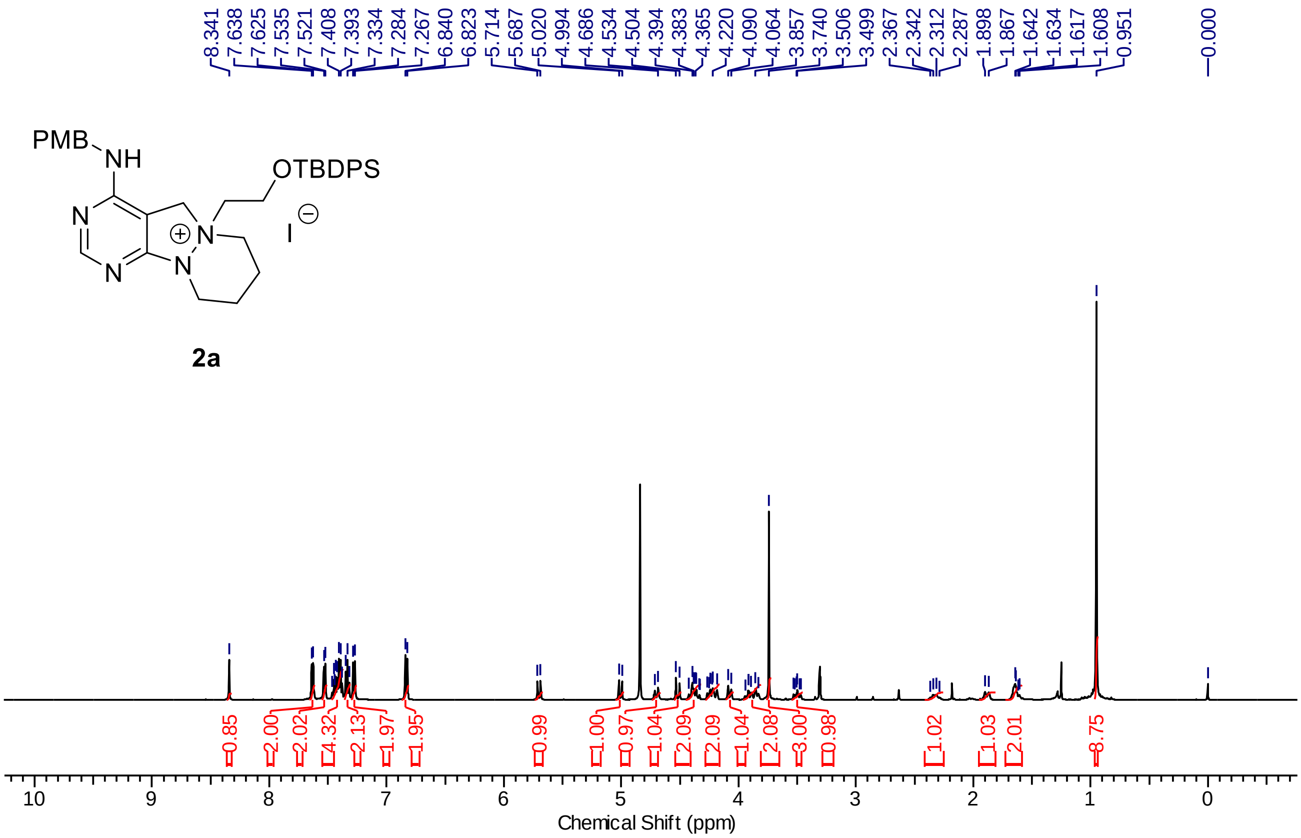
PMB
NH
OTBDPS
N
N
N
N
I
2a
8.341
7.638
7.625
7.535
7.521
7.408
7.393
7.334
7.284
7.267
6.840
6.823
5.714
5.687
5.020
4.994
4.686
4.534
4.504
4.394
4.383
4.365
4.220
4.090
4.064
3.857
3.740
3.506
3.499
2.367
2.342
2.312
2.287
1.898
1.867
1.642
1.634
1.617
1.608
0.951
0.000
0.85
2.00
2.02
4.32
2.13
1.97
1.95
0.99
1.00
0.97
1.04
2.09
2.09
1.04
2.08
3.00
0.98
1.02
1.03
2.01
8.75
Chemical Shift (ppm)

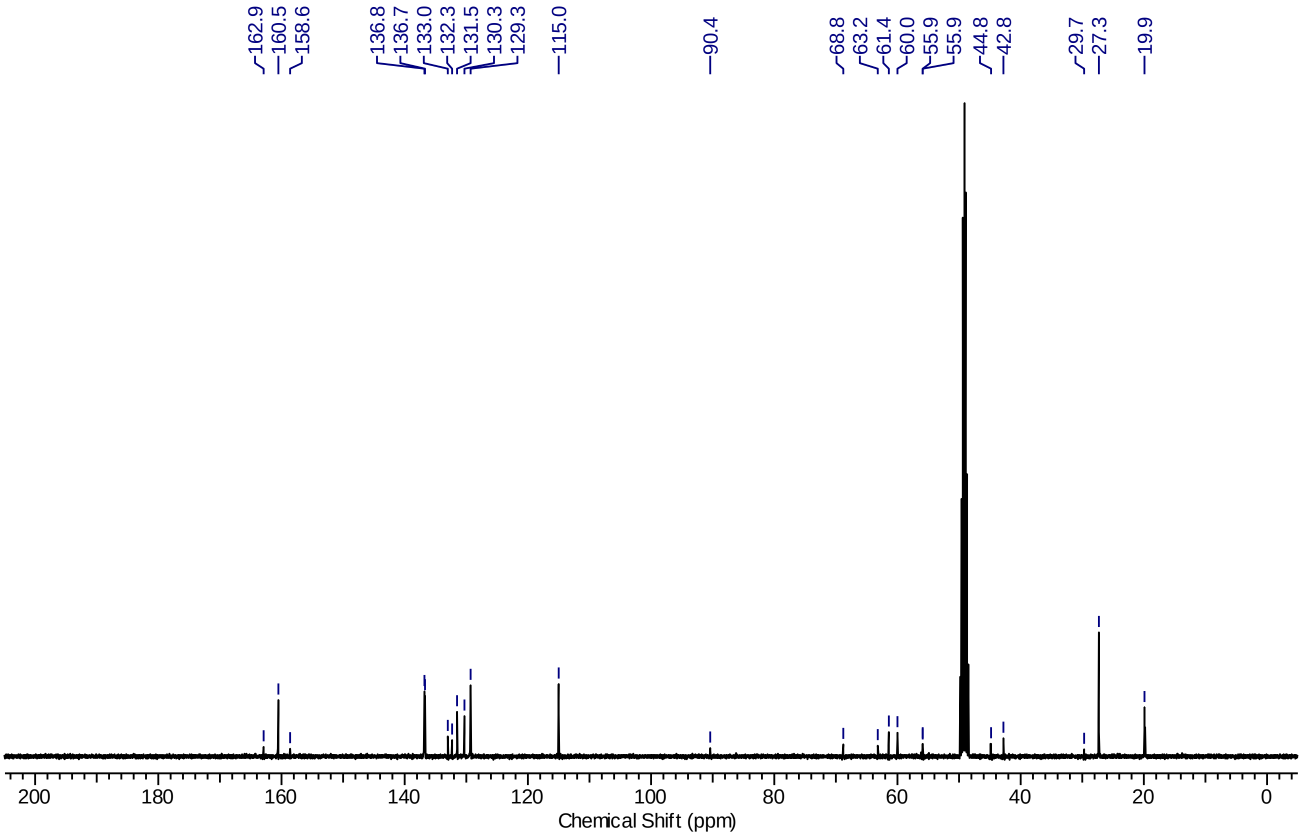
162.9
160.5
158.6
136.8
136.7
133.0
132.3
131.5
130.3
129.3
115.0
90.4
68.8
63.2
61.4
60.0
55.9
55.9
44.8
42.8
29.7
27.3
19.9
Chemical Shift (ppm)

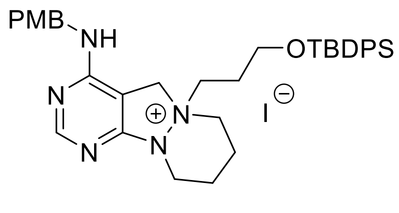
PMB
NH
N
N
N
N
OTBDPS
I

2b

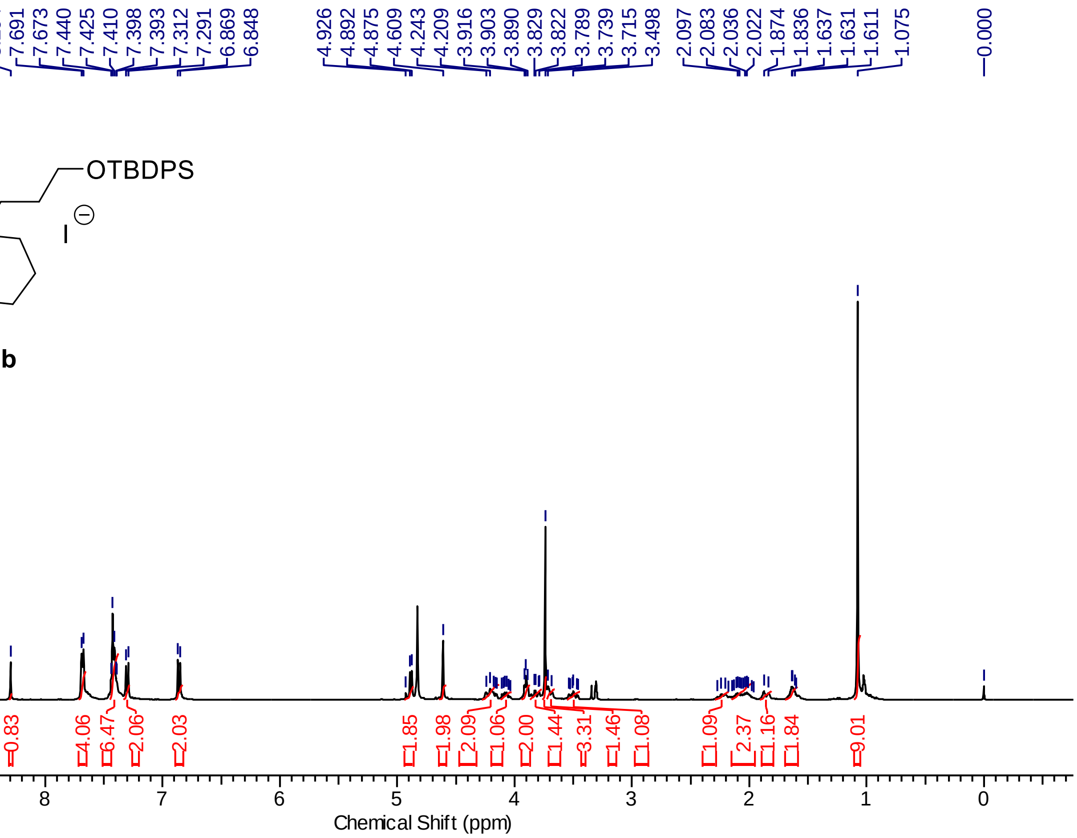
8.294
7.691
7.673
7.440
7.425
7.410
7.398
7.393
7.312
7.291
6.869
6.848
4.926
4.892
4.875
4.609
4.243
4.209
3.916
3.903
3.890
3.829
3.822
3.789
3.739
3.715
3.498
2.097
2.083
2.036
2.022
1.874
1.836
1.637
1.631
1.611
1.075
0.000
0.83
4.06
6.47
2.06
2.03
1.85
1.98
2.09
1.06
2.00
1.44
3.31
1.46
1.08
1.09
2.37
1.16
1.84
9.01
10
9
8
7
6
5
4
3
2
1
0
Chemical Shift (ppm)

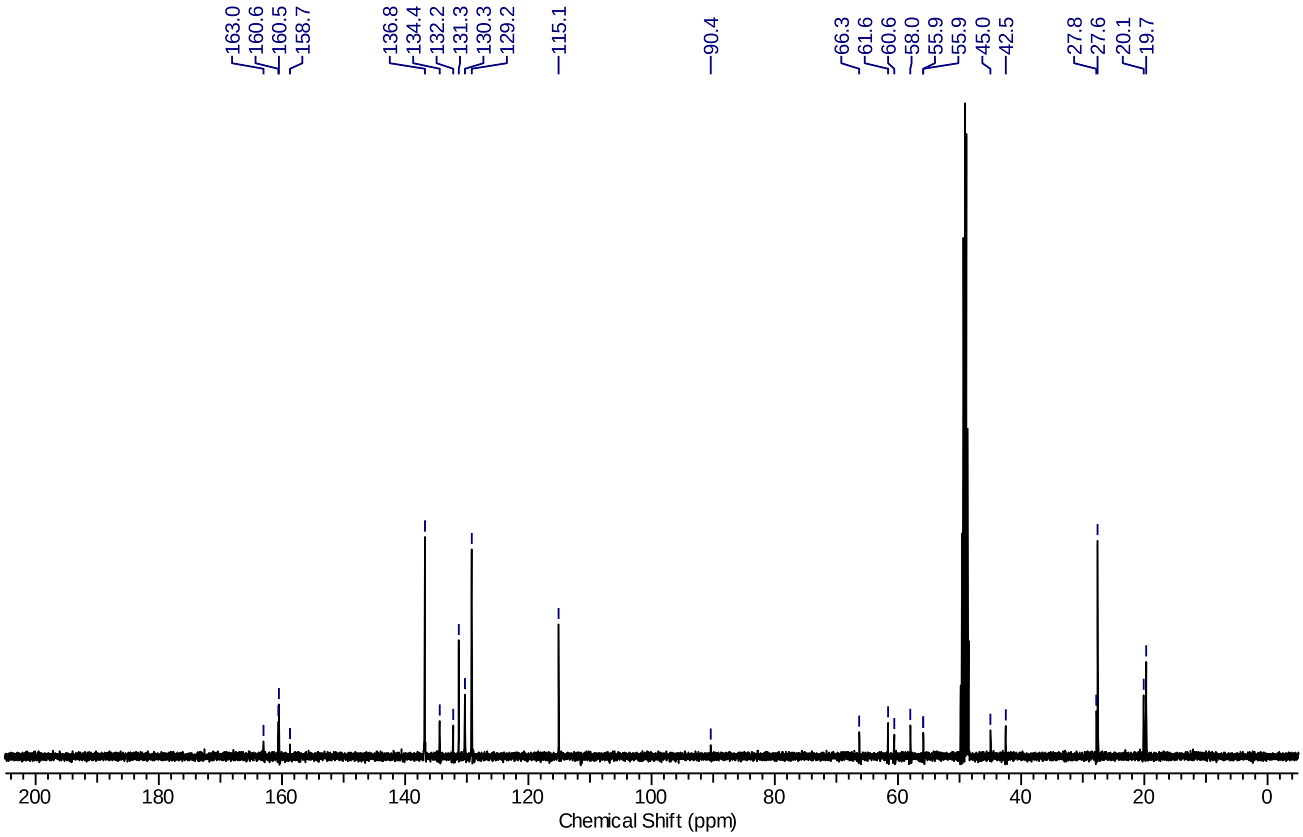
163.0
160.6
160.5
158.7
136.8
134.4
132.2
131.3
130.3
129.2
115.1
90.4
66.3
61.6
60.6
58.0
55.9
55.9
45.0
42.5
27.8
27.6
20.1
19.7
200
180
160
140
120
100
80
60
40
20
0
Chemical Shift (ppm)

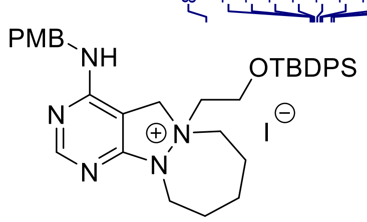
PMB
NH
OTBDPS
N
N
N
N
I

2c

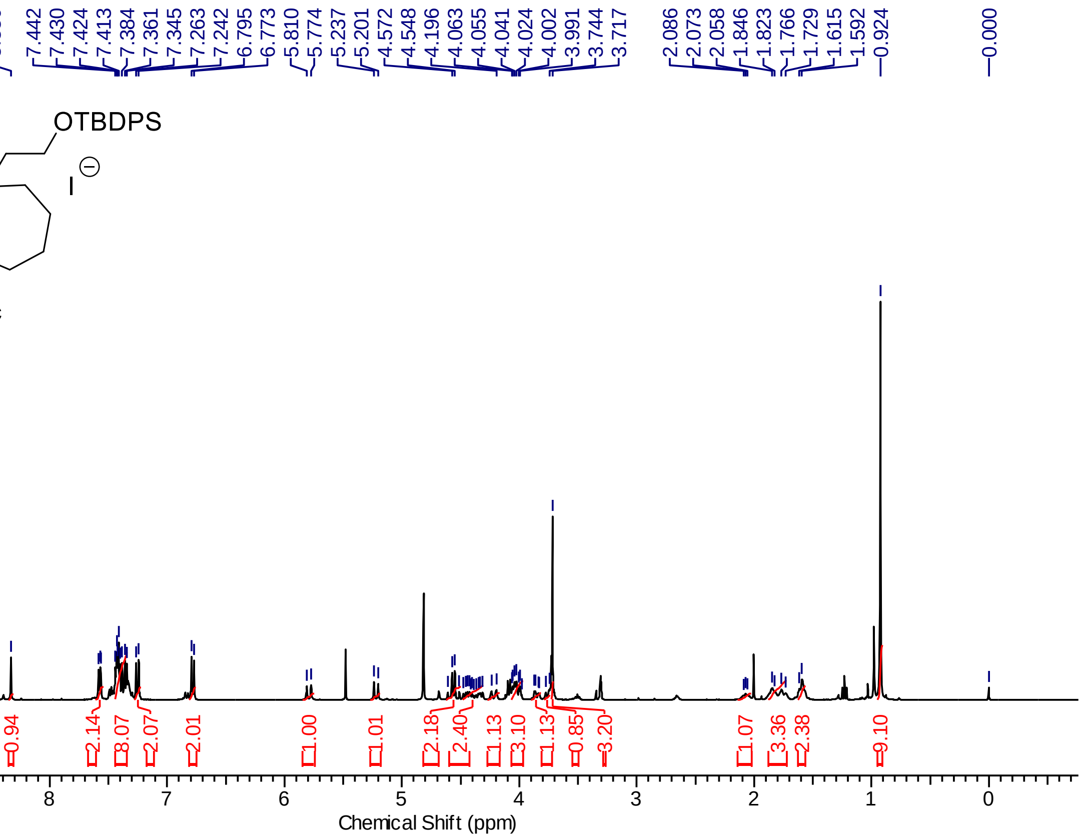
8.330
7.442
7.430
7.424
7.413
7.384
7.361
7.345
7.263
7.242
6.795
6.773
5.810
5.774
5.237
5.201
4.572
4.548
4.196
4.063
4.055
4.041
4.024
4.002
3.991
3.744
3.717
2.086
2.073
2.058
1.846
1.823
1.766
1.729
1.615
1.592
0.924
0.000
0.94
2.14
8.07
2.07
2.01
1.00
1.01
2.18
2.40
1.13
3.10
1.13
0.85
3.20
1.07
3.36
2.38
9.10
10
9
8
7
6
5
4
3
2
1
0
Chemical Shift (ppm)

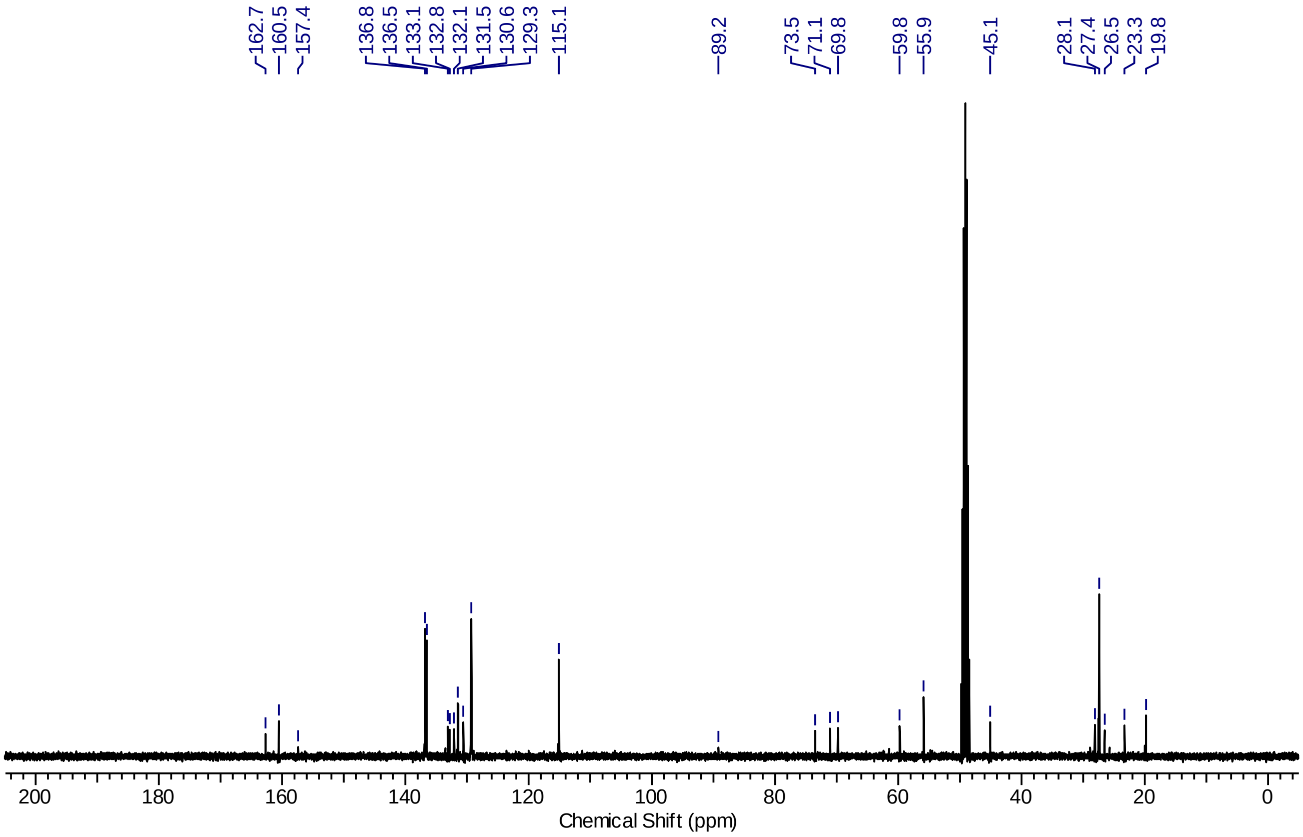
162.7
160.5
157.4
136.8
136.5
133.1
132.8
132.1
131.5
130.6
129.3
115.1
89.2
73.5
71.1
69.8
59.8
55.9
45.1
28.1
27.4
26.5
23.3
19.8
200
180
160
140
120
100
80
60
40
20
0
Chemical Shift (ppm)

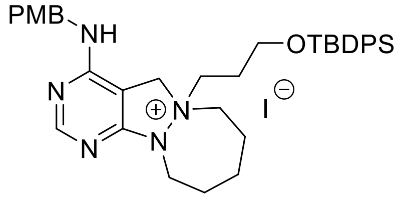

PMB
NH
OTBDPS
N
N
N
N
I

2d

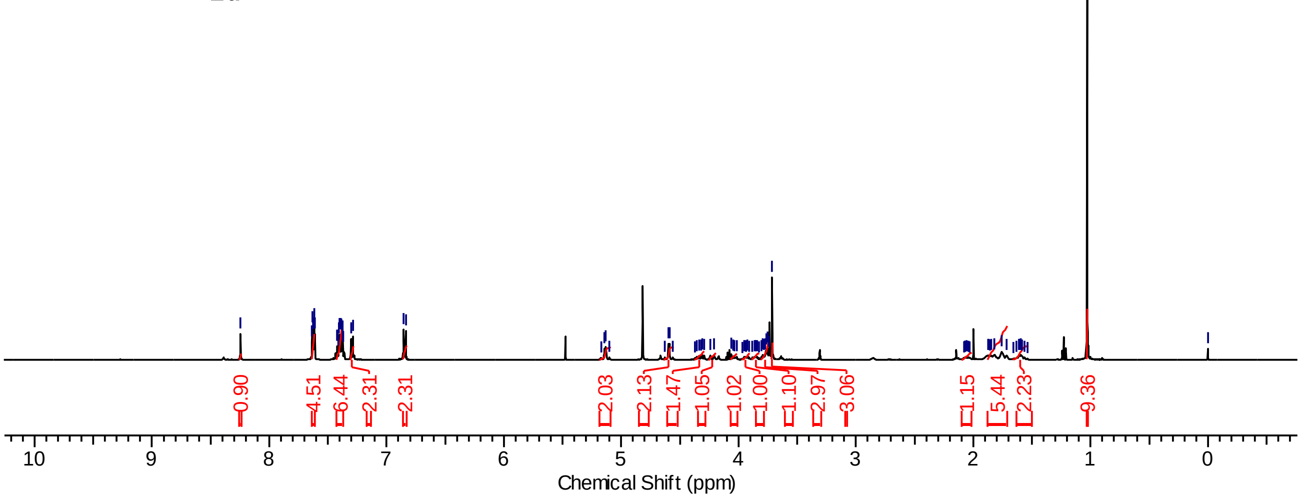

8.245 7.631 7.628 7.621 7.618 7.612 7.403 7.389 7.383 7.287 6.854 6.836
5.172 5.142 5.131 4.598 4.589 4.309 4.293 4.208 4.064 3.794 3.776 3.765 3.754 3.742 3.714
2.055 1.875 1.869 1.849 1.821 1.756 1.713 1.610 1.605 1.593 1.028
0.000
0.90 4.51 6.44 2.31 2.31 2.03 2.13 1.47 1.05 1.02 1.00 1.10 2.97 3.06 1.15 5.44 2.23 9.36
10 9 8 7 6 5 4 3 2 1 0
Chemical Shift (ppm)

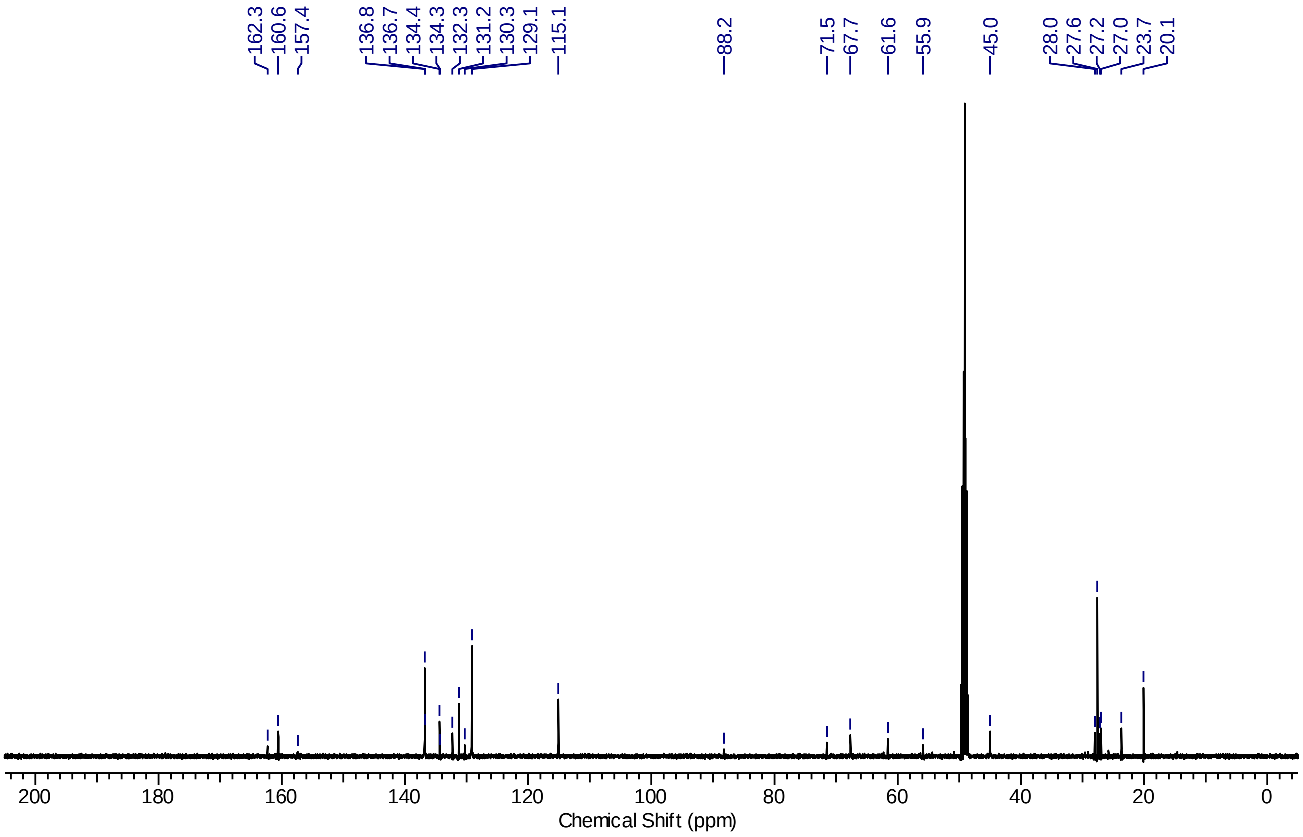

162.3 160.6 157.4
136.8 136.7 134.4 134.3 132.3 131.2 130.3 129.1 115.1
88.2
71.5 67.7 61.6 55.9 45.0
28.0 27.6 27.2 27.0 23.7 20.1
200 180 160 140 120 100 80 60 40 20 0
Chemical Shift (ppm)

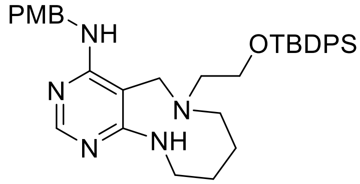
PMB
NH
OTBDPS
N
N
N
NH

3a

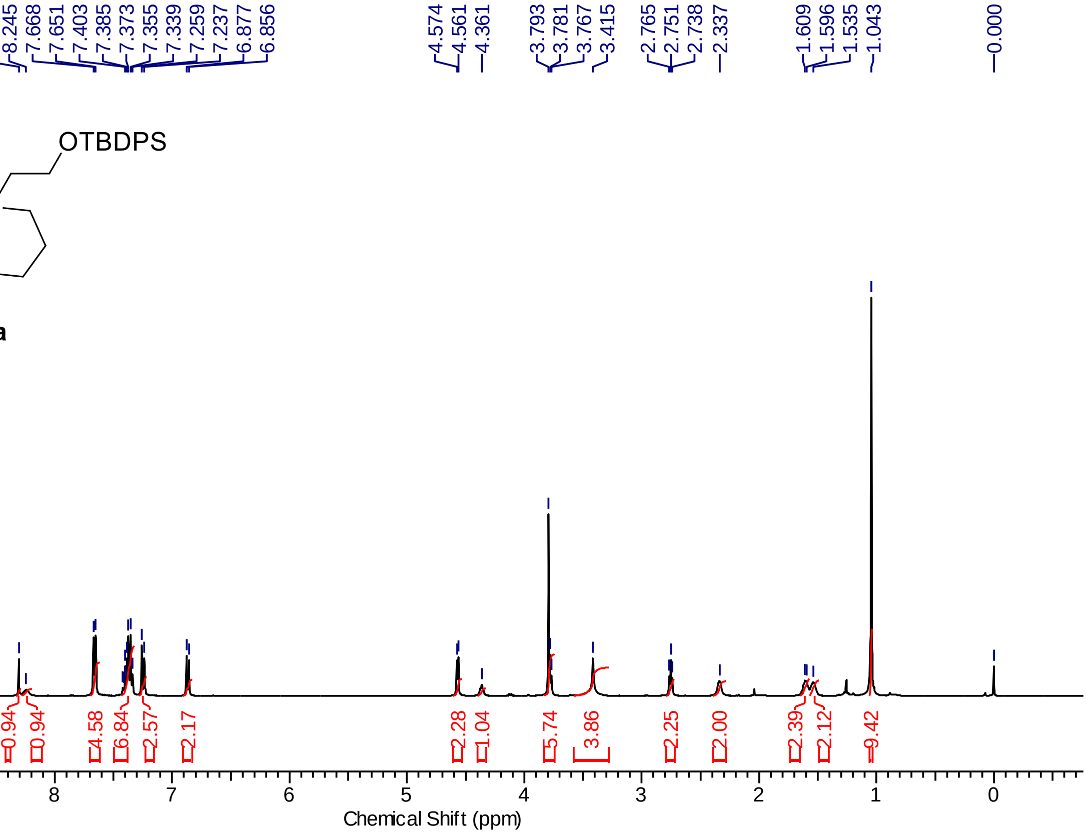
8.306
8.245
7.668
7.651
7.403
7.385
7.373
7.355
7.339
7.259
7.237
6.877
6.856
4.574
4.561
4.361
3.793
3.781
3.767
3.415
2.765
2.751
2.738
2.337
1.609
1.596
1.535
1.043
0.000
0.94
0.94
4.58
6.84
2.57
2.17
2.28
1.04
5.74
3.86
2.25
2.00
2.39
2.12
9.42
Chemical Shift (ppm)

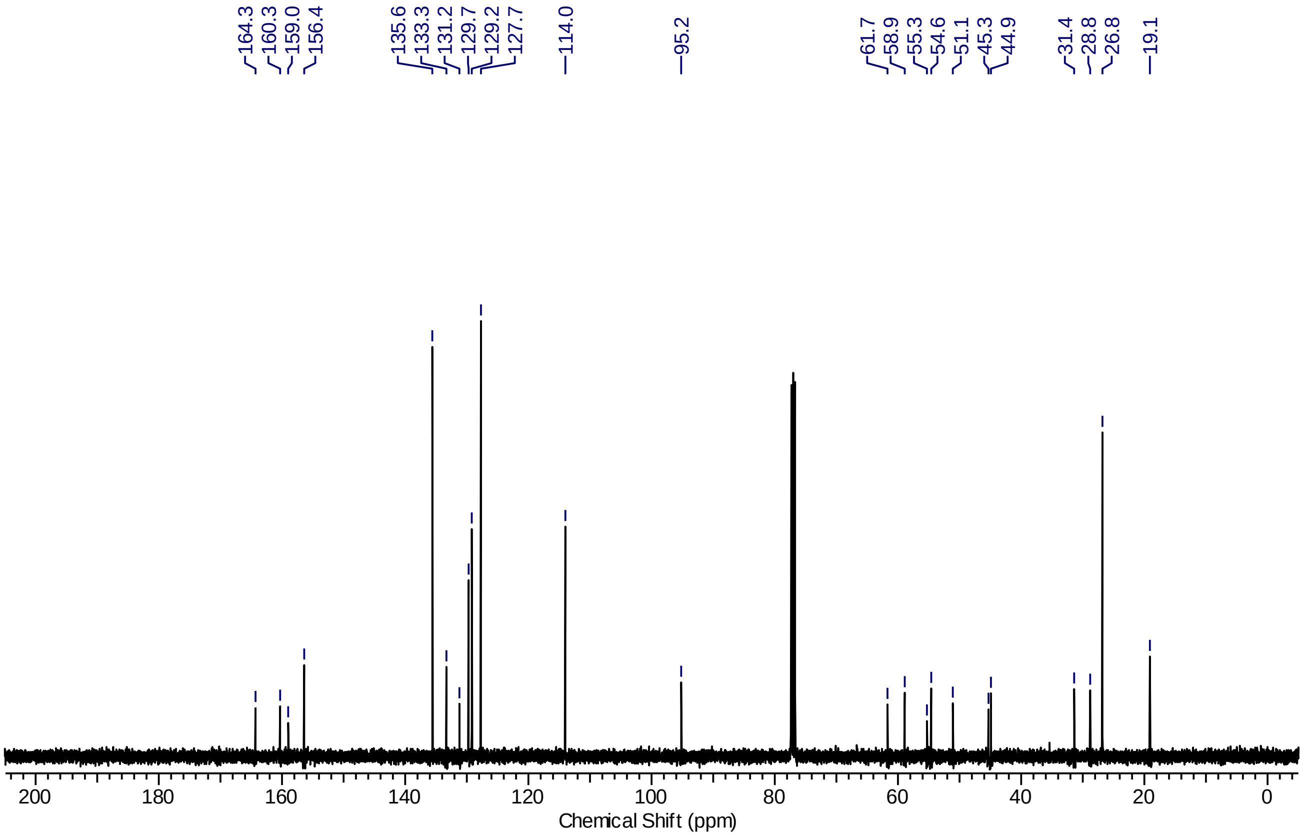
164.3
160.3
159.0
156.4
135.6
133.3
131.2
129.7
129.2
127.7
114.0
95.2
61.7
58.9
55.3
54.6
51.1
45.3
44.9
31.4
28.8
26.8
19.1
Chemical Shift (ppm)

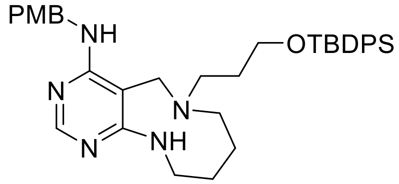
PMB
NH
OTBDPS
N
N
N
NH

3b

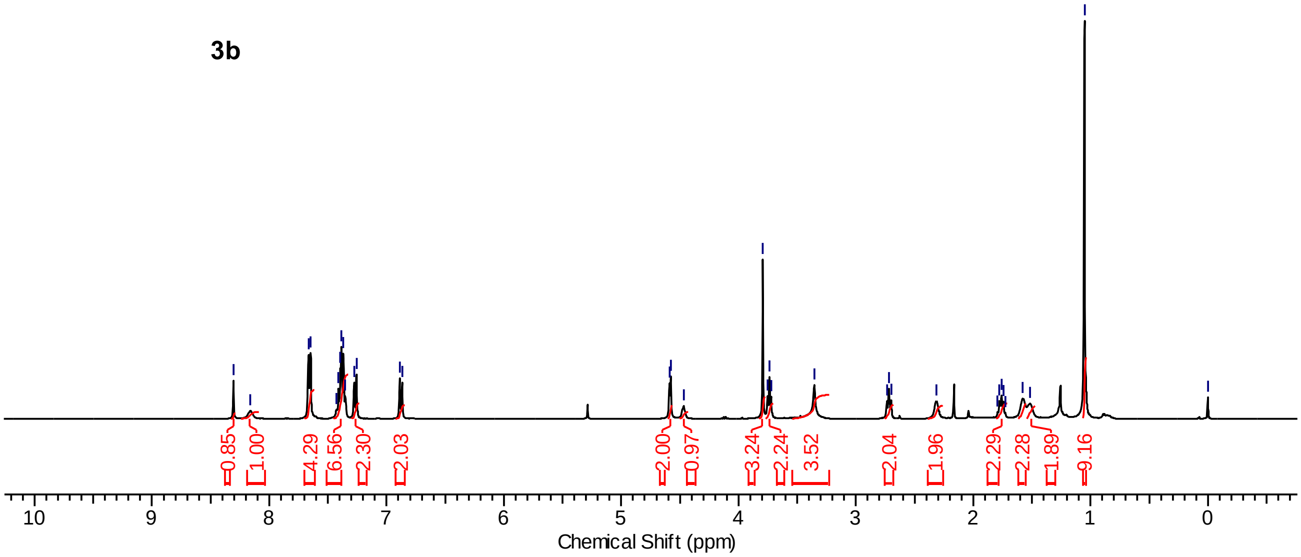
8.304
8.161
7.665
7.649
7.410
7.394
7.386
7.368
7.352
7.276
7.256
6.888
6.867
4.589
4.577
4.468
3.793
3.752
3.737
3.721
3.353
2.736
2.718
2.699
2.312
1.792
1.776
1.759
1.742
1.727
1.580
1.517
1.051
0.000
0.85
1.00
4.29
6.56
2.30
2.03
2.00
0.97
3.24
2.24
3.52
2.04
1.96
2.29
2.28
1.89
9.16
10 9 8 7 6 5 4 3 2 1 0
Chemical Shift (ppm)

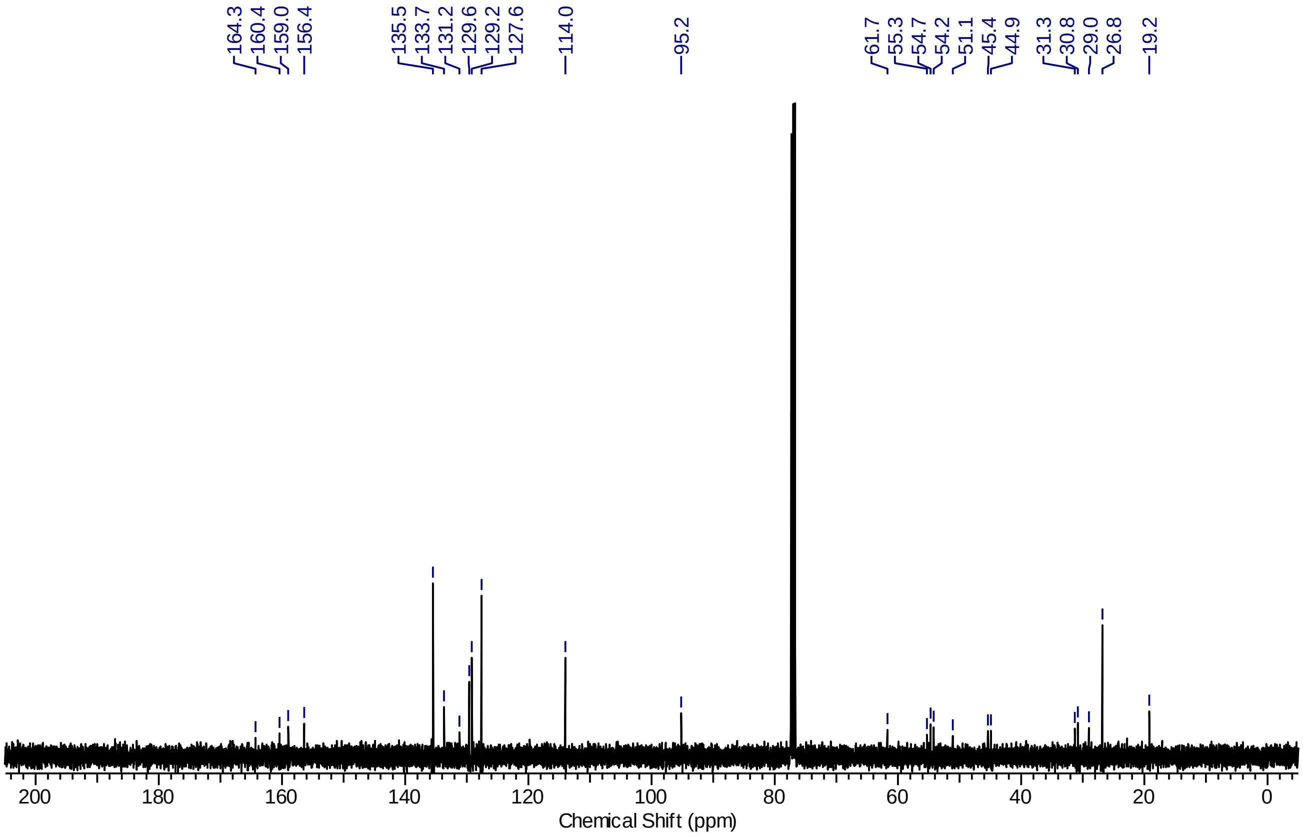
164.3
160.4
159.0
156.4
135.5
133.7
131.2
129.6
129.2
127.6
114.0
95.2
61.7
55.3
54.7
54.2
51.1
45.4
44.9
31.3
30.8
29.0
26.8
19.2
200 180 160 140 120 100 80 60 40 20 0
Chemical Shift (ppm)

3c

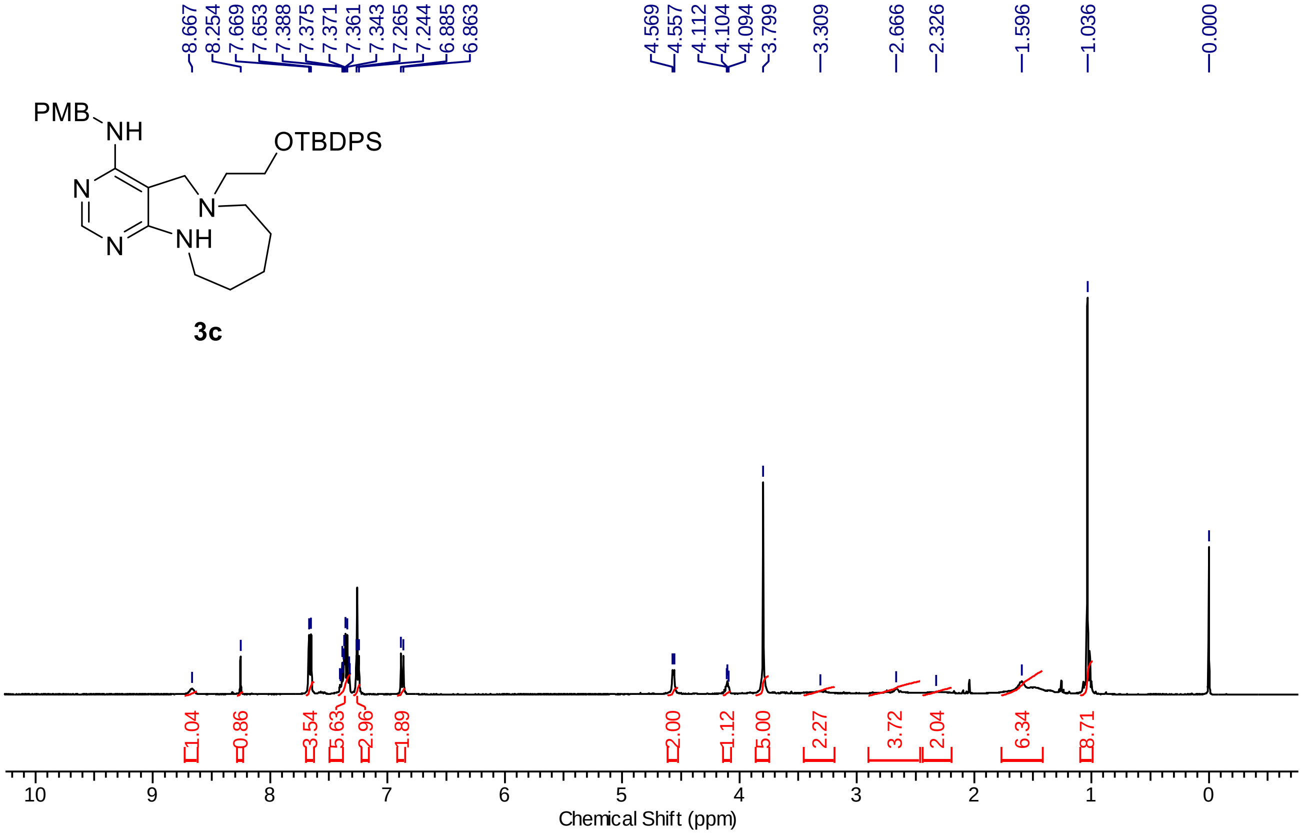
PMB
NH
OTBDPS
N
N
N
NH
8.667
8.254
7.669
7.653
7.388
7.375
7.371
7.361
7.343
7.265
7.244
6.885
6.863
4.569
4.557
4.112
4.104
4.094
3.799
3.309
2.666
2.326
1.596
1.036
0.000
1.04
0.86
3.54
5.63
2.96
1.89
2.00
1.12
5.00
2.27
3.72
2.04
6.34
8.71
Chemical Shift (ppm)

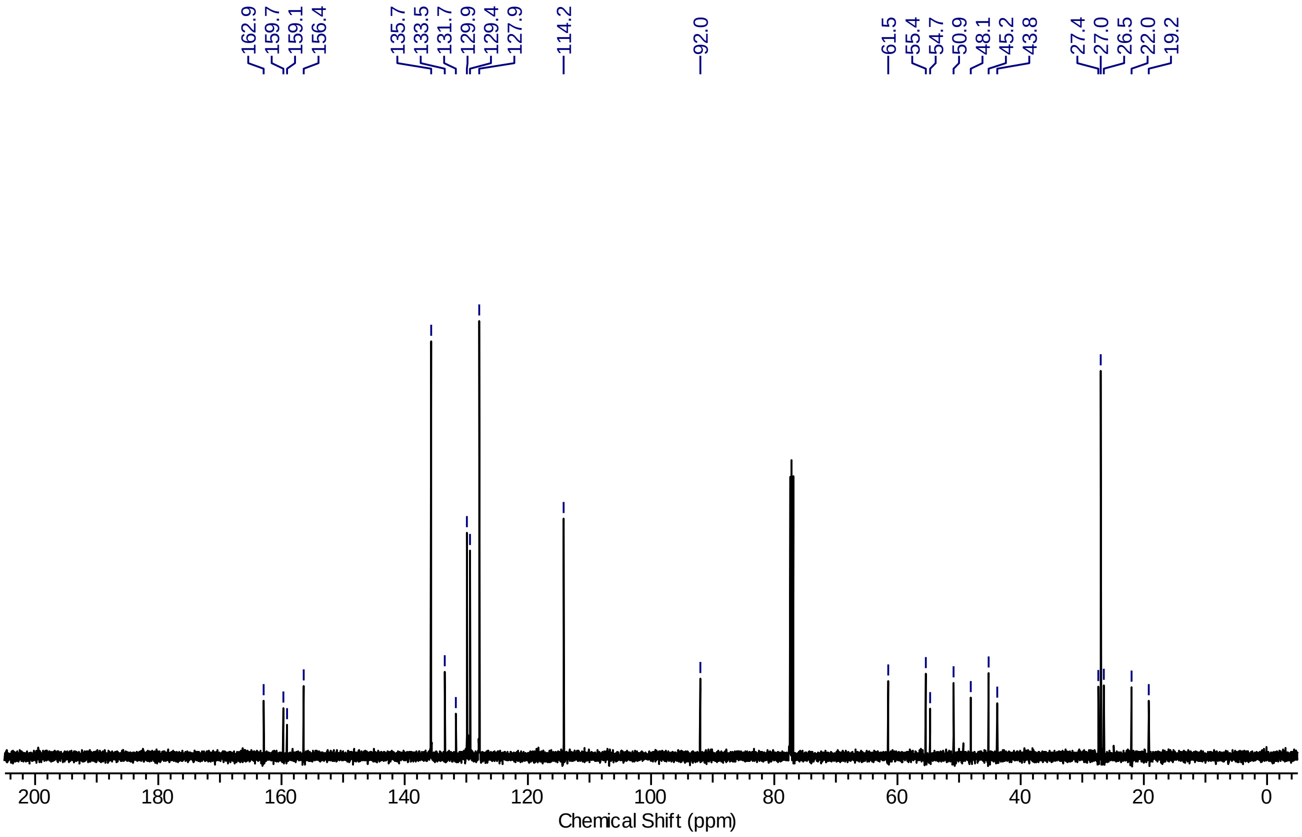
162.9
159.7
159.1
156.4
135.7
133.5
131.7
129.9
129.4
127.9
114.2
92.0
61.5
55.4
54.7
50.9
48.1
45.2
43.8
27.4
27.0
26.5
22.0
19.2
Chemical Shift (ppm)

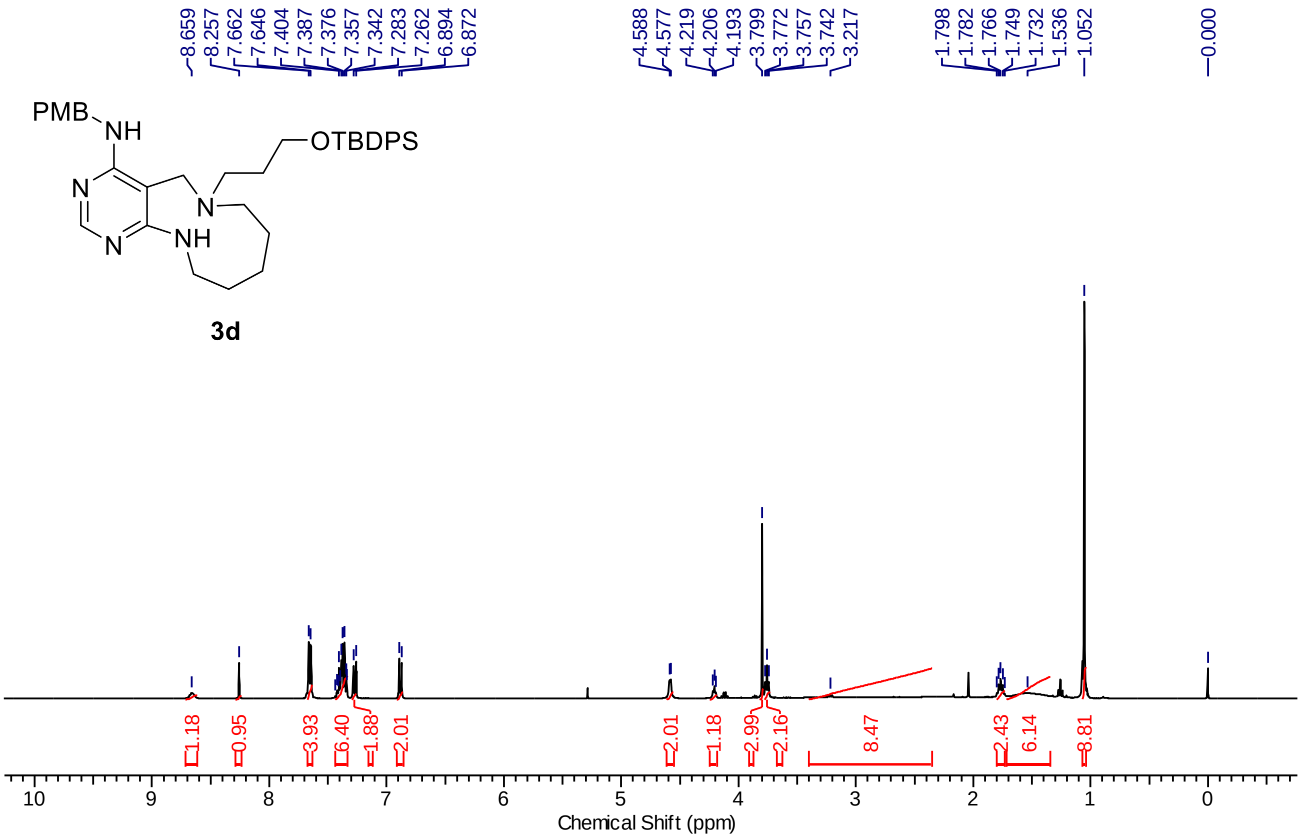
PMB
NH
OTBDPS
N
N
N
NH
3d
8.659
8.257
7.662
7.646
7.404
7.387
7.376
7.357
7.342
7.283
7.262
6.894
6.872
4.588
4.577
4.219
4.206
4.193
3.799
3.772
3.757
3.742
3.217
1.798
1.782
1.766
1.749
1.732
1.536
1.052
0.000
1.18
0.95
3.93
6.40
1.88
2.01
2.01
1.18
2.99
2.16
8.47
2.43
6.14
8.81
Chemical Shift (ppm)

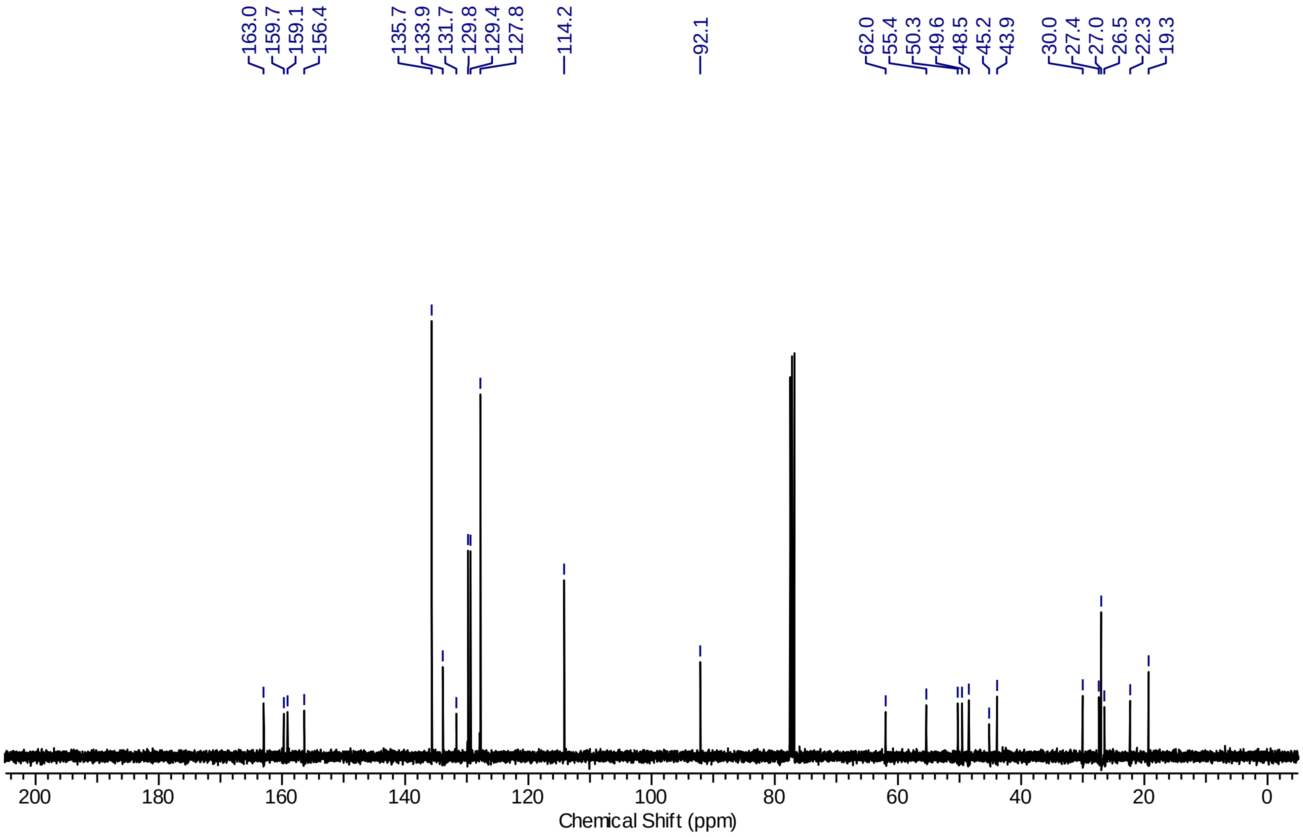
163.0
159.7
159.1
156.4
135.7
133.9
131.7
129.8
129.4
127.8
114.2
92.1
62.0
55.4
50.3
49.6
48.5
45.2
43.9
30.0
27.4
27.0
26.5
22.3
19.3
Chemical Shift (ppm)

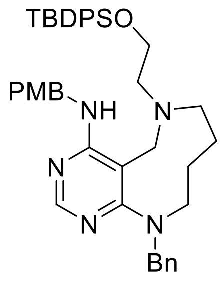
TBDPSO
PMB
NH
N
N
N
N
Bn

3a'

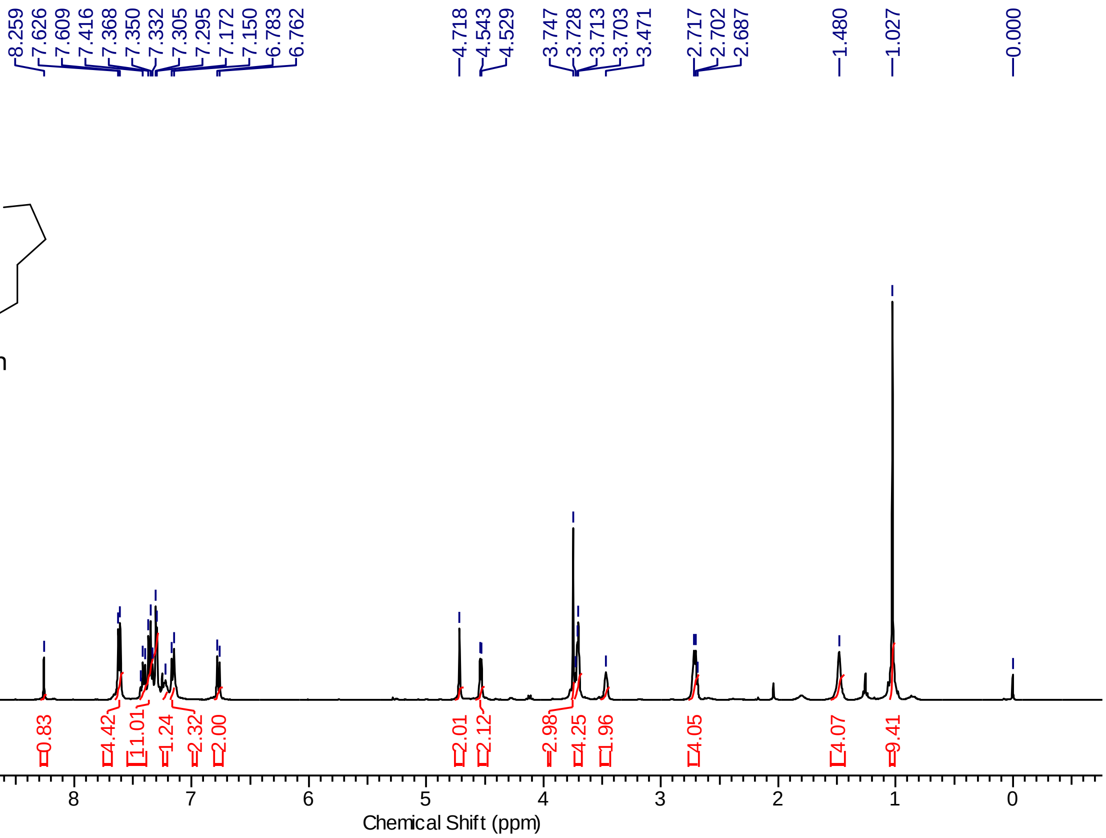
8.259
7.626
7.609
7.416
7.368
7.350
7.332
7.305
7.295
7.172
7.150
6.783
6.762
4.718
4.543
4.529
3.747
3.728
3.713
3.703
3.471
2.717
2.702
2.687
1.480
1.027
0.000
0.83
4.42
11.01
1.24
2.32
2.00
2.01
2.12
2.98
4.25
1.96
4.05
4.07
9.41
10
9
8
7
6
5
4
3
2
1
0
Chemical Shift (ppm)

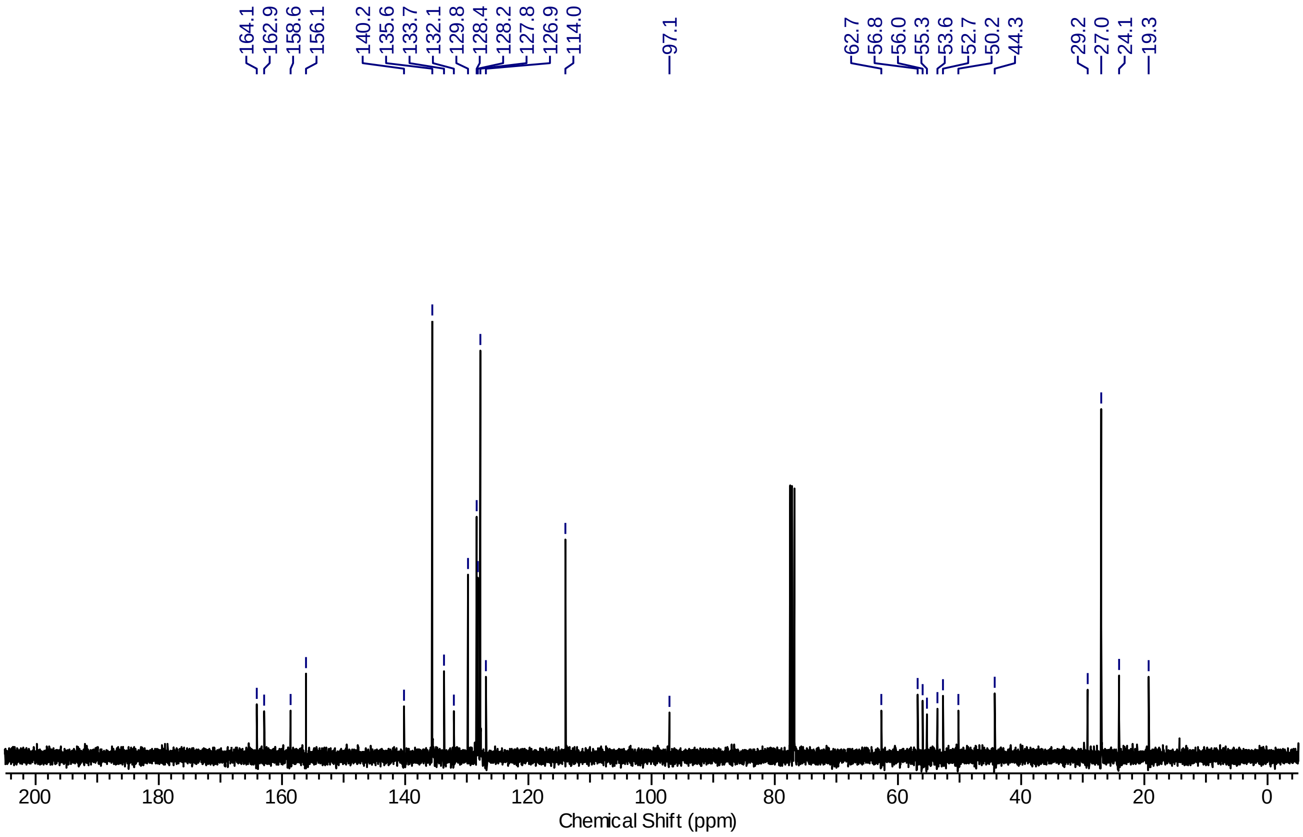
164.1
162.9
158.6
156.1
140.2
135.6
133.7
132.1
129.8
128.4
128.2
127.8
126.9
114.0
97.1
62.7
56.8
56.0
55.3
53.6
52.7
50.2
44.3
29.2
27.0
24.1
19.3
200
180
160
140
120
100
80
60
40
20
0
Chemical Shift (ppm)

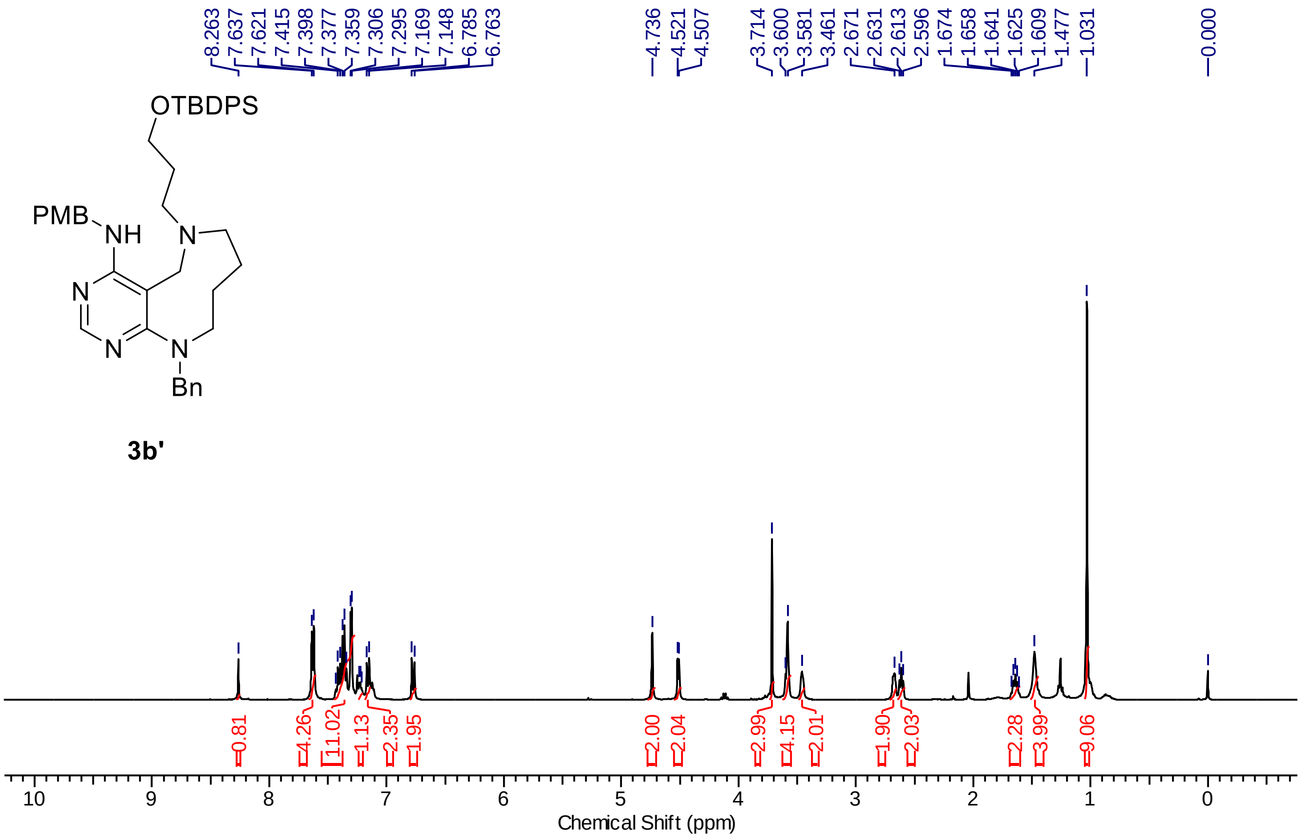
OTBDPS
PMB
NH
N
N
N
N
Bn
8.263
7.637
7.621
7.415
7.398
7.377
7.359
7.306
7.295
7.169
7.148
6.785
6.763
4.736
4.521
4.507
3.714
3.600
3.581
3.461
2.671
2.631
2.613
2.596
1.674
1.658
1.641
1.625
1.609
1.477
1.031
0.000
0.81
4.26
11.02
1.13
2.35
1.95
2.00
2.04
2.99
4.15
2.01
1.90
2.03
2.28
3.99
9.06
10
9
8
7
6
5
4
3
2
1
0
Chemical Shift (ppm)

3b'

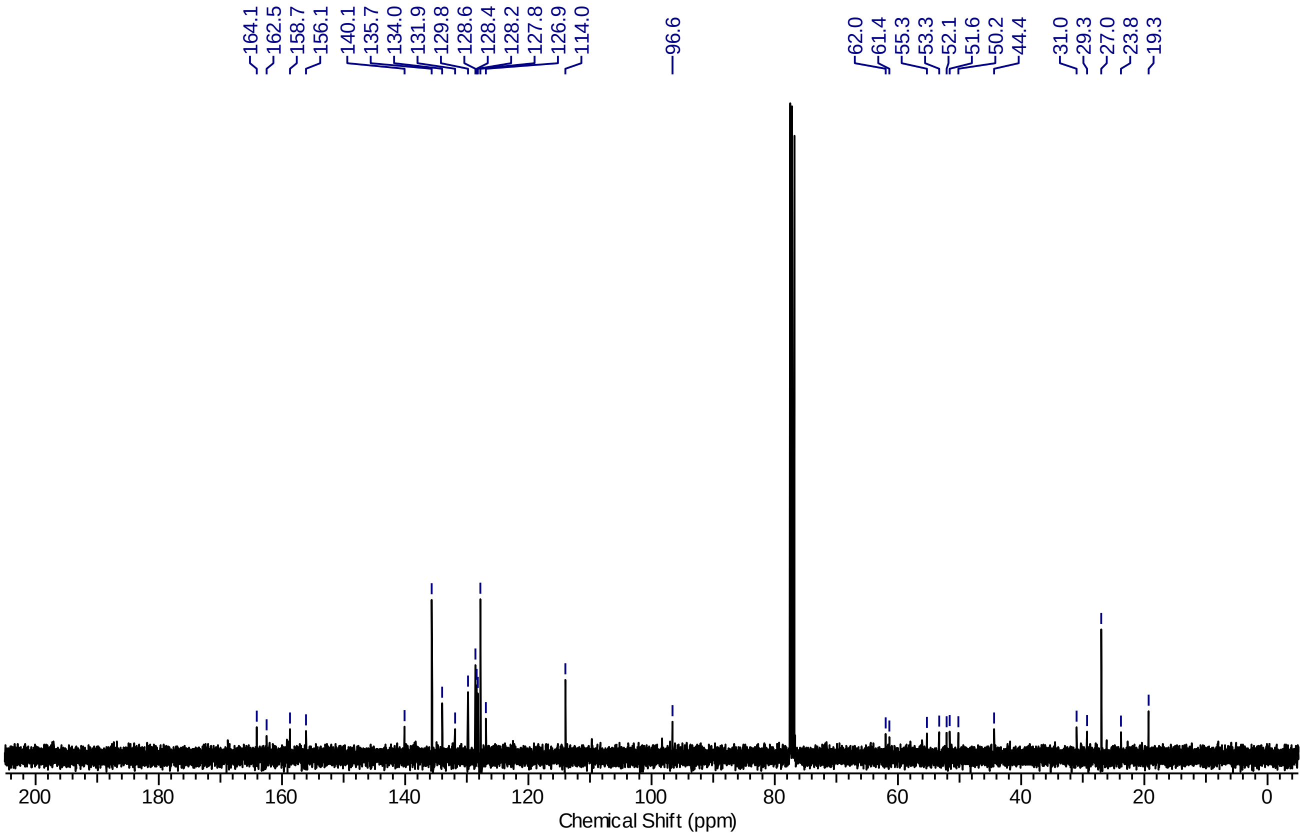
164.1
162.5
158.7
156.1
140.1
135.7
134.0
131.9
129.8
128.6
128.4
128.2
127.8
126.9
114.0
96.6
62.0
61.4
55.3
53.3
52.1
51.6
50.2
44.4
31.0
29.3
27.0
23.8
19.3
200
180
160
140
120
100
80
60
40
20
0
Chemical Shift (ppm)

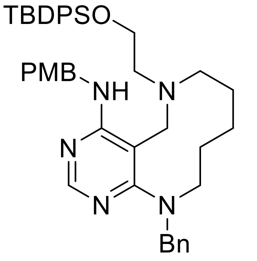
TBDPSO
PMB
NH
N
N
N
N
Bn

3c'

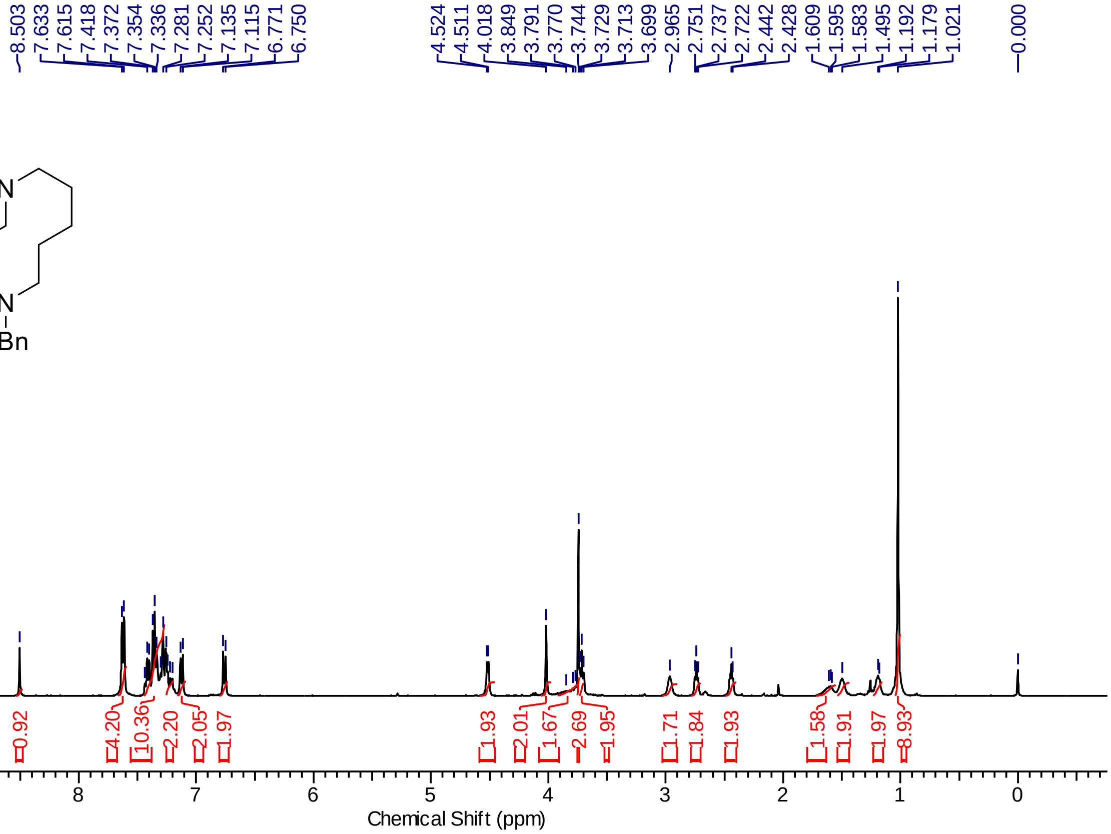
8.503
7.633
7.615
7.418
7.372
7.354
7.336
7.281
7.252
7.135
7.115
6.771
6.750
4.524
4.511
4.018
3.849
3.791
3.770
3.744
3.729
3.713
3.699
2.965
2.751
2.737
2.722
2.442
2.428
1.609
1.595
1.583
1.495
1.192
1.179
1.021
0.000
0.92
4.20
10.36
2.20
2.05
1.97
1.93
2.01
1.67
2.69
1.95
1.71
1.84
1.93
1.58
1.91
1.97
8.93
10
9
8
7
6
5
4
3
2
1
0
Chemical Shift (ppm)

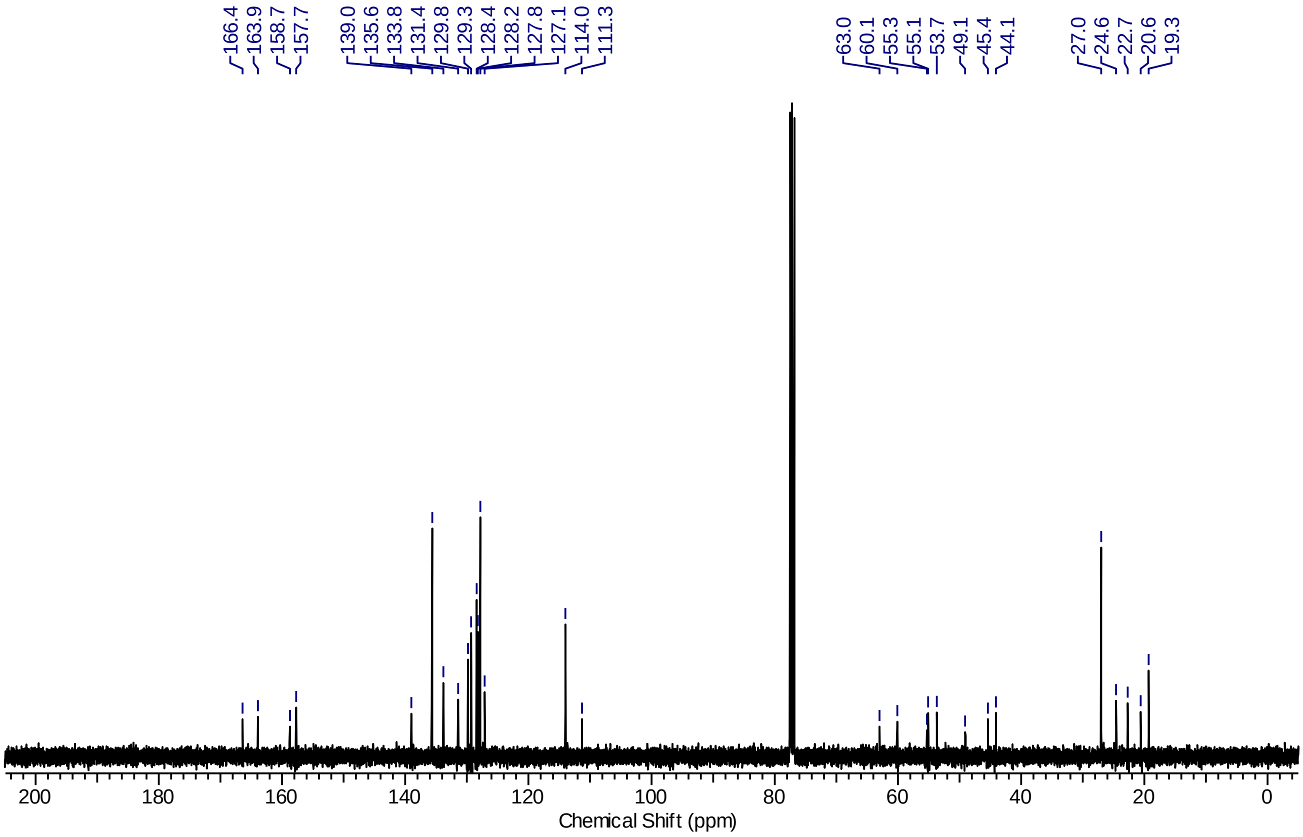
166.4
163.9
158.7
157.7
139.0
135.6
133.8
131.4
129.8
129.3
128.4
128.2
127.8
127.1
114.0
111.3
63.0
60.1
55.3
55.1
53.7
49.1
45.4
44.1
27.0
24.6
22.7
20.6
19.3
200
180
160
140
120
100
80
60
40
20
0
Chemical Shift (ppm)

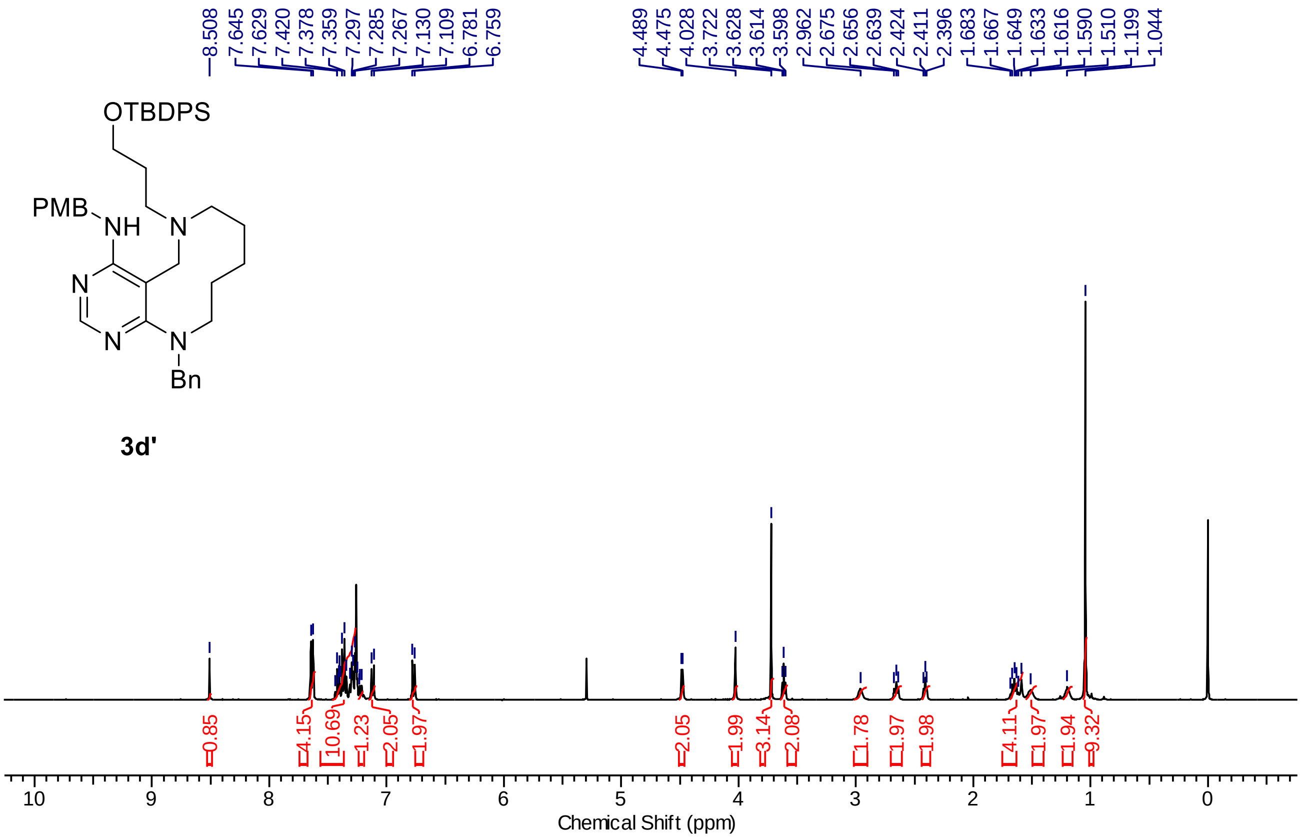
8.508
7.645
7.629
7.420
7.378
7.359
7.297
7.285
7.267
7.130
7.109
6.781
6.759
4.489
4.475
4.028
3.722
3.628
3.614
3.598
2.962
2.675
2.656
2.639
2.424
2.411
2.396
1.683
1.667
1.649
1.633
1.616
1.590
1.510
1.199
1.044
OTBDPS
PMB
NH
N
N
N
N
Bn
3d'
0.85
4.15
10.69
1.23
2.05
1.97
2.05
1.99
3.14
2.08
1.78
1.97
1.98
4.11
1.97
1.94
9.32
Chemical Shift (ppm)

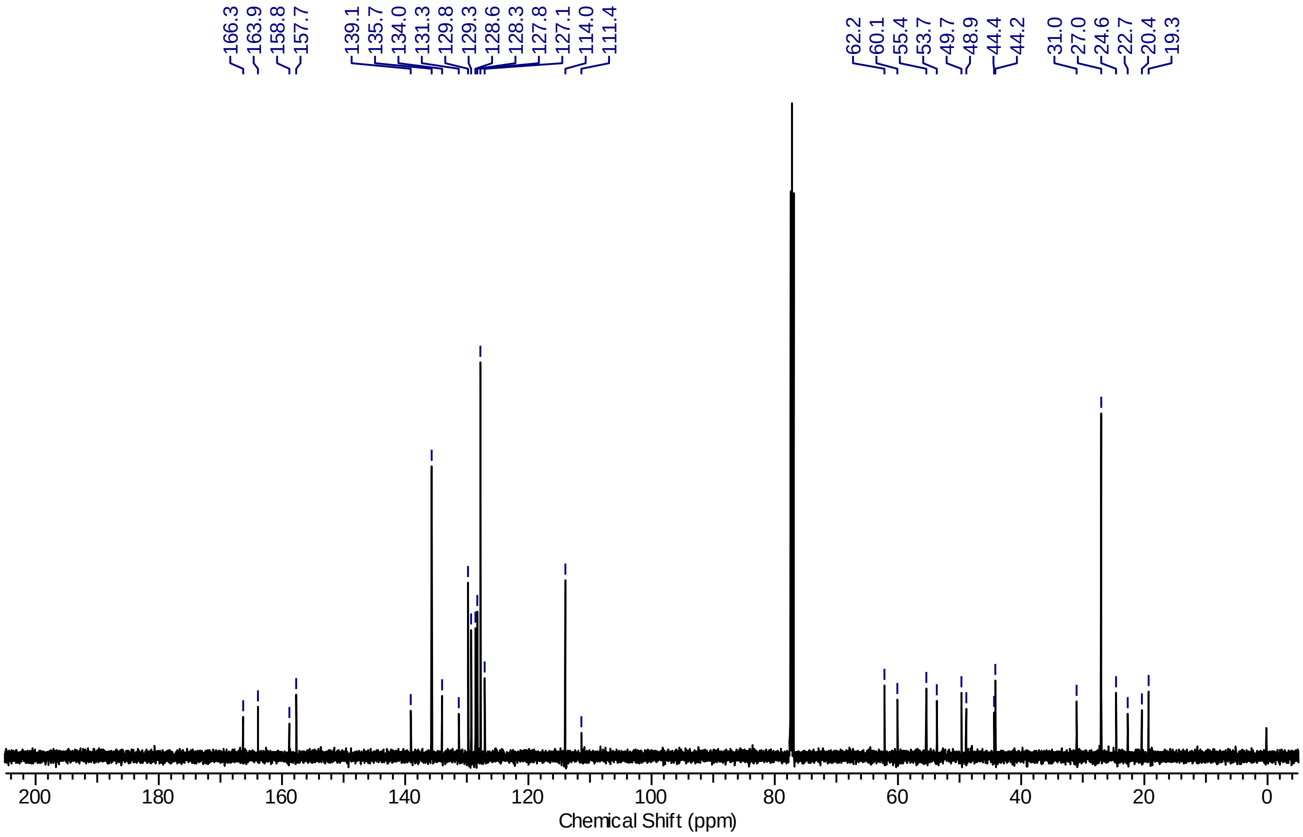
166.3
163.9
158.8
157.7
139.1
135.7
134.0
131.3
129.8
129.3
128.6
128.3
127.8
127.1
114.0
111.4
62.2
60.1
55.4
53.7
49.7
48.9
44.4
44.2
31.0
27.0
24.6
22.7
20.4
19.3
Chemical Shift (ppm)

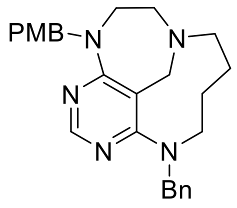
PMB
N
N
N
N
N
Bn

4a

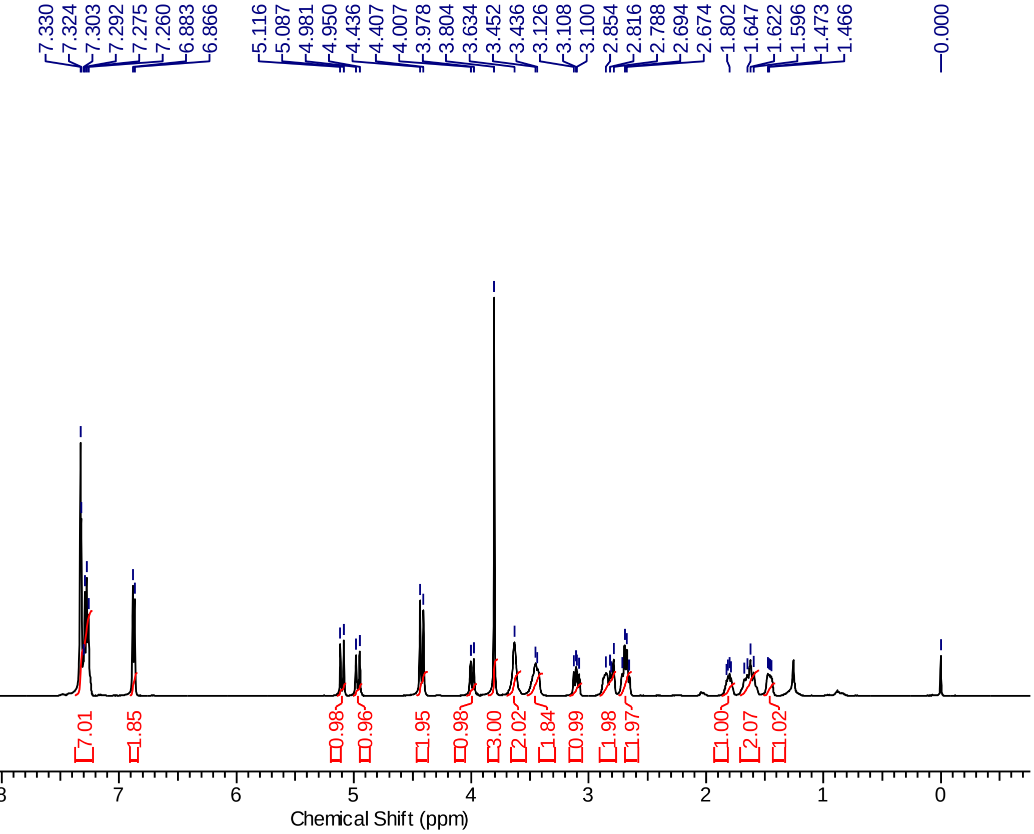
8.243
7.330
7.324
7.303
7.292
7.275
7.260
6.883
6.866
5.116
5.087
4.981
4.950
4.436
4.407
4.007
3.978
3.804
3.634
3.452
3.436
3.126
3.108
3.100
2.854
2.816
2.788
2.694
2.674
1.802
1.647
1.622
1.596
1.473
1.466
0.000
0.76
7.01
1.85
0.98
0.96
1.95
0.98
3.00
2.02
1.84
0.99
1.98
1.97
1.00
2.07
1.02
10
9
8
7
6
5
4
3
2
1
0
Chemical Shift (ppm)

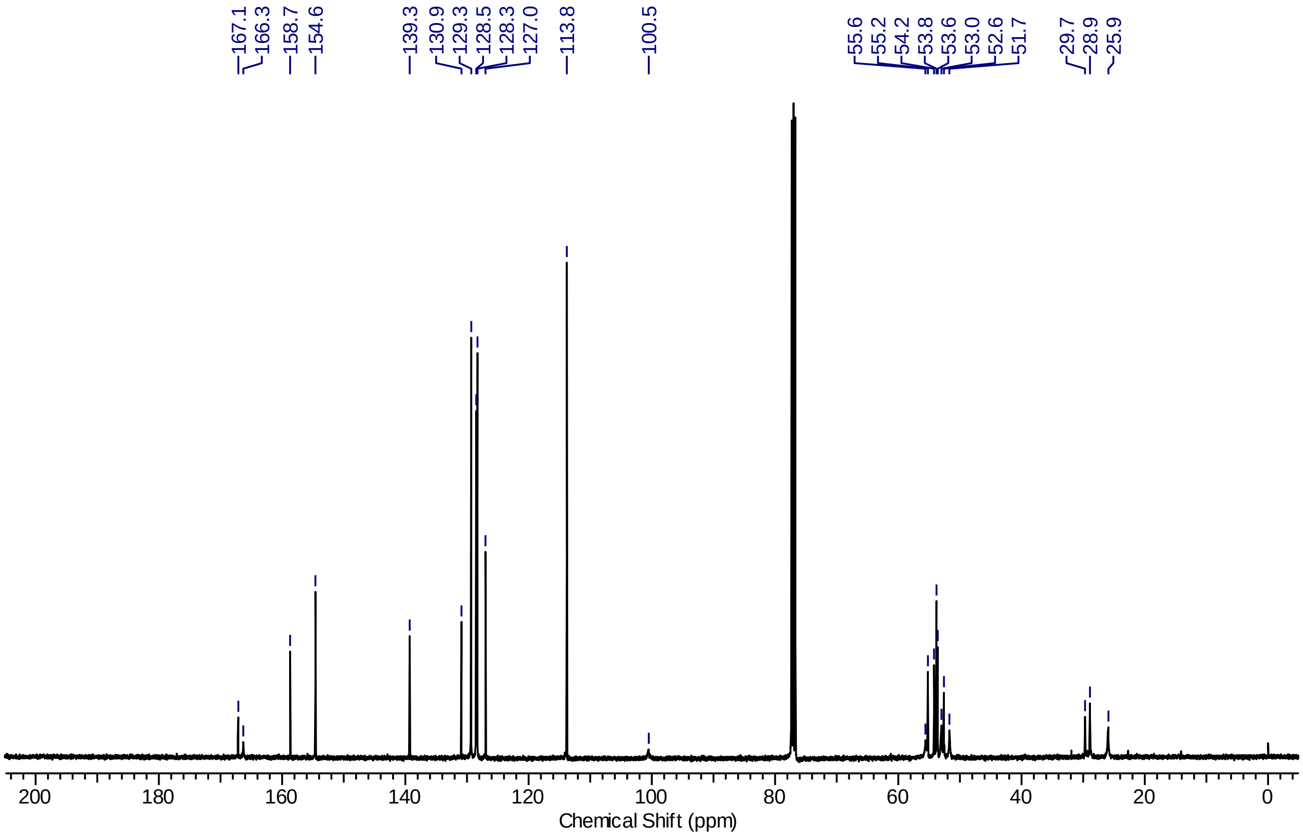
167.1
166.3
158.7
154.6
139.3
130.9
129.3
128.5
128.3
127.0
113.8
100.5
55.6
55.2
54.2
53.8
53.6
53.0
52.6
51.7
29.7
28.9
25.9
200
180
160
140
120
100
80
60
40
20
0
Chemical Shift (ppm)

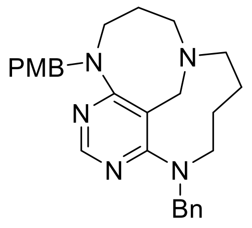
PMB
N
N
N
N
N
Bn

4b

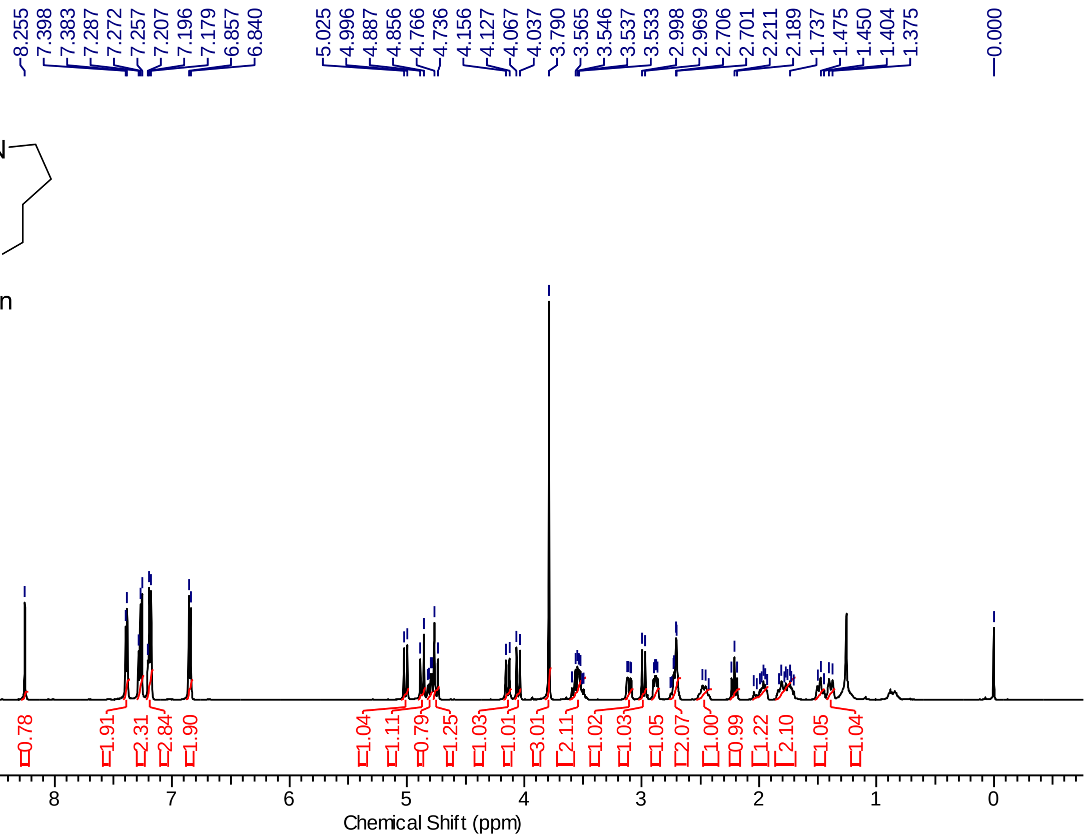
8.255
7.398
7.383
7.287
7.272
7.257
7.207
7.196
7.179
6.857
6.840
5.025
4.996
4.887
4.856
4.766
4.736
4.156
4.127
4.067
4.037
3.790
3.565
3.546
3.537
3.533
2.998
2.969
2.706
2.701
2.211
2.189
1.737
1.475
1.450
1.404
1.375
0.000
0.78
1.91
2.31
2.84
1.90
1.04
1.11
0.79
1.25
1.03
1.01
3.01
2.11
1.02
1.03
1.05
2.07
1.00
0.99
1.22
2.10
1.05
1.04
10
9
8
7
6
5
4
3
2
1
0
Chemical Shift (ppm)

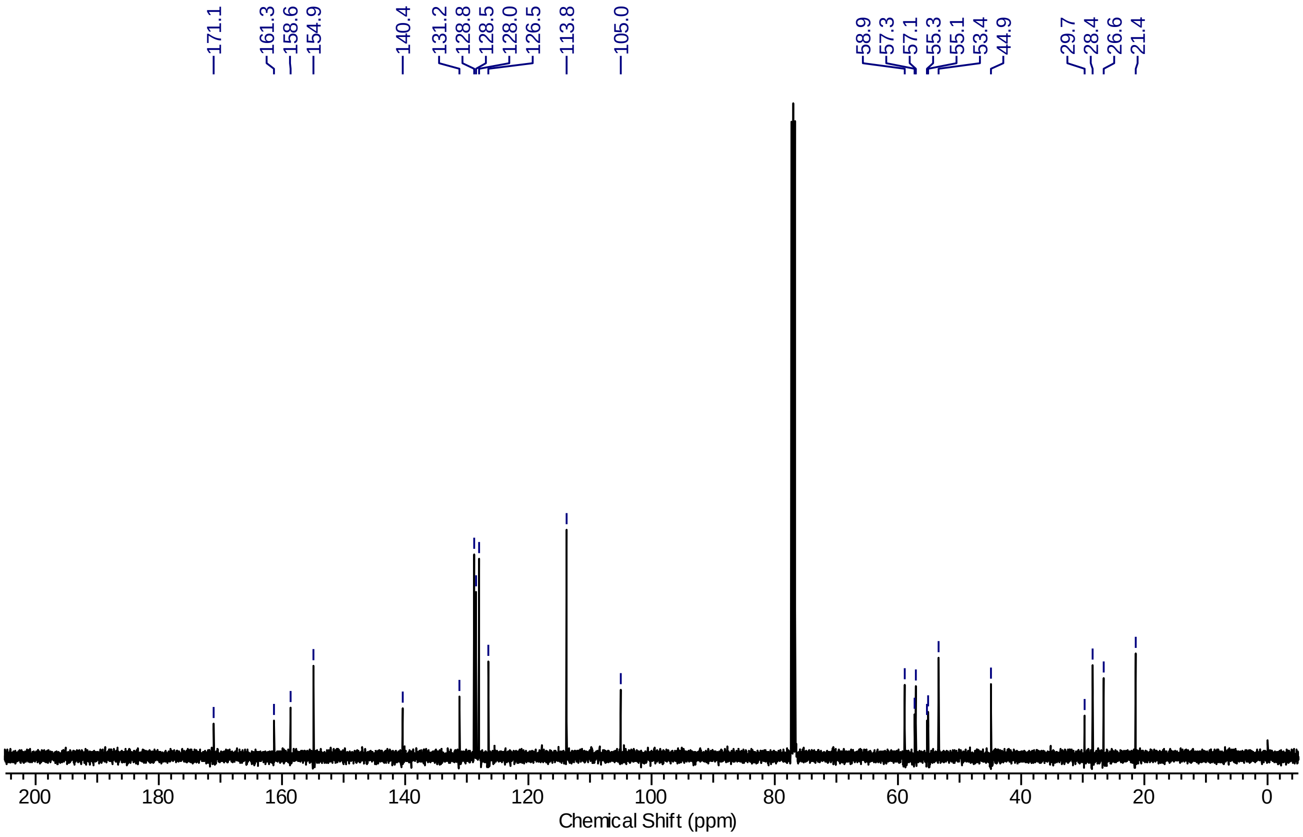
171.1
161.3
158.6
154.9
140.4
131.2
128.8
128.5
128.0
126.5
113.8
105.0
58.9
57.3
57.1
55.3
55.1
53.4
44.9
29.7
28.4
26.6
21.4
200
180
160
140
120
100
80
60
40
20
0
Chemical Shift (ppm)

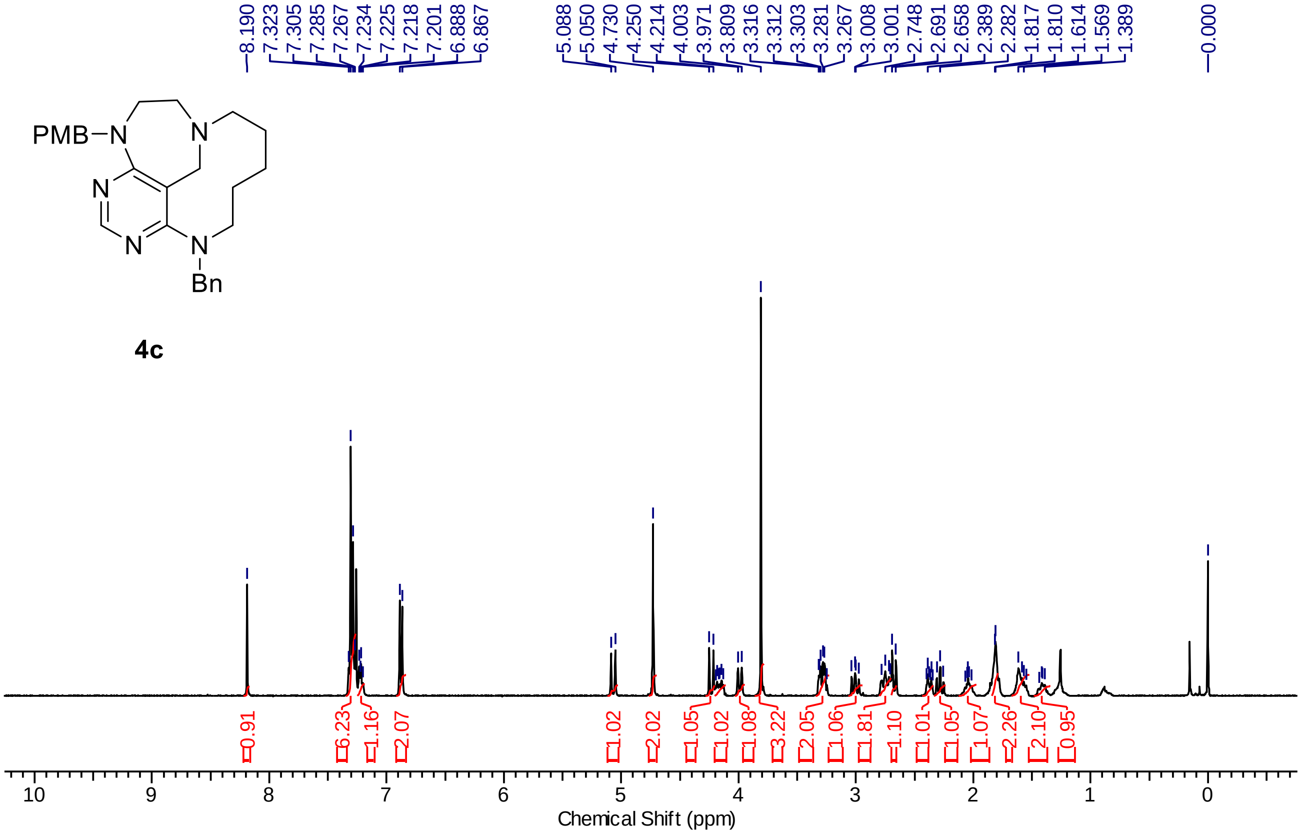
PMB–N
N
N
N
N
Bn

4c

8.190
7.323
7.305
7.285
7.267
7.234
7.225
7.218
7.201
6.888
6.867
5.088
5.050
4.730
4.250
4.214
4.003
3.971
3.809
3.316
3.312
3.303
3.281
3.267
3.008
3.001
2.748
2.691
2.658
2.389
2.282
1.817
1.810
1.614
1.569
1.389
0.000
0.91
6.23
1.16
2.07
1.02
2.02
1.05
1.02
1.08
3.22
2.05
1.06
1.81
1.10
1.01
1.05
1.07
2.26
2.10
0.95
10 9 8 7 6 5 4 3 2 1 0
Chemical Shift (ppm)

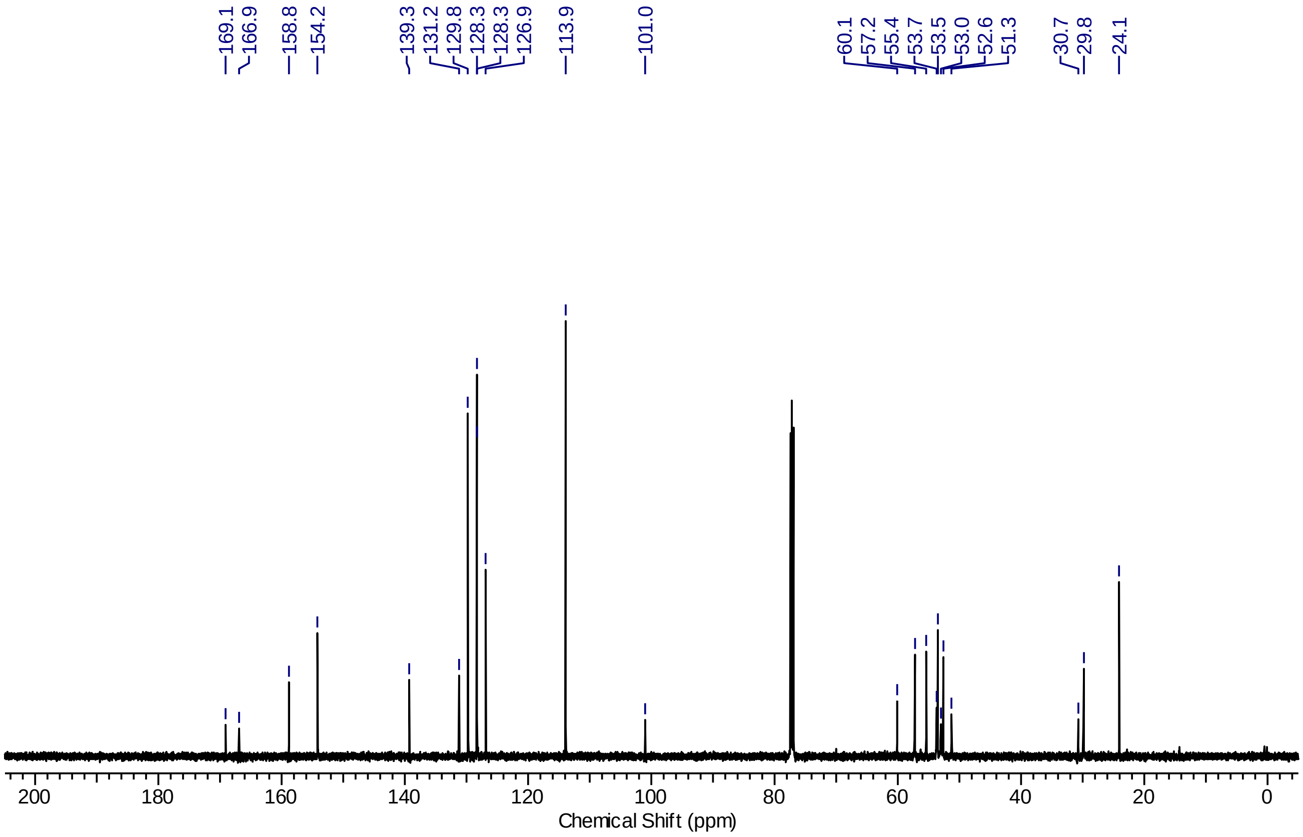
169.1
166.9
158.8
154.2
139.3
131.2
129.8
128.3
128.3
126.9
113.9
101.0
60.1
57.2
55.4
53.7
53.5
53.0
52.6
51.3
30.7
29.8
24.1
200 180 160 140 120 100 80 60 40 20 0
Chemical Shift (ppm)

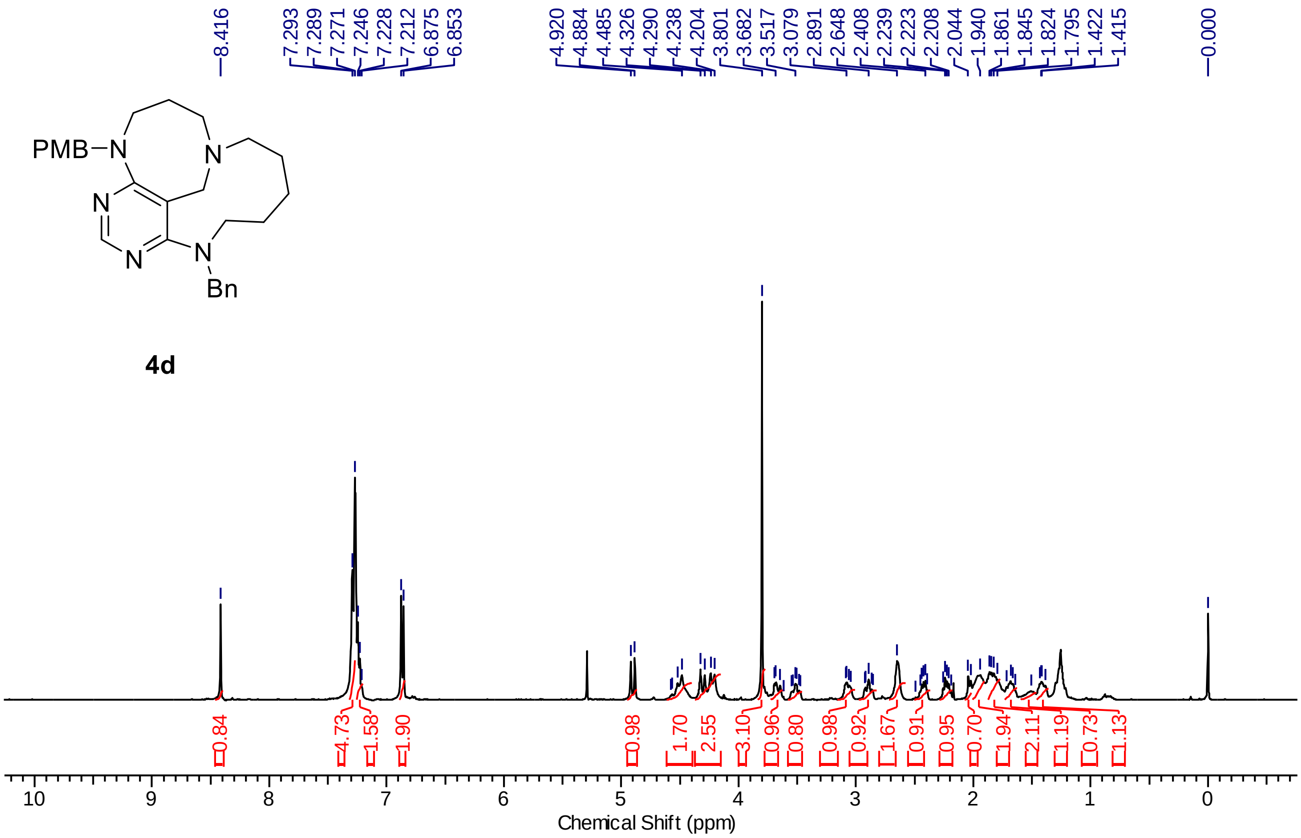
8.416
7.293
7.289
7.271
7.246
7.228
7.212
6.875
6.853
4.920
4.884
4.485
4.326
4.290
4.238
4.204
3.801
3.682
3.517
3.079
2.891
2.648
2.408
2.239
2.223
2.208
2.044
1.940
1.861
1.845
1.824
1.795
1.422
1.415
0.000
PMB
N
N
N
N
N
Bn
4d
0.84
4.73
1.58
1.90
0.98
1.70
2.55
3.10
0.96
0.80
0.98
0.92
1.67
0.91
0.95
0.70
1.94
2.11
1.19
0.73
1.13
10
9
8
7
6
5
4
3
2
1
0
Chemical Shift (ppm)

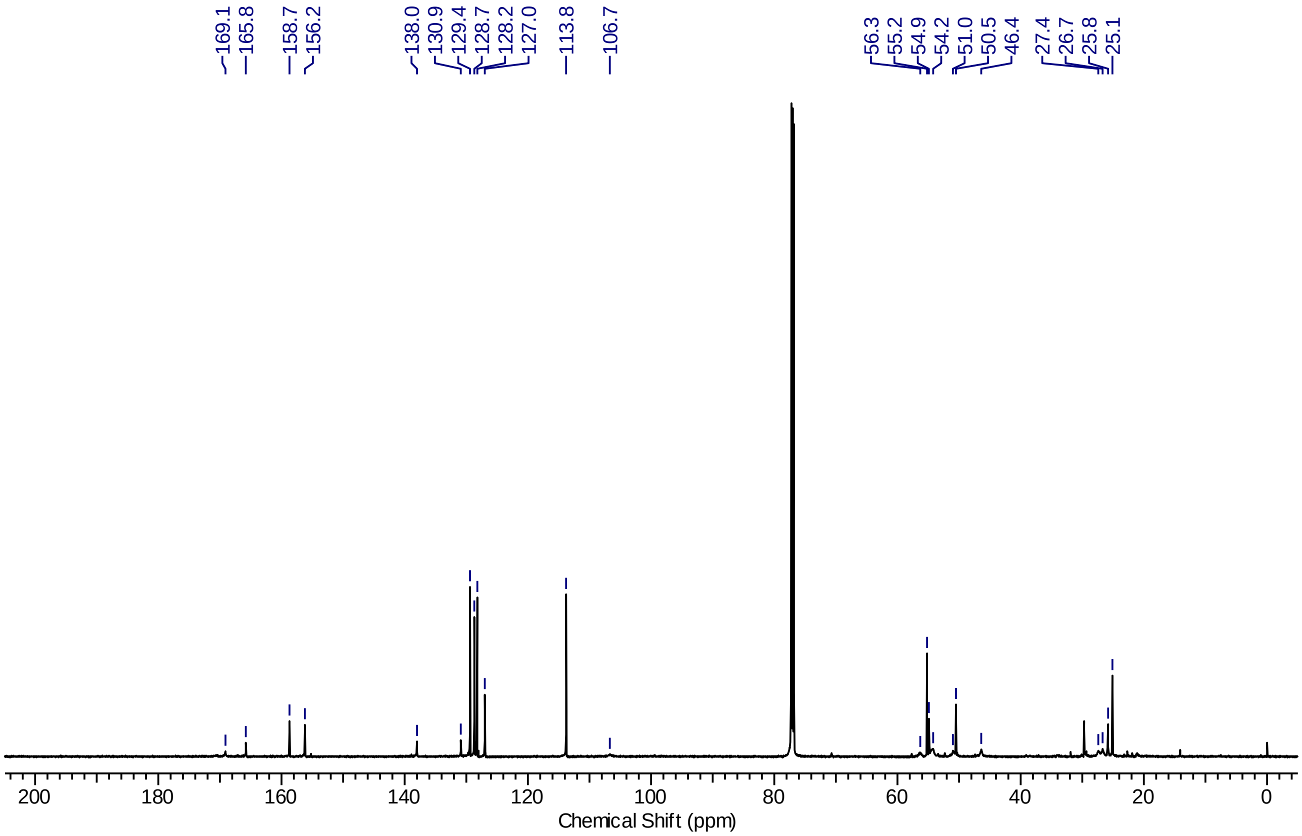
169.1
165.8
158.7
156.2
138.0
130.9
129.4
128.7
128.2
127.0
113.8
106.7
56.3
55.2
54.9
54.2
51.0
50.5
46.4
27.4
26.7
25.8
25.1
200
180
160
140
120
100
80
60
40
20
0
Chemical Shift (ppm)

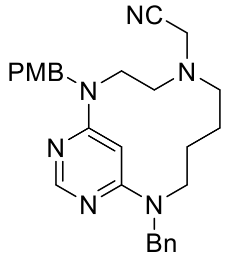
NC
PMB
N
N
N
N
N
Bn

5a

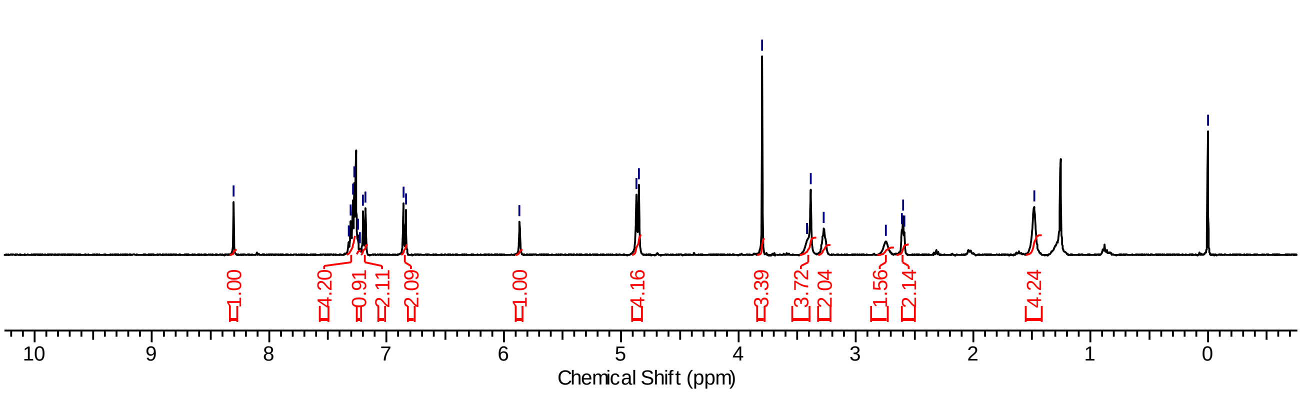
8.302
7.323
7.306
7.287
7.273
7.244
7.226
7.201
7.180
6.856
6.835
5.866
4.871
4.850
3.798
3.414
3.386
3.274
2.744
2.609
2.599
2.587
1.481
0.000
1.00
4.20
0.91
2.11
2.09
1.00
4.16
3.39
3.72
2.04
1.56
2.14
4.24
Chemical Shift (ppm)

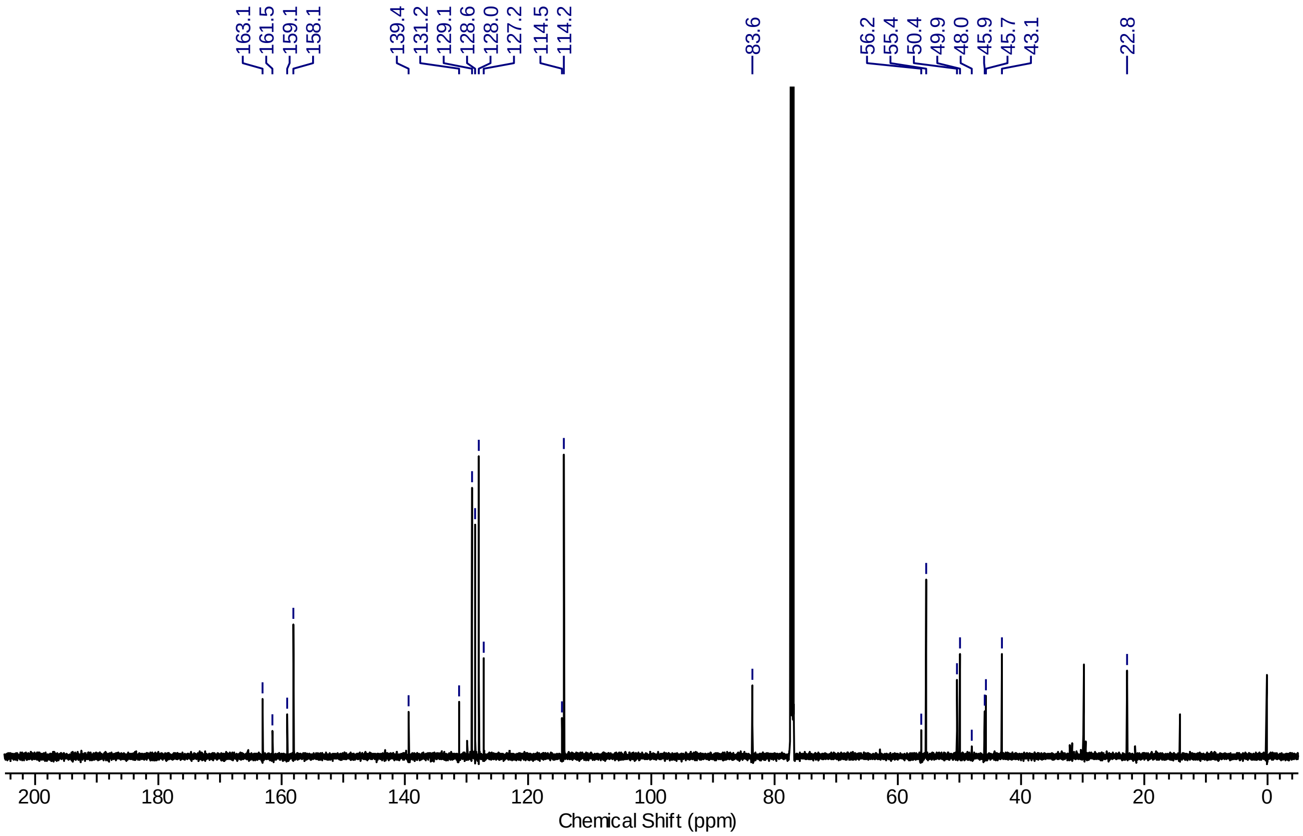
163.1
161.5
159.1
158.1
139.4
131.2
129.1
128.6
128.0
127.2
114.5
114.2
83.6
56.2
55.4
50.4
49.9
48.0
45.9
45.7
43.1
22.8
Chemical Shift (ppm)

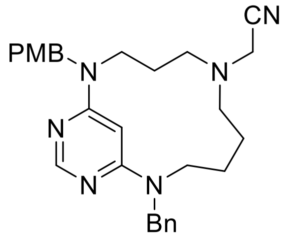
CN
PMB
N
N
N
N
N
N
Bn

5b

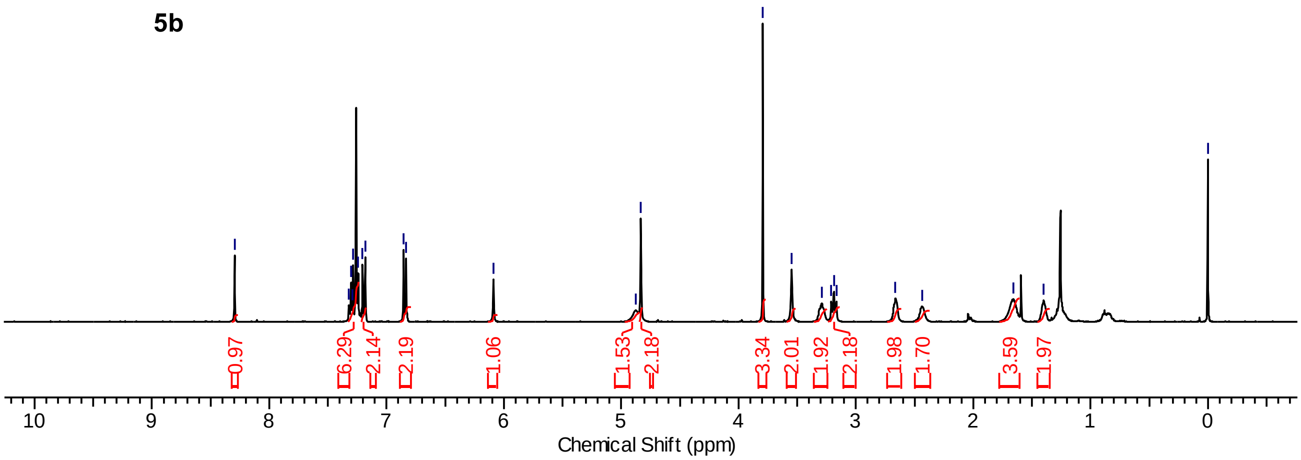
8.294
7.321
7.303
7.301
7.293
7.285
7.242
7.205
7.183
6.853
6.832
6.089
4.874
4.832
3.793
3.546
3.291
3.209
3.188
3.166
2.663
2.435
1.657
1.400
0.000
0.97
6.29
2.14
2.19
1.06
1.53
2.18
3.34
2.01
1.92
2.18
1.98
1.70
3.59
1.97
10
9
8
7
6
5
4
3
2
1
0
Chemical Shift (ppm)

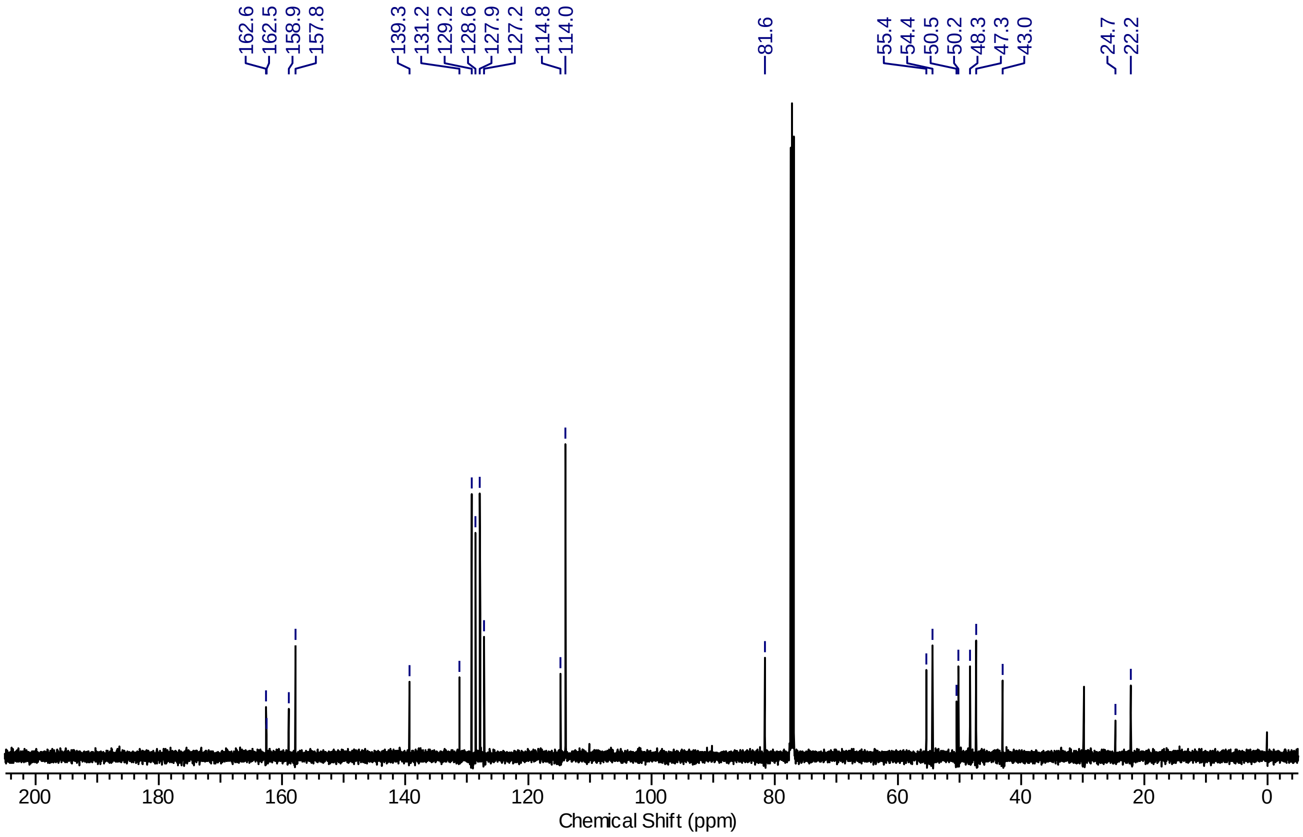
162.6
162.5
158.9
157.8
139.3
131.2
129.2
128.6
127.9
127.2
114.8
114.0
81.6
55.4
54.4
50.5
50.2
48.3
47.3
43.0
24.7
22.2
200
180
160
140
120
100
80
60
40
20
0
Chemical Shift (ppm)

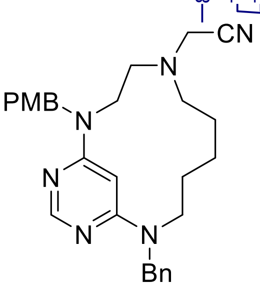
CN
N
PMB
N
N
N
N
Bn

5c

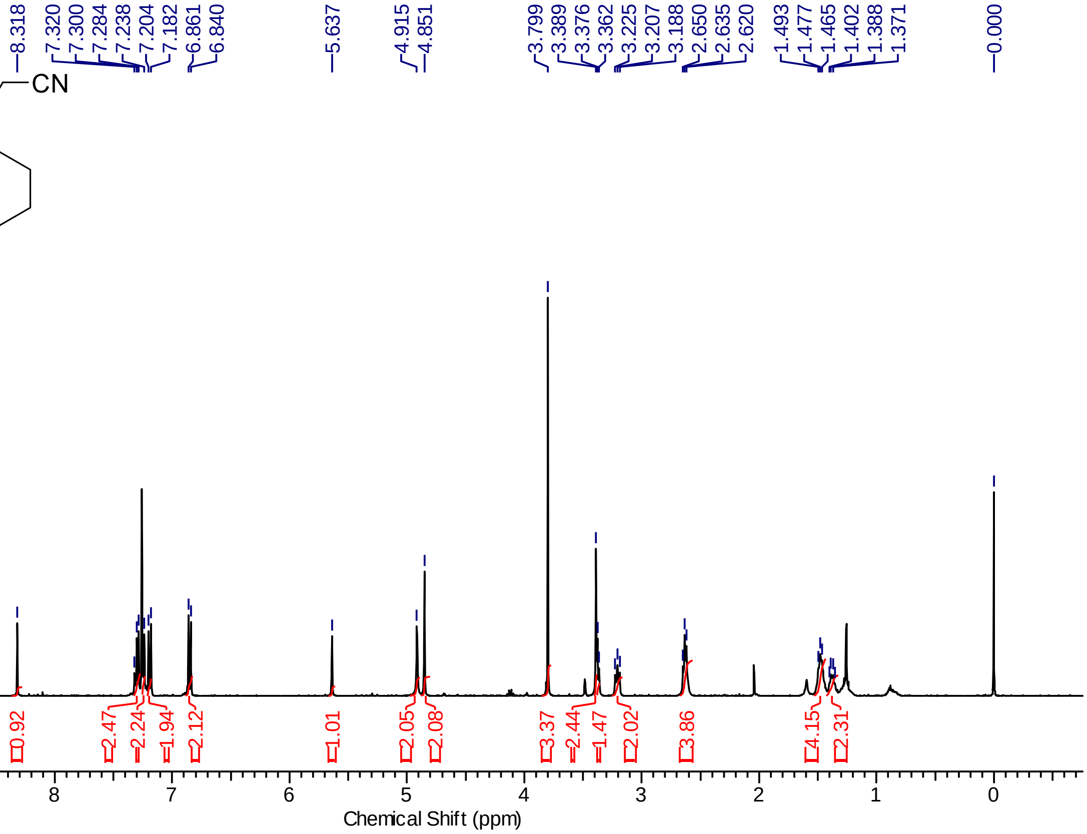
8.318
7.320
7.300
7.284
7.238
7.204
7.182
6.861
6.840
5.637
4.915
4.851
3.799
3.389
3.376
3.362
3.225
3.207
3.188
2.650
2.635
2.620
1.493
1.477
1.465
1.402
1.388
1.371
0.000
0.92
2.47
2.24
1.94
2.12
1.01
2.05
2.08
3.37
2.44
1.47
2.02
3.86
4.15
2.31
10
9
8
7
6
5
4
3
2
1
0
Chemical Shift (ppm)

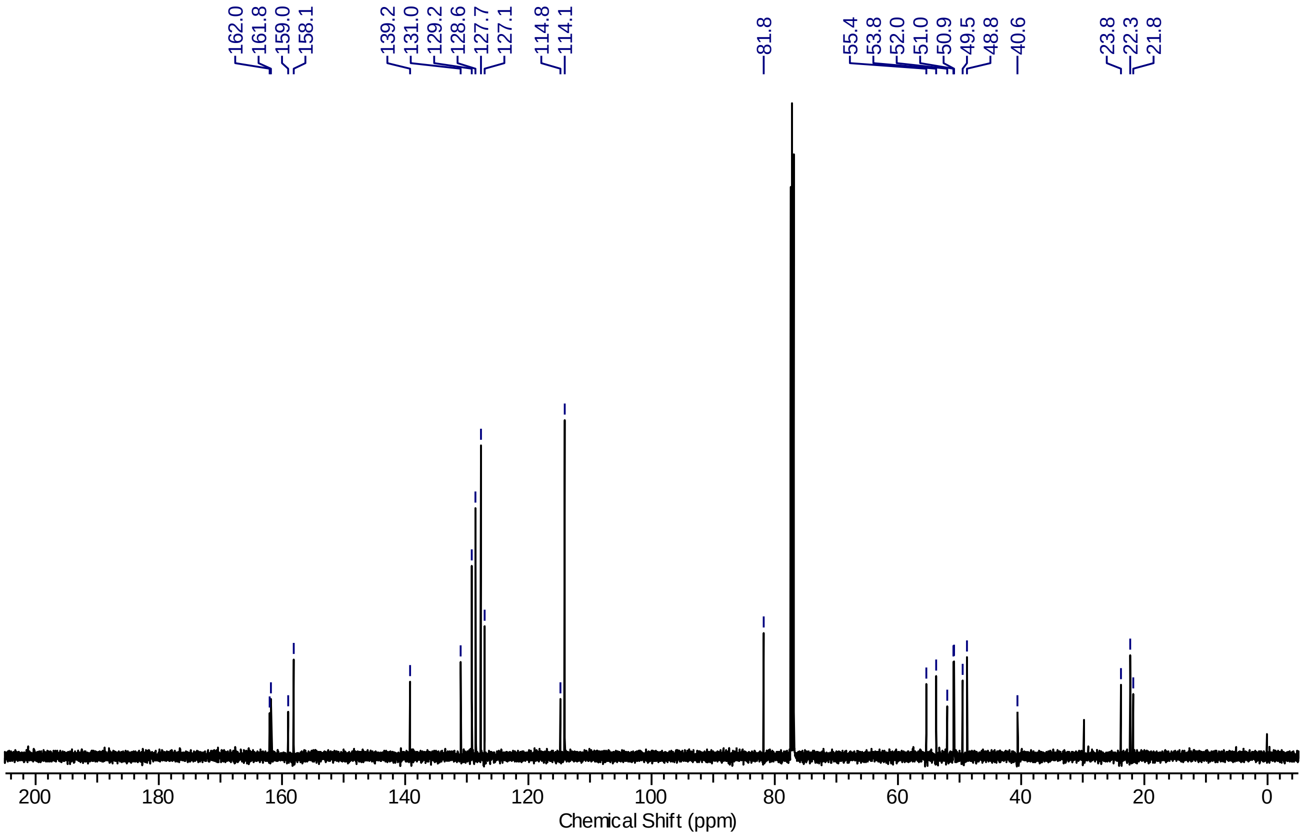
162.0
161.8
159.0
158.1
139.2
131.0
129.2
128.6
127.7
127.1
114.8
114.1
81.8
55.4
53.8
52.0
51.0
50.9
49.5
48.8
40.6
23.8
22.3
21.8
200
180
160
140
120
100
80
60
40
20
0
Chemical Shift (ppm)

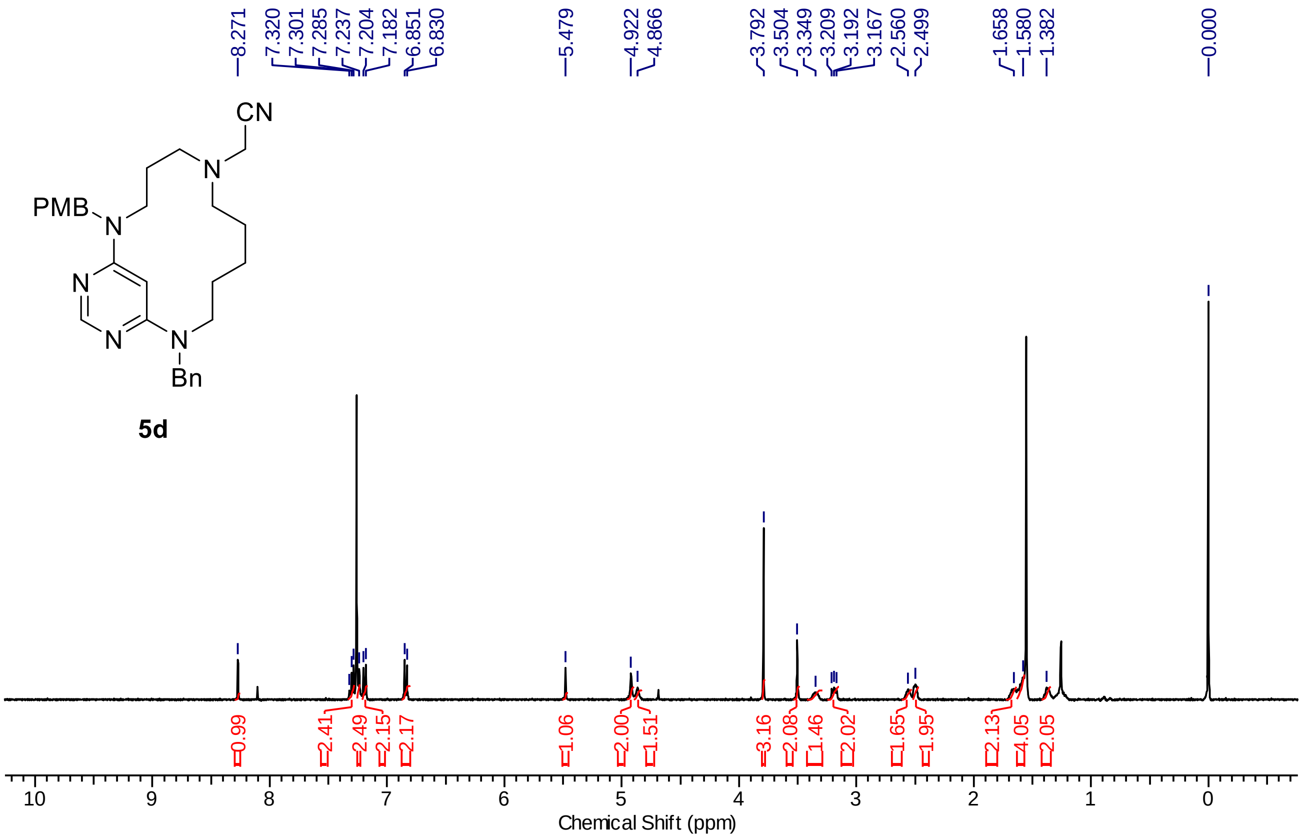
CN
N
PMB
N
N
N
N
Bn
5d
8.271
7.320
7.301
7.285
7.237
7.204
7.182
6.851
6.830
5.479
4.922
4.866
3.792
3.504
3.349
3.209
3.192
3.167
2.560
2.499
1.658
1.580
1.382
0.000
0.99
2.41
2.49
2.15
2.17
1.06
2.00
1.51
3.16
2.08
1.46
2.02
1.65
1.95
2.13
4.05
2.05
Chemical Shift (ppm)

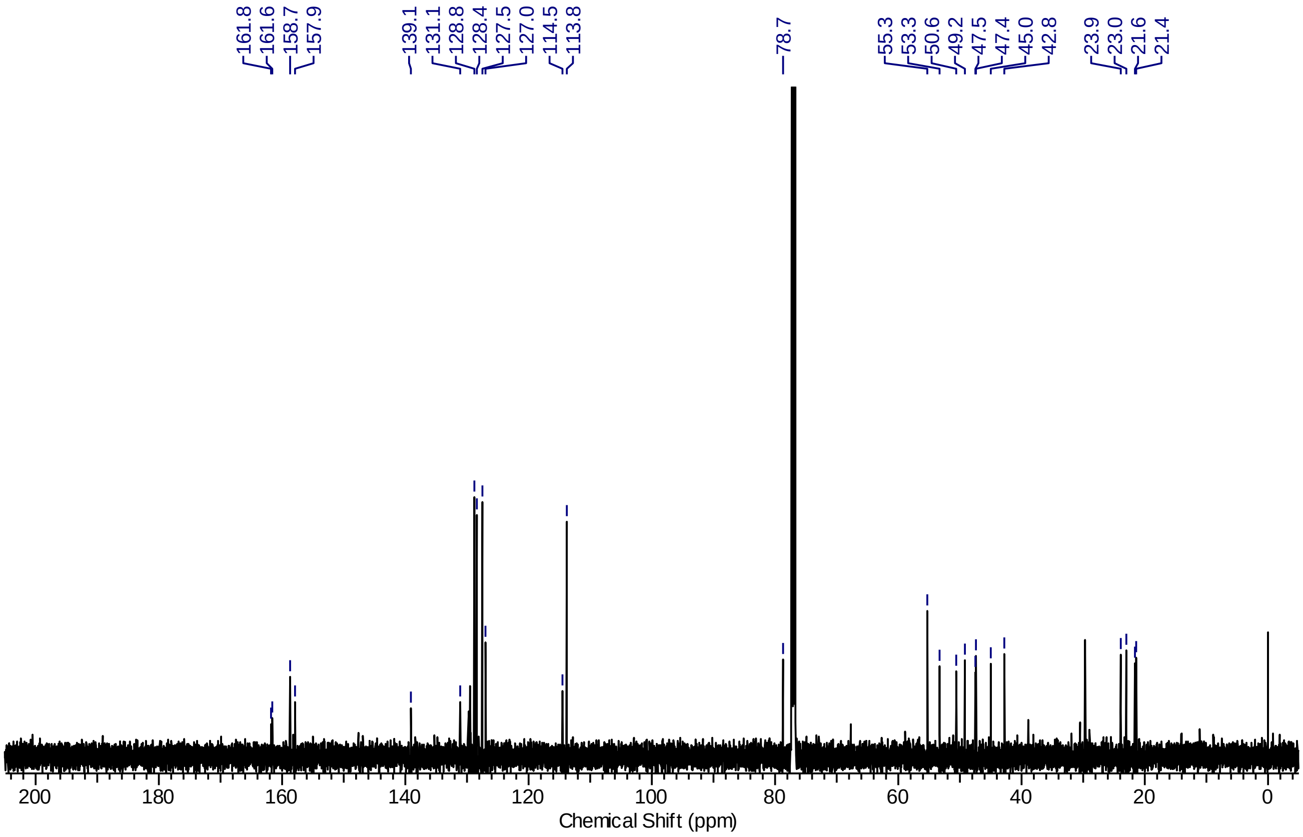
161.8
161.6
158.7
157.9
139.1
131.1
128.8
128.4
127.5
127.0
114.5
113.8
78.7
55.3
53.3
50.6
49.2
47.5
47.4
45.0
42.8
23.9
23.0
21.6
21.4
Chemical Shift (ppm)

2 References

Choi, Y., Kim, H., Shin, Y.-H., and Park, S. B. (2015). Diverse Display of Non-Covalent Interacting Elements using Pyrimidine-Embedded Polyheterocycles. *Chem. Commun.* 51(65)**,** 13040–13043. doi: 10.1039/C5CC04335B.

Fürstner, A., Bouchez, L. C., Morency, L., Funel, J.-A., Liepins, V., Porée, F.-H., et al. (2009). Total Syntheses of Amphidinolides B1, B4, G1, H1 and Structure Revision of Amphidinolide H2. *Chem. Eur. J.* 15(16)**,** 3983–4010. doi: 10.1002/chem.200802067.
